# A resource-rational model of human processing of recursive linguistic structure

**DOI:** 10.1073/pnas.2122602119

**Published:** 2022-10-19

**Authors:** Michael Hahn, Richard Futrell, Roger Levy, Edward Gibson

**Affiliations:** ^a^Department of Linguistics, Stanford University, Stanford, CA 94305;; ^b^Collaborative Research Center 1102, Saarland University; Saarland 66123, Germany;; ^c^Department of Language Science, University of California, Irvine, CA 92697;; ^d^Department of Brain and Cognitive Sciences, Massachusetts Institute of Technology, Cambridge, MA 02139

**Keywords:** language processing, surprisal, resource-rationality

## Abstract

A major goal of psycholinguistic theory is to account for the cognitive constraints limiting the speed and ease of language comprehension and production. Wide-ranging evidence demonstrates a key role for linguistic expectations: A word’s predictability, as measured by the information-theoretic quantity of surprisal, is a major determinant of processing difficulty. But surprisal, under standard theories, fails to predict the difficulty profile of an important class of linguistic patterns: the nested hierarchical structures made possible by recursion in human language. These nested structures are better accounted for by psycholinguistic theories of constrained working memory capacity. However, progress on theory unifying expectation-based and memory-based accounts has been limited. Here we present a unified theory of a rational trade-off between precision of memory representations with ease of prediction, a scaled-up computational implementation using contemporary machine learning methods, and experimental evidence in support of the theory’s distinctive predictions. We show that the theory makes nuanced and distinctive predictions for difficulty patterns in nested recursive structures predicted by neither expectation-based nor memory-based theories alone. These predictions are confirmed 1) in two language comprehension experiments in English, and 2) in sentence completions in English, Spanish, and German. More generally, our framework offers computationally explicit theory and methods for understanding how memory constraints and prediction interact in human language comprehension and production.

## Significance

Researchers have long studied humans’ difficulty in comprehending complex sentences as a window into the nature of human language processing. However, a unified theoretical account of comprehension difficulty has proven elusive. We propose a unifying model based on resource-rational memory representations, which we scale to the rich statistical structure of language using large-scale text data and contemporary machine learning methods. The model makes fine-grained predictions sharply different from those of existing models, which we confirm in three behavioral experiments. Taken together, our work shows how general cognitive principles, implemented using machine learning, predict fine-grained patterns in human language comprehension that previous theories cannot account for.

Language expresses recursive thoughts via linear strings of words (1). Therefore, a central part of language comprehension is recovering a hierarchical structure from a linear sequence. While we do this seemingly without effort, it has long been observed that humans’ ability to do so can run against limitations of short-term memory (2). Such limitations are of central importance to understanding the nature of human language processing, and have been an important subject of study (3–8). Human processing limitations often give rise to measurable, localized differences in comprehension difficulty between otherwise similar sentences, and modeling those difficulty differences has been a key aim of psycholinguistic research (3–8). However, it has proven challenging to develop a unified account of what makes different sentences easier or harder for humans to comprehend.

Research has identified two seemingly disparate perspectives on what makes sentences hard to comprehend. Expectation-based models (9, 10) describe how context generates expectations about likely future input. According to such models, words are harder to process when they are harder to anticipate from preceding context. In contrast, memory-based models hold that difficulty of processing stems from limits on the ability to store representations of preceding context and to retrieve and integrate them with new input (4, 5, 7). Both perspectives are supported by substantial bodies of empirical evidence (11–16), and it has remained an open question how they can be theoretically and empirically reconciled (14–16).

Here, we develop a theory and implemented model reconciling expectation-based and memory-based theories, building on recent research that has proposed a key role for noise and uncertainty in modeling human mental representations of linguistic input (17–19). Whereas traditional models of language processing generally assume veridical context and input representations—the problem of sentence-level comprehension is cast as one of analyzing a known sequence of words to determine its structure and meaning and to predict future input—“noisy channel” language processing theory treats these representations as uncertain, and hypothesizes that analysis and prediction in human language processing approximates normative principles of Bayesian inference given these uncertain representations (20, 21). These ideas have led to the proposal of unifying expectation-based and memory-based theories of processing difficulty through lossy-context surprisal (22). Lossy-context surprisal posits that human processing difficulty is determined by expectations derived not from veridical context but from probabilistic inference over imperfect memory representations of the context. In principle, this approach could account for the predictions of both expectation-based and memory-based models: Words are easy to process when they are easy to anticipate—as predicted by expectation-based models—but if the relevant contextual information is poorly represented in memory, upcoming words may be difficult to anticipate correctly, yielding processing difficulty as predicted by traditional memory-based theories. However, to date, many parts of this theory remain to be specified. The theory lacks an implemented specification of which aspects of preceding context are prone to memory loss, which is key to deriving testable predictions. Ideally, this specification should be based on deeper theoretical principles. Furthermore, no scaled implementation of noisy channel processing, which is necessary to make fine-grained predictions on the difficulty profiles of specific sentences, has been available.

In this work, we present theoretical and empirical advances that address these limitations. On a theoretical level, we propose a resource-rational model (23) of fine-grained memory representations, based on the hypothesis that memory representations are optimized to minimize expected downstream processing effort given cognitive resource constraints. Combining this idea with lossy-context surprisal as a processing difficulty metric leads to wide-ranging empirical predictions. In order to evaluate those predictions and understand them in detail, we implement the proposed model using contemporary neural network modeling, and fit it on large-scale text data, enabling the theory to make detailed predictions regarding human comprehension behavior for arbitrary natural language input.

**Fig. 1.**
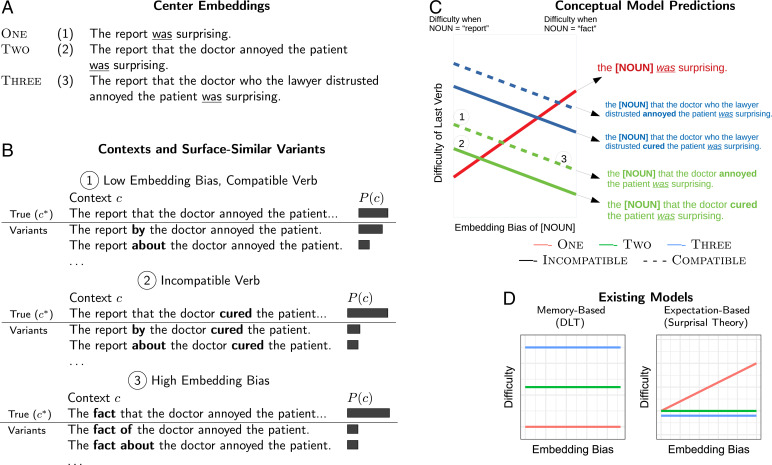
(*A*) Sentences exhibiting nested recursive structures (“center embeddings”), with one, two, or three noun–verb pairs. Difficulty differences between the sentences manifest as reading time differences on the underlined verbs. (*B*) Varying the probability of true contexts and surface-similar variants: We show a context requiring a verb in order to form a complete sentence, with surface-similar variants that do not require a verb in order to be complete (item 1). The relative prior probability *P*(*c*) of the true context and the surface-similar variants can be modulated by changing the identity of the second-to-last verb (item 2) or the first noun (item 3). Our model predicts that the final verb is easier to process when the true context has higher prior probability when compared to the variants. (*C*) Conceptual predictions of our model for difficulty at the last verb: First, higher difficulty is predicted for the Three condition (blue) than the Two condition (green). Second, higher difficulty is predicted when the second-to-last verb is semantically compatible (*“*annoyed*”* as opposed to *“*cured*”*) with the first noun. Third, higher difficulty is predicted when the first noun has a lower embedding bias (*“*report*”* as opposed to *“*fact*”*). In the One condition, an effect in the opposite direction is predicted. (*D*) Predictions are sharply different from existing theories: The DLT predicts that increasing levels of embedding should increase difficulty, but predicts no effects of embedding bias or compatibility. Surprisal theory predicts that embedding bias impacts difficulty in the One condition, but not in the other conditions.

Our theory derives predictions for difficulty patterns in human processing of recursive structures that neither expectation-based nor memory-based theories individually could account for. Recursive structures, in particular, cases of center embedding where sentences are nested inside one another, are crucial for psycholinguistic theory because they reveal human limitations in processing the hierarchical structures of language (2–4, 24–26). Consider Fig. 1*A*. In these sentences, varying numbers of sentences are embedded within each other. More center embedding leads to structures that are more difficult to process: Whereas items 1 and 2 in Fig. 1*A* are readily understood, item 3 is considerably harder. Adding further levels of embedding would increase difficulty to the point of incomprehensibility. More levels of center embedding are rarer in language use (27), so purely expectation-based theories correctly predict that they are difficult overall, but fail to predict where this difficulty manifests in human processing: when exiting the embedding, at the word “was” in the examples of Fig. 1*A*. If context were veridically represented and used to predict upcoming input, then exiting the embedding at this point should be exactly what is expected, and easy for human language processing.

Some memory-based theories predict that exiting the embedding is difficult, on the basis that the complexity of the preceding context makes retrieval of the correct site for structural integration challenging (4, 5, 7). Here, however, we present experimental work showing that this difficulty is modulated by fine-grained differences in the context: for example, changing *report* to *fact* in the sentences of Fig. 1*A* turns out to make exiting the center embedding easier. This phenomenon is not predicted by existing memory-based theories.

Our model is capable, in principle, of accounting for all these patterns. When memory representations are imperfect, rational comprehenders should reconstruct the context based on their knowledge of the statistics of the language. Comprehenders’ structural expectations of inputs should thus be biased toward contexts with high a priori probability that are similar in form to the true contexts. In a resource-rational model, this will particularly affect variants that differ in words that are normally easy to reconstruct from other parts of the context, such as high-frequency function words. For instance, we expect that a context such as “the report that the doctor annoyed the patient*…*” will compete with variants such as “the report by the doctor annoyed the patient*…*,” where “annoyed” is the verb belonging to the initial noun “report.” For such a nonveridical variant, no third verb is expected. Rational comprehenders with imperfect memory should thus be more likely to expect the final verb when such nonveridical versions with lower embedding depth have a lower a priori probability. In contrast, when nonveridical variants have high a priori probability, comprehenders should not expect the final verb, and comprehension will be disrupted when it is encountered.

These expectations can be measured via native speakers’ reading times when they encounter the final verb after the preceding context, or, alternatively, by providing native speakers with the preceding context and asking them to complete the sentence. We use both to test our theory. First, we show that a scaled-up implementation of our model indeed derives the predictions intuitively described above. Next, in two reading time experiments, we systematically vary the a priori probability of the true context relative to structurally different variants. We find that, when the prior favors nonveridical variants, humans experience increased difficulty on processing the final verb. This contrast is a signature prediction of our proposed unifying model, and does not follow from existing models from either the expectation-based or the memory-based paradigms. Finally, in a production study in three languages (English, German, and Spanish), we then show an analogous pattern in production, whereby humans are more likely to produce the correct number of verbs when structurally different variants have a lower a priori probability.

## Formalization and Implementation

**Fig. 2.**
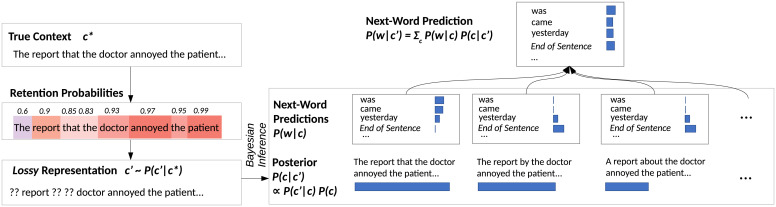
Resource-rational lossy-context surprisal. We show the model as applied to a context ($c^{*}$) exhibiting a center embedding, and requiring a subsequent verb phrase (e.g., “was surprising”) to form a complete sentence. The model determines retention probabilities for every word, varying with the number of intervening words. These, together, determine a distribution over lossy representations c′, for which we show one sample where three words have been lost. Combining knowledge of the retention probabilities with the a priori statistics of the language, Bayesian inference determines a posterior distribution over likely true contexts, given the lossy representation. Each possible context leads to a distribution over possible next words. For example, the first context is likely to be followed by a verb; the second and third ones are not. The model posits that human comprehenders’ expectations of the next word arise from marginalizing out the possible contexts *c*. In this example, substantial probability is assigned to continuations without a verb, increasing comprehension difficulty when a verb is encountered.

We describe the proposed model, resource-rational lossy-context surprisal, in Fig. 2. The model computes a retention probability (28, 29) for each word in the past context, determined by 1) the word’s identity and 2) how many words have been observed after observing it. The overall memory representation c′ then consists of the available words, and a placeholder symbol for those words that have not been retained. Via Bayes’ rule and knowledge of the a priori statistics of the language, c′ gives rise to a posterior P(c|c′) over possible contexts and thus a predictive distribution P(w|c′) over the next word. Processing difficulty on a word is determined by its degree of unpredictability from c′, as measured by the information-theoretic quantity of surprisal,

[1] $$-\log P(w|c\prime )=-\log\sum_{c}P(w|c)P(c|c\prime ).$$

We represent the a priori statistics of English using GPT-2 (30), a large-scale neural network model that provides one of the strongest existing statistical models of English text. Retention probabilities are parameterized using a neural network acting on a context-independent vector representation of the word and its distance. We optimize retention probabilities to minimize the average model surprisal [**1**] over large-scale text data, subject to an upper bound on the average number of retained context words. We fitted the model for integer values of this bound from 0 to 20. Optimization uses text data from the English Wikipedia, unrelated to the center embeddings stimuli of interest (see *Materials and Methods*). The optimized retention probabilities prominently exhibit two key properties: Words are more likely to be preserved when they are recent, and when they have lower word frequency (*SI Appendix*, Fig. S2). Both biases are well documented in experimental research on human linguistic memory (31–33).

## Predictions

We used resource-rational lossy-context surprisal to derive three predictions about the processing of nested recursive structures (Fig. 1 *B* and *C*). First, recovering from more levels of embedding should be harder (blue vs. green lines in Fig. 1*C*). This simple prediction is common to most models of memory in sentence comprehension, going back to the 1960s (2, 4, 7, 24). Our model generally predicts it because more levels of embedding are less likely a priori, so that nonveridical variants with fewer levels tend to have higher a priori probability.

Second, recovering should be easier when semantic cues reinforce the correct dependency structure for the preceding embedding context. We tested this prediction by manipulating whether the second-to-last verb was compatible with both the first and second nouns as its subject, as in Fig. 1*B*, item 1 (report … annoyed and doctor … annoyed are both plausible), or with only the second noun, as in Fig. 1*B*, item 2 (report … cured is implausible, whereas doctor … cured is plausible). When this verb is incompatible with the first noun as its subject, it disfavors nonveridical context representations such as “the report by the doctor cured the patient*…*” and thus reinforces the veridical context. Therefore, changing the verb “annoyed” to “cured” should decrease processing effort on the final verb. Early work on center embedding already noted that semantic match between nouns and verbs made comprehension easier (25, 26, 34), but, as far as we know, this effect has not been demonstrated in word-by-word reading times. While such an effect may be compatible with some memory-based theories (7, 35), it does not arise in existing computationally explicit models (7).

Third, recovering should be easier when the nouns provide supporting statistical cues (Fig. 1*B*, item 3). Nouns vary strongly in the a priori probability that they are followed by a “that”-clause; this probability ranges from ≈70% (“fact”) to ≈0.7% (“report”). We use the term “embedding bias” to denote the log-probability that a noun is followed by “that.” In Fig. 1*B*, item 3, changing the noun “report” to “fact” decreases the probability of the nonveridical variants, which is again predicted to decrease processing effort on the final verb. In the Two and Three conditions, the third verb should be predicted more accurately when embedding bias is higher, as it increases the a priori probability of the true context (descending blue and green lines in Fig. 1*C*). This prediction does not follow from existing models but is a straightforward consequence of our model. In the One condition, we expect the opposite pattern (ascending red line in Fig. 1*C*), as nouns that embed a clause with a very high probability are less likely to be immediately followed by a verb.

The resulting pattern of difficulty is sharply different from what is predicted by existing memory-based and expectation-based models (Fig. 1*D* and *SI Appendix*, section S7.1). We exemplify these using the dependency locality theory (DLT) (36), which asserts that difficulty stems from integrating long syntactic dependencies, and surprisal theory (9, 10), which asserts that processing effort is proportional to surprisal derived from fully veridical context representations. The effect of the number of embedding levels, and the behavior of the One condition, are predicted by existing memory-based and expectation-based theories, respectively. However, neither group of previous models predicts effects of semantic compatibility or embedding bias in the Two and Three conditions. See *SI Appendix*, section S7 for more on previous models.

## Experiment 1: Effect of Statistical Cues

We tested resource-rational lossy-context surprisal by comparing model surprisal (Eq. **1**) against human processing difficulty on the final verb, as reflected in reading times.

**Fig. 3.**
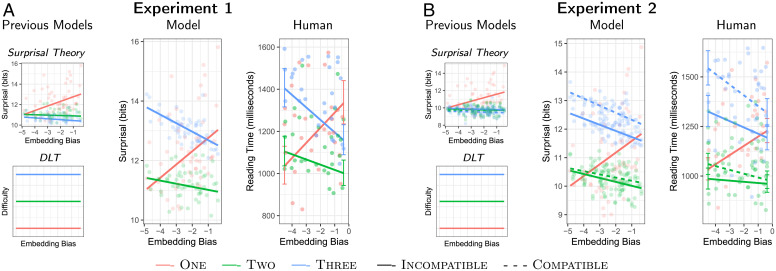
Model predictions and reading times in Experiment 1 (*A*) and Experiment 2 (*B*). We compare human reading times with predictions from our model, and with the predictions of previous theories of processing difficulty. For the human data, we show empirical per-noun means, and estimated reading times obtained from a trial-by-trial Bayesian mixed-effects analysis (see *Materials and Methods*), with posterior SDs for reading times at “report” (left end, low embedding bias) and “fact” (right end, high embedding bias). The difficulty pattern predicted by the model, distinct from the predictions of previous theories, is borne out in the human reading time data.

We constructed 32 stimuli of the form in Fig. 1*A*, each in three conditions (One, Two, and Three), and crossed these with 58 different nouns varying in embedding bias (e.g., report, fact, …). We first derived predictions for surprisal theory and DLT for difficulty on the final verb (Fig. 3*A*); we implemented surprisal theory using the same statistical model used for the prior in our model (GPT-2). We then computed resource-rational lossy-context surprisal, varying the average number of context words retained during optimization of the retention probabilities from 0 to 20. Across the range of this parameter, the model traverses three distinct phases (*SI Appendix*, Fig. S4): When many (≳17) words are retained, behavior is very similar to surprisal theory. When very few (≲4) words are retained, model surprisal is indistinctly flat in Two and Three. In between, the model exhibits the qualitative predictions described above; we show results for an average retention rate of 10 words in Fig. 3*A* (see *SI Appendix*, Fig. S4 for results at other retention rates): Model surprisal is higher in the Three condition than in Two, increases with embedding bias in the One condition, and decreases with embedding bias in the Two and Three conditions.

We compared these predictions to human reading times, which we measured using the maze paradigm (37–39). In this paradigm, participants read sentences word by word, and choose between the correct next word and a distractor word that is matched in length and frequency but is very bad in the given context. The dependent variable of interest is the time it takes to make a choice (reaction time). The maze paradigm enables higher temporal resolution and sensitivity than the traditional self-paced reading paradigm, which suffers from poor localization due to spillover effects and from noise due to inattentive participants, in particular, in web-based experiments (38, 39). Different methods exist for creating distractor words; we use the A-maze variant in which distractors are automatically generated using large-scale statistical language models (39) (see *Materials and Methods* for details).

We recruited 100 English native speakers, collecting data from 10 critical trials for each participant, interspersed with 30 fillers (see *Materials and Methods* for details).

The median participant made an error on 1.9% of words across both fillers and critical trials. The predetermined exclusion criterion for participants (incorrect response on ≥20% of words) affected 1.0% of participants. We excluded trials from reading time analyses when the response on the critical word was wrong; this affected 5.2% of the data (primarily in the difficult Three condition). See *SI Appendix*, section S3.6 for analyses of errors and of reading times conditioned on errors.

We show reading times on the last verb in Fig. 3*A*. We analyzed these, after log-transformation, in a Bayesian linear mixed-effects regression, entering participants, items, and nouns as random effects (see *Materials and Methods* for details). The results exhibit the effects predicted by the resource-rational model. First, reading times were higher in Three than in Two (β=0.18, 95% credible interval [CrI] [0.13,0.25], P(β<0)<0.0001, effect in raw reading times: 217 ms, 95% CrI [144,297] ms). Second, there was an interaction between embedding bias and the presence of a “that”-clause (β=−0.09, 95% CrI [−0.15,−0.03], P(β>0)=0.0015). Consistent with both our model and surprisal theory, the effect of embedding bias was positive in the One condition (difference between “fact” and “report”: 297 ms, 95% CrI [34,566] ms). However, as predicted by our model, this effect turned negative in the presence of a “that”-clause (difference between “fact” and “report”: –166 ms, 95% CrI [−297,−41] ms). This effect agrees with model predictions, and is inconsistent with DLT or surprisal theory. An interaction between embedding depth and embedding bias can be demonstrated when pooling data across experiments (*SI Appendix*, sections S2.1 and S6.6). See *SI Appendix*, section S3 for further analyses.

We found that resource-rational lossy-context surprisal improves over surprisal theory and DLT in predicting reading times not only in center embeddings but also in the filler trials (*SI Appendix*, section S9).

We also examined predictions for a uniform memory model where each context word is retained with equal probability, assumed in prior work on lossy-context surprisal (22). This model predicts the effects of embedding bias but not the effect of depth. We further compared to a window-based model where exactly the last *K* words are available; this model predicts the effect of depth but not the negative effect of embedding bias. See *SI Appendix*, section S2.2 for details.

## Experiment 2: Effect of Semantic Cues

We next replicated experiment 1 on a second set of items, and simultaneously tested the predicted effect of semantic compatibility.

Beyond the two manipulations from experiment 1, in the Two and Three conditions, we additionally varied the second-to-last verb phrase: In the Compatible condition, the first noun was a plausible subject (e.g., “annoyed the patient”); in the Incompatible condition, it was not (e.g., “cured the patient”). In the Compatible condition, nonveridical versions such as “the report by…” should have a higher a priori probability, making prediction of the last verb less accurate. We constructed 42 stimulus items.

Fig. 3*B* shows predictions from the resource-rational model and previous theories for these items. In addition to the effects from experiment 1, the model predicts higher difficulty in the Compatible condition, particularly within Three. Neither surprisal theory nor DLT predict any effect of compatibility.

We collected reading time data from 200 participants, including both Compatible and Incompatible variants in the Two and Three conditions. In all other respects, experiment and data analysis were identical to experiment 1. Reading times are shown in Fig. 3*B*. The results of experiment 1 were replicated: First, reading times were higher in Three than in Two (β=0.29, 95% CrI [0.24,0.35], P(β<0)<0.0001; effect in raw reading times: 337 ms, 95% CrI [267,411] ms). Second, there was an interaction between embedding bias and the presence of a “that”-clause (β=−0.06, 95% CrI [−0.10,−0.024], P(β>0)=0.0007). As in experiment 1, the effect of embedding bias was positive in the One condition (difference between “fact” and “report”: 193 ms, 95% CrI [37,357] ms), and negative across the Two and Three conditions (difference between “fact” and “report”: –105 ms, 95% CrI [−194,−18] ms). Third, in agreement with the model predictions, reading times were higher in the Compatible condition than the Incompatible condition (β=0.083, 95% CrI [0.031,0.136], P(β<0)=0.0014; effect in raw reading times: 96 ms, 95% CrI [36,156] ms). See *SI Appendix*, section S3 for further analyses. Note that the effects of embedding bias and compatibility are numerically larger in the Three condition than in the Two condition; a metaanalysis shows that these differences are statistically meaningful in both reading times and in parts of the model’s parameter space (*SI Appendix*, sections S2.1 and S6.6). Numerical differences in the slope of embedding bias between Compatible and Incompatible were not statistically meaningful (*SI Appendix*, Fig. S23), nor were numerical differences in the intercept of the model predictions between the two experiments (*SI Appendix*, Fig. S6).

See *SI Appendix*, section S6 for converging evidence from preceding reading time studies (total *n* = 501). We further replicated the effect of embedding bias on comprehension in two ratings studies (total *n* = 335; *SI Appendix*, section S5).

## Experiment 3: Production Study

So far, we have confirmed the model predictions in reading times. Difficulty measured in reading times indicates that humans’ expectations are violated, but does not directly indicate what human expectations are. To provide a second test of human expectations, we turned to a production paradigm—Cloze completion (40, 41)—that has been used in language research in order to evaluate what words are expected immediately following a preamble. We use this method in order to evaluate the complexity of multiply nested structures, in order to measure how many verbs humans expect following a complex preamble.*

**Fig. 4.**
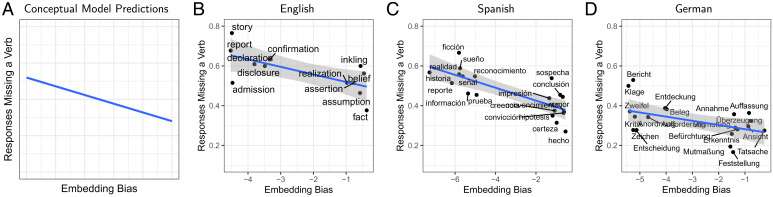
Production experiments. (*A*) Conceptually, the model predicts that incomplete responses are most common for nouns with low embedding bias. In contrast, neither surprisal theory nor DLT predict any effect of embedding bias. (*B*–*D*) While languages differ in the overall rate of ungrammatical responses, the prediction is borne out in each language. Blue lines indicate linear fits; gray shading indicates 95% confidence bands.

We asked participants to complete contexts of the form “The report that the doctor who the diplomat*…*” to a complete sentence. We expected participants to either produce grammatical completions with three verbs, such as “*…*mistrusted cured the patient was surprising,” or ungrammatical versions with fewer verbs, such as “*…*mistrusted was surprising.” Resource-rational lossy-context surprisal predicts that the rate of such ungrammatical completions should be lower for nouns with high embedding bias (e.g., “fact”), as these make it easier to recover the true context from imperfect memory representations (Fig. 4*A*). Existing expectation-based and memory-based models do not predict that the rate of grammatical completions depends on embedding bias.

We recruited 80 participants. Fig. 4 shows the rate of incomplete completions (less than three verbs) as a function of embedding bias. As predicted, there was an effect of embedding bias on the rate of ungrammatical responses (β=−0.32, 95% CrI [−0.60,−0.05], P(β>0)=0.0123) in a trial-by-trial logistic mixed-effects analysis.

We replicated this study in two more languages (Spanish and German), including one (German) where the difficulty of center embeddings has been found to be substantially weaker than in English (42). In Spanish, we targeted subject relative clauses (*el hecho de que el director que*, “the fact that the director who”) to avoid less natural subject–initial object relative clauses, simultaneously testing generalization to a different syntactic configuration. In German, we targeted embedded structures (e.g., *Klaus hat erzählt, dass die Behauptung, dass der Student, den der Professor*, “Klaus said that the claim that the student who the professor”), as they are known to increase difficulty to levels closer to English (35).

We recruited 60 participants in each language. In both languages, the effect of embedding rate was estimated to be negative, with estimated effect sizes comparable to the English result (Spanish: β=−0.23, 95% CrI [−0.34,−0.12], P(β>0)<0.0003; German: β=−0.28, 95% CrI [−0.56,−0.03], P(β>0)=0.01738). These results suggest that the—previously undocumented—effect of embedding bias on human expectations holds across different languages, even when they vary in the overall difficulty of center embeddings.

## Discussion

We have introduced a model of human language processing as resource-rational prediction, scaled to arbitrary input using contemporary machine learning methods. Aiming to reconcile memory- and expectation-based perspectives on human syntactic processing, the model not only recovers predictions of those prior theories where they are correct but also predicts previously undocumented interactions between memory limitations and probabilistic expectations, which we confirmed in three behavioral experiments probing human processing of recursive structures.

Our results reveal that the well-documented difficulty of integrating long linguistic dependencies, which is at the heart of existing memory-based models (5, 7, 36), is substantially modulated by probabilistic expectations: The comparison between the One and Three conditions shows that such locality effects can be weakened or even reversed when the nonlocal syntactic structure has high a priori probability, a prediction that falls out naturally from our proposed unification of memory- and expectation-based perspectives. Our work further documents three prominent families of effects from the psycholinguistic literature in a single experiment and with a single model: locality effects (increased difficulty of Three), predictability effects (effect of embedding bias in the One condition), and semantic interference effects (effect of semantic compatibility). There has been considerable interest in a unified theoretical treatment of these families of effects; our work showcases how a single model can describe, in detail, how they interact. One group of phenomena not targeted by our experiments is similarity-based interference (43, 44). Investigating whether it can also be accounted for with this modeling framework is an interesting problem for future research.

Our resource-rational model is formally related to models in various domains. Classical work has shown that rational analysis of retention probabilities can account for fundamental properties of human memory (28, 29). Recent work (45–48) has formalized rational models of human working memory in some domains, such as visual working memory, using rate–distortion theory, an information-theoretic framework deriving high-fidelity encodings under resource constraints. The key difference between rate–distortion theory and our model is that the measure of economy is the fraction of available words here, while it is the number of encoded bits in rate–distortion theory. Applied to sentence comprehension, rate–distortion theory would lead to fully compressed “gist” representations of past context. Such fully compressed representations do not lead to the difficulty patterns observed in our experiments (see *SI Appendix*, section S8 for details). On the other hand, our model is also a simplification in that it models the recent context as a sequence of words, which may underestimate the role of memory representations of longer context where individual words may have been forgotten but memory of meaning remains. Further advances in machine learning may allow inferring a more sophisticated format of memory representations from resource-rational optimization.

In computer science, recursive structure is typically processed using stack-based data structures. Correspondingly, early models of human syntactic processing assumed bounds on the size of the stack, or the number of nodes that can be held in memory at the same time (2, 24). Such models predict that deeper embedding is more difficult, but do not predict that difficulty is modulated by statistical or semantic cues. Unlike stack-based architectures, our theory assigns a major role to probabilistic cues in establishing recursive structure. In this respect, it agrees with more-recent memory-based theories assuming that humans do not maintain data structures such as stacks, and, instead, establish syntactic structures using associative cue-based retrieval (5, 7, 49, 50). Models of associative retrieval as currently implemented (7) do not account for the distinctive difficulty patterns predicted by our model and observed in our experiments. Nonetheless, we view our theory as compatible with ideas from that literature. Our theory provides a computational-level model that makes predictions compatible with existing memory-based models, but—unlike those models—is rationally attuned to the rich statistical structure of language, enabling it to predict how memory limitations interact with probabilistic expectations. Our results suggest that identifying probabilistic versions of associative retrieval models, as algorithmic-level implementations of the resource-rational model described here, is an interesting problem for psycholinguistic research. See *SI Appendix*, section S7.2 for more on the implications of our results for retrieval-based memory models.

Our proposed unification of expectation-based and memory-based models rests on the idea that imperfect working memory representations are reconstructed rationally—although sometimes incorrectly—using knowledge of the statistics of the language. This idea has an important precedent in work on redintegration in verbal working memory (e.g., refs. 51–55), a process whereby degraded short-term memory is restored using knowledge from long-term memory. This has been applied to memory for word lists (e.g., ref. 52–55) and, more recently, memory for syntactic patterns (56). Our model provides an account of such processes grounded in Bayesian inference constrained by resource rationality. There are also models where working memory is treated not as a component of memory of its own but as emergent from the interaction of processing and long-term memory (57, 58). For such models, our results provide data on how long-term knowledge informs processing.

Our experiments capitalize on statistical correlates of syntactic structures in order to probe how probabilistic expectations interact with memory constraints. This has some parallels in prior work on expectation-based models that showed how correlations, such as between animacy and relative clause type, impact processing in ways not accounted for by existing memory-based accounts (e.g., refs. 59–61). Our work expands on this line of work by articulating an implemented theory of the interaction between memory constraints and probabilistic expectation.

Our model has a free parameter *δ*, the average number of retained words. We assumed a single value in deriving predictions and comparing to human reading times. Fitting it for individual subjects and understanding its relationship to established measures of individual differences is an interesting problem for future research.

Connectionist models of human syntactic processing (8, 62–64) aim to describe human processing using expectations derived from neural network representations, and have been proposed to model effects related to both memory limitations and probabilistic expectations. However, the differences between plain surprisal as computed by GPT-2 and resource-rational lossy-context surprisal show that human-like memory limitations need not emerge automatically in connectionist models.

We have shown how a model of resource-rational language processing can be scaled to the rich statistical structure of natural language. Our machine learning–based method may open the door to fitting sophisticated rational models on natural input statistics also in other domains of human cognition.

The generality of our model also suggests that similar phenomena might exist outside of language: Whenever humans process input that is too complex for all its parts to be attended to simultaneously, processing should be impacted by the statistical structure of similar inputs.

## Materials and Methods

### Nouns.

We collected nouns that can take a sentential complement, using the Penn Treebank (65), the English Web Treebank (66), the AnCoRA treebank (67) of Spanish, and the HDT Treebank (68) of German. We estimated embedding bias as the log-probability that “the NOUN” was followed by “that” using the English Wikipedia (2.3 billion words), the German Wikipedia (800 million words), and the Spanish Wikipedia (500 million words). See *SI Appendix*, section S11 for details. We validated the English estimates using two other large corpora of American and British English (*SI Appendix*, section S10.1).

### Model.

Resource-rational lossy-context surprisal is defined by a family of retention probabilities $\theta =\{q_{w,i}:i,w\}$, where *w* ranges over words and i=1,…,N, where *N* = 20 is the maximum context length considered, long enough to accommodate all contexts appearing in the experiments. We parameterize $q_{w,i}$ using a neural network that combines a past word’s identity and the number of intervening words, to output a retention probability (*SI Appendix*, section S1.1). The model *θ* gives rise to the likelihood p(c′|c) and thus the posterior p(c|c′). It is chosen to minimize average next-word surprisal for the resulting next-word posterior p(w|c′):

[2] $$\underset{\theta }{\min}E_{c^{*}w}E_{c\prime \sim p(c\prime |c^{*})}\left[-\log p(w|c\prime )\right],$$

where $c^{*}w$ are contexts in the corpus together with the next words, subject to the constraint that the average number of retained words does not exceed some bound $\delta \in R_{+}$,

[3] $$E_{c^{*}}E_{c\prime \sim p(c\prime |c^{*})}\left[\#\{i:c\prime_{i}\neq \mathrm{erased}\}\right]\leq \delta ,$$

where $c\prime =c\prime_{1}\ldots c\prime_{N}$ consists of the retained words and erased for the other words.

For each integer 0<δ<20, we solved [**2**] and [**3**] on large-scale text data from the English Wikipedia using machine learning methods based on neural networks (*SI Appendix*, section S1.3). We fitted the model on contexts $c^{*}$ of length *N* = 20 of continuous text, across sentence boundaries marked by periods. We removed commas from the text data, as they might provide confounding cues to hierarchical structure.

We show results at *δ* = 10 in Fig. 3; see *SI Appendix*, Fig. S4 for results at other values of *δ*.

We estimated the a priori statistics of English with GPT-2, and used importance sampling to compute model surprisal [**1**] (*SI Appendix*, section S1.4). Due to high computational resource demands, we used the medium-sized version of GPT-2 (345 million parameters). Larger versions of GPT-2 provide equivalent predictions in the zero-loss setting (*SI Appendix*, section S7.1).

### Experimental Setup for Reading Time Studies.

For all studies, the experimental protocol was approved by the Institutional Review Board at Stanford University. Informed consent was obtained from all participants. Each participant was presented with 10 critical trials. In both experiments, two trials were in One, and four trials were in Two and Three each. In experiment 2, half of the Two and Three trials were each in the Compatible (Incompatible) condition. We chose a small number of critical trials, to minimize any effect of statistical adaptation to center embeddings during the task. To maximize statistical precision, we selected 15 nouns with very high embedding bias and 15 nouns with very low embedding bias (*SI Appendix*, Fig. S36). For each participant, we sampled five nouns with high embedding bias and five nouns with a low value, and matched these with the 10 critical trials. For each participant, we also sampled 30 fillers from a pool of 56 fillers from a prior reading time study of center embeddings (42). To remove semantic anomalies due to presupposition violations (e.g., “the fact was wrong”), we classified the nouns into entailing (e.g., “fact”), nonentailing neutral (e.g., “claim”), and nonentailing negative (e.g., “accusation”) nouns, and classified items for compatibility with each of these three classes (*SI Appendix*, section S11). For each participant, we matched the 10 nouns with semantically compatible items.

For the maze task, we generated distractors automatically (39) using the Gulordava language model (69): these distractors have extremely low contextual probability, while being matched with the target word in frequency and length. Distractors were matched across conditions, except within the second-to-last verb phrase in the (In)Compatible conditions in experiment 2. In particular, distractors were matched on the critical word across all conditions.

When participants made a mistake (i.e., chose the distractor), they were prompted to retry the current word (70). Reaction times on such trials were excluded; this choice did not impact conclusions (*SI Appendix*, section S3.6).

For each subject, trials were presented in random order so that no two critical trials were adjacent. Participants, recruited on the Prolific academic platform, took a median of 13 min, and received £2.20 (≈3 USD).

### Data Analysis for Reading Times.

We excluded trials 1) with an incorrect answer, 2) from participants who made errors on more than 20% of words, and 3) below or above 99% of all reading times. See *SI Appendix*, section S3.6 for robustness to condition 1, and see *SI Appendix*, section S3.7 for robustness to condition 3. We then analyzed log-transformed reading times on the final verb using Bayesian mixed-effects models implemented in Stan (71) using brms (72). See *SI Appendix*, section S3.3 for priors and robustness to prior choices. We used contrast coding with the presence of a “that”-clause (One vs. Two/Three), depth (Two vs. Three), and the compatibility manipulation (Compatible vs. Incompatible) as contrasts. Embedding bias was centered, and all nonvacuous binary interactions were added as fixed effects (*SI Appendix*, section S3.2). We included the maximal random effects structure justified by the experimental design, entering items, nouns, and participants as random effects. In order to estimate effects in raw reading times (milliseconds), we first computed the predicted log-transformed reading time in both conditions (e.g., Compatible and Incompatible), then transformed both into milliseconds by exponentiating, and computed the difference (see *SI Appendix*, section S3.4 for further details). In Fig. 3, we plot the posterior mean of the predicted reading time in all conditions for nouns with embedding bias matching “fact” or “report.” Error bars represent the posterior SD.

### Details for Production Study.

We constructed 28 items of the form “The XXX that the diplomat who the senator,” and selected 12 nouns, 6 each with very high or very low embedding bias. For each participant, we randomly paired items and nouns. The 12 critical trials were presented in random order with 27 fillers. A linguist manually annotated, for each completion provided, whether the correct number of verb phrases (three) was produced. The annotator was blind to the identity of the noun.

In Spanish and German, we selected 20 nouns with very high or very low embedding bias in each language, sampling 6 high and 6 low embedding bias nouns for each participant. As in the English version, we randomly matched 12 items with the 12 sampled nouns for each participant. Fillers were translated from the English experiment.

In German, we further constructed 12 matrix sentences (e.g., “Klaus said that”), and randomly matched them with items and nouns for each participant.

We conducted a Bayesian trial-by-trial logistic mixed-effects analysis with embedding bias as a fixed effect, and random effects of nouns, items, participants, and (in German) matrix sentences. See *SI Appendix*, section S4 for details.

## Supplementary Material

Supplementary File

## Data Availability

Fitted retention probabilities and model predictions have been deposited in Zenodo (https://zenodo.org/record/6602698) (73), (https://zenodo.org/record/6988696) (74). Anonymized reading times, language production data, and source code have been deposited in GitLab (https://gitlab.com/m-hahn/resource-rational-surprisal) (75).

## Footnotes

^*^E. Gibson, E. Fedorenko, “The domain-generality of working memory resources for language” in *Architectures and Mechanisms for Language Processing (AMLaP)* (2011).
