# Supplementary material for: A resource-rational model of human processing of recursive linguistic structure
Source: Proc Natl Acad Sci U S A. 2022 Oct 19;119(43):e2122602119. doi: 10.1073/pnas.2122602119 (PMC9618130; doi:10.1073/pnas.2122602119)
Supplement: Supplementary File [file pnas.2122602119.sapp.pdf]

Supplementary Information: A Resource-Rational Model of Human Processing of Recursive Linguistic Structure

Michael Hahn, Richard Futrell, Roger Levy, Edward Gibson

mhahn2@stanford.edu

October 7, 2022

Contents

I Details for Model and Experiments **3**

1 Implementation Details **3**
1.1 Formal Description and Parameterization of Model 3
1.2 Objective Function 4
1.3 Optimization Method 5
1.4 Posterior Inference with Importance Sampling 6
1.5 Model Runs 7

2 Detailed Model Results **8**
2.1 Differences between TWO and THREE 9
2.2 Uniform Loss Model 12

3 Additional Analyses for Reading Times in Experiments 1–2 **13**
3.1 Assignment of Trials to Conditions 13
3.2 Coding of Conditions 13
3.3 Details for Data Analysis and Mixed-Effects Regression 13
3.4 Effects in Raw Reading Times 14
3.5 Results for Experiments 1 and 2 14
3.6 Analysis of Incorrect Responses 17
3.7 Effect of Outlier Removal 17

4 Additional Analyses for Experiment 3 **22**

II Further Experiments and Meta-Analysis **23**

5 Ratings Studies **23**
5.1 Ratings Study S1 23
5.2 Ratings Study S2 24

6 Previous Reading Time Experiments **26**
6.1 Experiment S1: Reading Times in THREE . . . 26
6.2 Experiment S2: Reading Times in THREE+COMPATIBLE . . . 27
6.3 Experiment S3: Reading Times in ONE and TWO . . . 27
6.4 Experiment S4: Effect of Compatibility on Reading Times . . . 28
6.5 Experiment S5: Large-Scale Replication of Experiments S3 and S4 . . . 28
6.6 Meta-Analysis for Reading Time Studies . . . 29

III Comparison with Other Models and Theories **33**

7 Comparison with other Theories **33**
7.1 Conceptual predictions for DLT and Surprisal Theory . . . 33
7.2 Implications for Cue-Based Retrieval Accounts of Center Embedding Difficulty . . . 34
7.3 Other Objective Functions . . . 36

8 Comparison with Information-Theoretic Bottleneck **38**

IV Further Analyses **41**

9 Model Fit on Fillers **41**

10 Comparing Embedding Bias with other Possible Predictors **43**
10.1 Estimating Embedding Bias Using Other Corpora . . . 43
10.2 Embedding Bias and other Predictors . . . 43

V Materials **48**

11 Nouns and Embedding Bias **48**

12 Stimuli (Reading Time Studies) **52**
12.1 Stimuli (Experiment 1) . . . 52
12.2 Stimuli (Experiment 2) . . . 54

13 Stimuli (Production Study) **57**
13.1 English . . . 57
13.2 German . . . 58
13.3 Spanish . . . 59

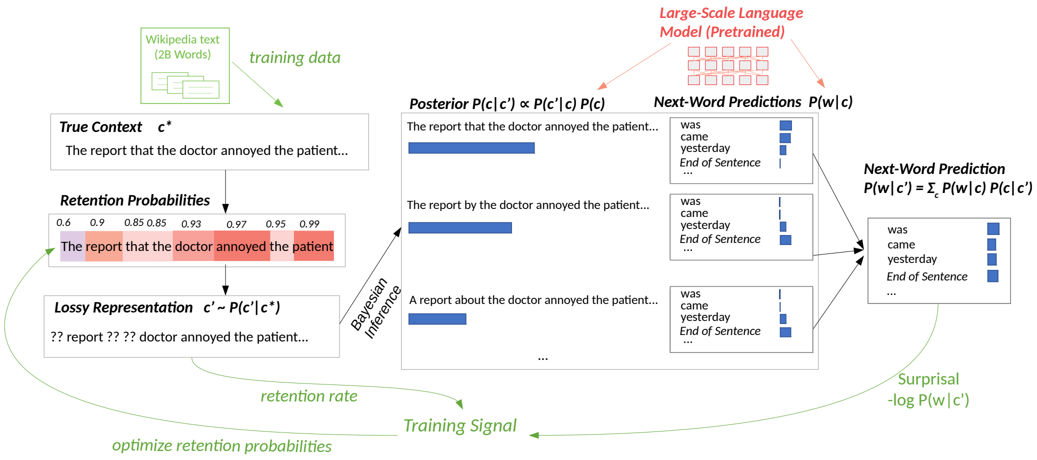

Figure 1: Implementation, including fitting procedure (green) and inference (red). Compare Figure 2 in the main paper. The model is fitted on text from the English Wikipedia, unrelated to the center embedding stimuli of interest. The training signal consists of the model surprisal incurred on the next word, and a constraint on the number of retained words; the retention probabilities are optimized to minimize surprisal under an upper bound on the average number of retained words. When computing model predictions (red), both the prior $P(c)$ and the next-word probabilities $P(w|c)$ are computed using a large-scale pretrained language model, GPT-2.

Part I
Details for Model and Experiments

1 Implementation Details

Here, we describe how we fitted the model for the resource-rational objective function and how we computed next-word predictions $p(w|c')$. Our fitting method is based on successful recent approaches to formally related models in various domains of machine learning [42, 31, 49, 56, 66, 33, 40]. These are general techniques that may also be applicable to other rational and resource-rational models in cognition.

Our Python code for optimizing retention probabilities and calculating model surprisal is available at `https://gitlab.com/m-hahn/resource-rational-surprisal/-/tree/main/model`.

1.1 Formal Description and Parameterization of Model

Before describing the method used for fitting the model, we revisit the definition of the model formally, and describe how it is parameterized in our implementation.

Representation of Context The model operates on contexts $c = c_1 \dots c_N$. For the purpose of computational implementation, we set a bound $N = 20$ on the maximum size of the context, long enough to accommodate all prefixes in the stimuli. The model is fitted on continuous text, across sentence boundaries.

In line with standard practice in natural language processing, punctuation symbols (e.g., ".") are treated as individual tokens. The model is constrained to never forget periods or other kinds of punctuation, which helps maintain information about sentence boundaries. However, we remove commas from the text data as they might provide confounding cues to hierarchical structure.

For implementation reasons, neural network models have a bounded vocabulary of the most common words, and low frequency words are represented by an out-of-vocabulary (OOV) token. We selected the 50,000 most common words in the training set for the vocabulary. We constrained the model to never erase the OOV token, in order to prevent situations where the posterior assigns high probability to inputs where erased words have been reconstructed as OOV – such inputs may not have a well-defined syntactic structure, which could make next-word surprisals meaningless.

Likelihood The model is defined by a family of retention probabilities $\theta = \{q_{w,i} : i, w\}$ where w ranges over words and $i = 1, \dots, N$. Then $c' = c'_1 \dots c'_N$ is given by determining every c'_i independently as

$$c'_i = \begin{cases} c_i & \text{with probability } q_{c_i, N-i} \\ \text{ERASED} & \text{otherwise} \end{cases} \tag{1}$$

Thus, given an input $c = c_1 \dots c_N$, θ gives rise to a likelihood $p_\theta(c'|c)$.

Parameterizing the Model $p_\theta(c'|c)$ The key to parameterizing the model is to parameterize the retention probabilities $q_{w,d}$. For this, we draw on standard machine learning techniques from Natural Language Processing. Word identity is encoded through word embeddings [27], i.e., assigning a vector to each word in the vocabulary. Relative positions are encoded though positional embeddings, i.e., assigning a vector to each of the integers $i = 1, \dots, N$ [62]. The machine learning literature has identified several effective methods for combining pairs of embeddings into scalars. We found that a deep bi-affine model [18] yielded the best value for the resource-rational objective function. We made this choice purely on the basis of the achieved objective function, without considering model predictions on recursive structure. The deep bi-affine model is given by

$$q_{w,d} := \sigma\left(vp_i + w \cdot \text{ReLU}(Ax_i) + p_i^T \cdot B \cdot \text{ReLU}(Cx_i)\right) \tag{2}$$

where $x_i \in \mathbb{R}^{d_{word}}$ is the word embedding, $p_i \in \mathbb{R}^{d_{pos}}$ the positional embedding, $v \in \mathbb{R}^{d_{pos}}$, $w \in \mathbb{R}^{d_{hidden}}$ are parameter vectors, and $A \in \mathbb{R}^{d_{hidden} \times d_{word}}$, $B \in \mathbb{R}^{d_{pos} \times d_{hidden}}$, $C \in \mathbb{R}^{d_{hidden} \times d_{word}}$ are parameter matrices. σ is the sigmoid or inverse-logit function. We chose the dimensionalities to be $d_{word} = 1024$, $d_{pos} = 256$, $d_{hidden} = 500$ to optimize the objective function. We initialize the word embeddings from those of the pretrained inference network $q_\theta(w|c')$ (see below, Section 1.3). B is initialized at zero [18]. The other parameters are initialized randomly using the defaults of the PyTorch library [53].

1.2 Objective Function

Recall the objective function for the retention probabilities $\theta = \{q_{w,i} : i, w\}$:

$$\min_\theta \mathbb{E}_{c^*w} \mathbb{E}_{c' \sim p_\theta(c'|c^*)} \left[-\log p(w|c')\right] \tag{3}$$

where c^*w are contexts in the corpus together with the next words, subject to the constraint that an average of at most δ words are retained on average across contexts:

$$\mathbb{E}_{c^*} \mathbb{E}_{c' \sim p(c'|c^*)} \left[\#\{i : c'_i \neq \text{ERASED}\}\right] \leq \delta \in \mathbb{R}_+. \tag{4}$$

Here, the retention rate δ is a parameter, varied from 1 to 19 in the model simulations (see Figure 4) This problem can be solved efficiently by combining neural variational inference [50, 39] with Lagrangian duality [8]. Neural variational inference combines the problems of optimizing θ and estimating $p(w|c')$ (which depends on the posterior $p(c|c')$, and thus ultimately on θ) into a single optimization problem, using auxiliary neural networks that provide learned representations of the posterior and that are optimized simultaneously with θ. Thus, we expresses $p(w|c')$ using a powerful function approximator $q_\psi(w|c')$, with

parameters ψ chosen to minimize the KL-Divergence between the approximator and the true predictive distribution. Such a network is referred to as an *inference network* [50, 39]. Using this method, we rewrite (3) as

$$\min_{\theta,\psi} \mathbb{E}_{c^* w} \mathbb{E}_{c' \sim p_\theta(c'|c^*)} \left[-\log q_\psi(w|c') \right] \tag{5}$$

Using Gibbs' Inequality [12], one can prove that this problem is equivalent to (3), provided that the family of approximators is sufficiently expressive (such as a family of neural networks). Finally, to efficiently take the constraint (4) into account, we draw on Lagrangian duality.[1] We thus obtain the following optimization problem:

$$\min_{\kappa \leq 0} \max_{\theta,\psi} \mathcal{L}_1(\theta, \psi, \kappa) \tag{6}$$

where

$$\mathcal{L}_1(\theta, \psi, \kappa) := \mathop{\mathbb{E}}_{c} \mathop{\mathbb{E}}_{c' \sim p_\theta(c'|c)} \left[\log q_\psi(w|c') + \kappa \cdot \left[\#\{i : c'_i \neq \text{ERASED}\} \right] - \delta \right] \tag{7}$$

This objective function is amenable to standard optimization methods combining neural network parameterizations of $q_\psi(w|c')$ with gradient descent, as we describe in the following sections.

We use the same procedure to simultaneously also create a second inference network $q_\varphi(c|c')$ for the posterior $p(c|c')$, which we use as a proposal distribution when estimating surprisal (see Section 1.4). To achieve this, we maximize

$$\mathcal{L}_2(\varphi) := \mathop{\mathbb{E}}_{c} \mathop{\mathbb{E}}_{c' \sim p_\theta(c'|c)} \log q_\varphi(c|c') \tag{8}$$

This minimizes the KL divergence between $q_\varphi(c|c')$ and the true posterior $p(c|c')$, averaged over contexts c and representations c'.

Parameterizing the Inference Networks As described above, the model has two inference networks, one ($q_\psi(c|c')$) modeling recovery of the true context c from c', and one ($q_\psi(w|c')$) modeling prediction of the nect word w from c'. The parameterization for the inference networks follows standard sequence modeling techniques from Natural Language Processing; the precise numerical parameters were identified to optimize the numerical value of (6) without considering model predictions on recursive structure. We parameterize the prediction model $q_\psi(w|c')$ as a standard LSTM language model [35] with 1024 hidden units and 2 layers. We parameterize the reconstruction model $q_\psi(c|c')$ using a standard sequence-to-sequence LSTM network [59, 45], which transduces the input $c' = y_1 \dots y_N$ into a probability distribution over output sequences $c = x_1 \dots x_N$.

1.3 Optimization Method

Here, we describe how we solve the variational formulation (6) of the resource-rational objective function. All choices were made to optimize the numerical value of (6), without yet considering model behavior on the sentences considered in this study.

We solve (6) using stochastic gradient descent (SGD) combining ordinary backpropagation with the REINFORCE estimator [65], a scheme commonly used for problems similar to ours [42, 31, 49, 56, 66, 33, 40]. This leads to the following expressions for the gradients (the first line uses the REINFORCE or

[1]We established the validity of the Lagrangian dual objective as follows. Note that, a priori, for nonconvex problems such as the one we are considering, the mini-max problem (6) need not be equivalent to the original problem, as the solution (θ, ψ) of the mini-max problem need not satisfy the constraint (4). However, we empirically observe that the parameters θ obtained by solving (6) do satisfy the constraint. In this case, the optimal solution for (6) has $\kappa = 0$. We then note $\mathcal{L}_1(\theta, \psi, 0) = (3)$, establishing the equivalence of the dual problem with the original problem.

Component	Parameter	Explored Range
Amortized Posterior	Learning Rate	0.001, 0.01, 0.1, 0.2
Memory Model	Learning Rate	0.00002, 0.00005, 0.0001
	Momentum	0.5, 0.7, 0.9
Dual κ	Learning Rate	0.01, 0.02, 0.05, 0.1, 0.2, 0.3

Table 1: Hyperparameters for solving the resource-rational objective function. These were selected to optimize (6), without considering model behavior on recursive structure.

policy-gradient theorem, [65]):

$$\partial_\theta \mathcal{L}_1(\theta, \psi, \kappa) = \mathop{\mathbb{E}}_{c} \mathop{\mathbb{E}}_{c' \sim p_\theta(c'|c)} \left[\big(\partial_\theta \log p_\theta(c'|c)\big) \cdot \log q_\psi(w|c') + \kappa \sum_{i=1}^{N} \partial_\theta q_{c_i, N-i} \right] \tag{9}$$

$$\partial_\kappa \mathcal{L}_1(\theta, \psi, \kappa) = \mathop{\mathbb{E}}_{c} \mathop{\mathbb{E}}_{c' \sim p_\theta(c'|c)} \big[[\#\{i : c'_i = \text{ERASED}\}] - \delta \big] \tag{10}$$

$$\partial_\psi \mathcal{L}_1(\theta, \psi, \kappa) = \mathop{\mathbb{E}}_{c} \mathop{\mathbb{E}}_{c' \sim p_\theta(c'|c)} \partial_\psi \log q_\psi(w|c') \tag{11}$$

$$\partial_\phi \mathcal{L}_2(\theta, \psi, \kappa) = \mathop{\mathbb{E}}_{c} \mathop{\mathbb{E}}_{c' \sim p_\theta(c'|c)} \partial_\phi \log q_\phi(c|c') \tag{12}$$

In each step, the SGD algorithm takes a sample $c = x_1 \dots x_N x_{N+1}$ from the dataset and samples $c' \sim p_\theta(c'|c)$, and then updates the parameters using the expressions inside the expectations (9–12) as (unbiased) estimates of the gradient. In each update step, κ is updated in direction opposite from ψ, θ, and κ was clipped to be nonpositive.

We minibatch 12 copies of each training example, independently taking one sample from $p_\theta(c'|c)$ for each copy, but did not batch different examples together. We found this to improve optimization much more than other common choices, such as taking a larger batch size with only one copy per example. We further add a control variate [65] that reduces variance without introducing bias.

We run SGD for 200K steps. Empirically, we observe that the retention probabilities essentially converge, and that the objective function shows little improvement beyond this.

Learning rates and momentum are shown in Table 1. We found similar values of the objective function across a wide range of hyperparameters, and thus sampled different possible parameters for each model run to maximize coverage of possible hyperparameters.

We pretrain the inference networks on samples from a uniform loss model, where each word is retrieved with 80% probability. To eliminate potential artifacts of individual model runs, we constructed six reconstruction posterior models q_φ and randomly chose one of each every time we constructed an optimized model θ. These networks were trained on the full corpus (2.3 Billion words) with early stopping until convergence. This pretraining approach greatly reduces computation time and speeds up model convergence. We continue training the reconstruction network $q_\varphi(c|c')$ together with θ; this is particularly important because q_φ also serves as a proposal distribution in surprisal computation (see Section 1.4) and thus needs to be well-adapted to the behavior of θ. However, we found that continuing training the prediction network $q_\psi(w|c')$ was computationally costly without strong improvement of the objective function; we thus fixed it in part of the model runs to reduce computing cost, without observing differences in the results.

1.4 Posterior Inference with Importance Sampling

We now describe how, given optimized retention probabilities θ, we used importance sampling and GPT-2 to calculate model surprisal. By the rules of probability, the surprisal of a word w given a memory

representation c' is given as:

$$-\log p(w|c') = -\log \sum_{\tilde{c}} P(w|\tilde{c}) p(\tilde{c}|c') \tag{13}$$

where the posterior is given by

$$p(\tilde{c}|c') = \frac{p(c'|\tilde{c})P(\tilde{c})}{\sum_{\tilde{c}} p(c'|\tilde{c})P(\tilde{c})} \tag{14}$$

Direct calculation from this expression is intractable due to the sum over all sequences in the denominator.[2]

A standard approach for estimating such quantities is importance sampling [32], which obtains samples from a *proposal distribution* from which samples can be taken easily, and reweights these to match the desired target distribution. We use the inference network $q_\phi(c|c')$ as a proposal distribution and GPT-2-Medium for the prior $P(\tilde{c})$. This leads to

$$p(w|c') \approx \frac{\sum_{i=1}^{K} w_i P(w|c^{(i)})}{\sum_{i=1}^{K} w_i} \tag{15}$$

where $c^{(i)}$ are sampled from $p_\psi(c^{(i)}|c')$ and the importance weights are given as

$$w_i := \frac{P(c^{(i)})p(c'|c^{(i)})}{p_\psi(c^{(i)}|c')} \tag{16}$$

In our experiments, we set $K = 24$ to balance computational cost with stability of estimates.

As described in the main paper, we predict human reading times from the expected model surprisal averaged over memory representations, $\mathbb{E}_{c' \sim p(c'|c)} [-\log P(w|c')]$. As the set of all possible representations is exponentially large, we computed a Monte Carlo estimate using 6 samples $c' \sim p_\theta(c'|c)$. Thus, in total, model predictions are computed using $6 \cdot 24 = 144$ possible contexts $\tilde{c}$ for each trial.[3]

1.5 Model Runs

We solved the resource-rational objective function for $\delta = 1, 2, \ldots, 19$. The stochastic gradient descent optimization method described in Section 1.3 is nondeterministic, leading to different approximate optimizers of the objective function on every run. We created at least 4 runs for every parameter setting, and at least 10 for each setting in the range used for model predictions ($4 \leq \delta \leq 15$). Model runs were run by a script that was run in parallel on multiple machines, randomly choosing configurations for which the intended number of runs had not been reached; the overall number of model runs per configuration was thus determined by compute availability and chance. A total of 279 model runs resulted.

[2] An alternative approach could be to directly use the inference network $q_\psi(w|c')$ as a stand-in for $p(w|c')$. However, importance sampling allows us to draw on the modeling power of GPT-2, trained on an even larger dataset than our inference network.

[3] As our model simulations use 58 nouns paired with (in Experiment 2) 42 items and 5 conditions, it was not feasible to conduct all simulations with a larger number of samples. However, we verified that larger sample sizes led to equivalent results when subsampling a smaller set of nouns and items.

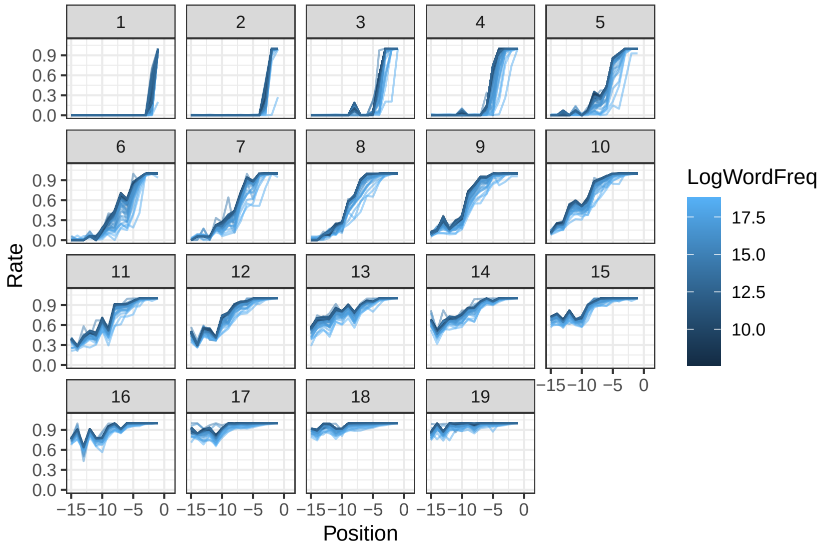

Figure 2: Retention probabilities, as a function of the average number of retained words ("retention rate", $1 \leq \delta \leq 19$), number of intervening words (x-axis), and log word frequency (color), averaged across all model runs at a given retention rate δ. For visibility, we focus on distances ≤ 15. We randomly chose a span of 200 words from the Wikipedia data, held out in the training process. Here, we plot retention probabilities for each word in the span, colored by log word frequency in the Wikipedia corpus. There is a strong recency effect, whereby recent words are more likely to be retained. There furthermore is a frequency effect, whereby lower-frequency words (dark blue) are more likely to be retained. Note that the fitted retention probabilities are not fully monotonic as a function of the distance; this can be attributed to the fact that the model has no built-in bias towards progressive decay of representations; this is learnt entirely from the resource-rational objective as applied to the Wikipedia corpus.

2 Detailed Model Results

Here, we report detailed results from the models.

Retention Probabilities In Figure 2, we plot retention probabilities as a function of word frequency. There is a recency bias, whereby more recent words have higher retention probabilities. There is also a word frequency effect, such that low-frequency words are more likely to be retained.

We further specifically plot the retention probabilities for the words most pertinent to the phenomena studied in our experiments: "that" and prepositions, in Figure 3; see caption for details.

Model Surprisal In Figure 4, we show model surprisal on the critical word in the stimuli in Experiments 1–2 across all model parameters (see caption for details).

Model Fit to Reading Times In Figure 5, we show model fit to log reading times on the critical words from trial-by-trial mixed-effects models with model surprisal as fixed effect, and random intercepts for nouns, items, and participants. Model surprisal was averaged across all model runs for each δ. These models were fitted with lme4 [6] with maximum likelihood. [4]

[4] A possible concern with these analyses is that the number of model runs differs between parameter settings, so that surprisal estimates might have lower variance when there are more model runs. To rule out this concern, we also conducted an analysis with AICs computed for each model run, and then averaged, and found equivalent results.

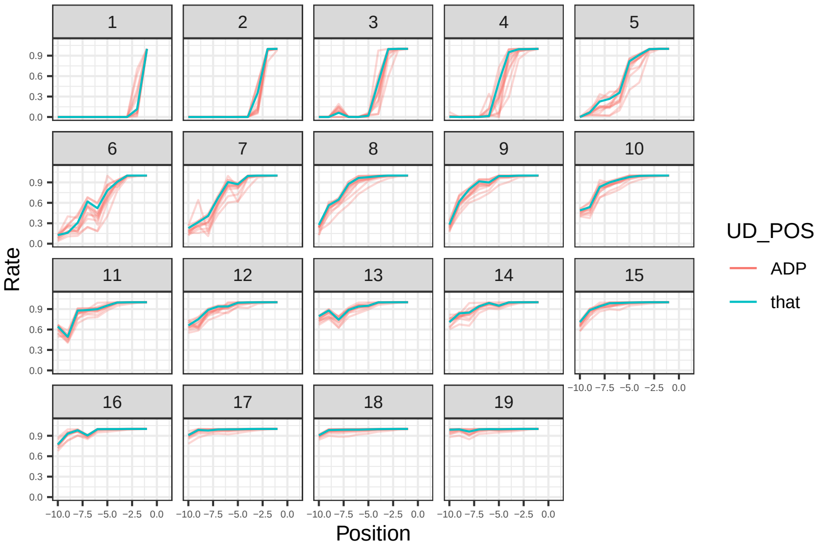

Figure 3: Retention probabilities for "that" (blue) and for prepositions (red) found in the span of text used for Figure 2. For visibility, we focus on distances ≤ 10. While both groups of words show very similar trajectories, prepositions have mostly lower retention probabilities than "that". That is, "that" is a priori less likely to be forgotten than most prepositions are. This means that, when "that" has been forgotten, then Bayes's rule (Equation 14) entails that – all things being equal – a higher posterior probability will be assigned to alternative contexts with a preposition (e.g., "of", "by") in place of "that". This asymmetry increases the bias towards such structurally different alternatives, unless those have low a-priori probability.

2.1 Differences between Two and Three

Figure 4 shows that varying the average number of retained words (δ, "retention rate") changes the relative strength of effects in Two and Three. This happens because of floor or ceiling effects when the true structure is essentially always or never reconstructed: For higher retention rates ($\delta \gtrapprox 10$), effects of compatibility and embedding bias are stronger in Three than in Two; this happens because the Two condition is unaffected by memory loss at high retention rates. In contrast, for low retention rates, effects of compatibility and embedding bias are stronger in Two, because the syntactic structure is rarely reconstructed correctly in the Three condition. Human reading times show stronger effects in Three than in Two, as shown by interactions Depth:EmbeddingBias and Depth:Compatibility in a meta-analysis across reading times (Figure 23 in Section 6.6), suggesting that effects of memory limitations are relatively mild in the Two condition. This agrees with model behavior at higher average retention rates (e.g., $\delta \approx 10, \dots, 13$ in Figure 4). However, the fact that effects of embedding bias and compatibility can be shown individually in human reading times even within the Two condition ("FactReportDifferenceTwo" and "CompatibilityEffect_Two" in Figure 23) shows that this is not a categorical difference: Similar to the behavior of the model, humans are impacted by memory limitations even in the simple Two condition, albeit to a lesser degree than in the more difficult Three condition. A prediction of the model is thus that, in situations where comprehenders are more forgetful or less attentive, effects should increase within Two and decrease within Three.[5]

[5] Cf Nicenboim et al. [51] for conceptually related experimental findings, where locality effects increased for high-capacity readers in an experiment in Spanish.

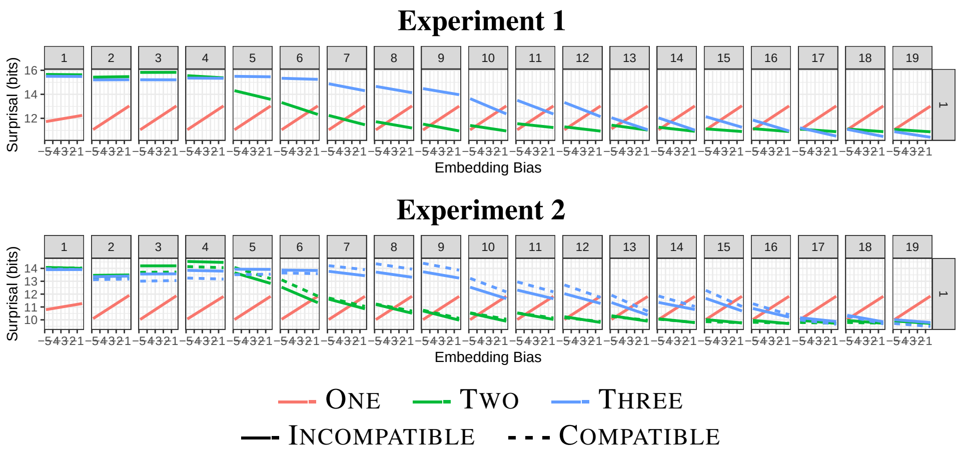

Figure 4: Model surprisal on the final verb across values of the average number of retained words δ ("retention rate"). We show linear fits across all item-noun-pairs, averaged across all model runs at a given δ.

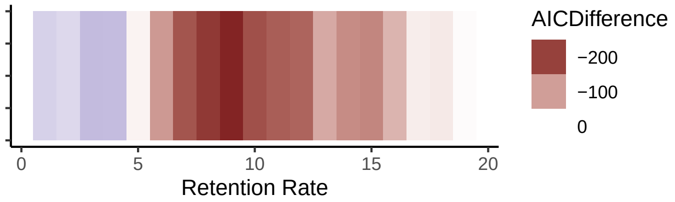

Figure 5: Model fit to critical log RTs measured by AIC as a function of the retention rate δ, for the data from Experiments 1 and 2. Compare Figure 4. Model fit is computed using frequentist mixed-effects models with model surprisal as fixed effects predictor, and random intercepts for nouns, items, and participants (see text for details). Model fit is compared to the AIC achieved by plain GPT-2 surprisal; negative values indicate a better model fit than GPT-2. Fit substantially improves over GPT-2 throughout the range where the three predictions described in the main paper are borne out (compare Figure 4).

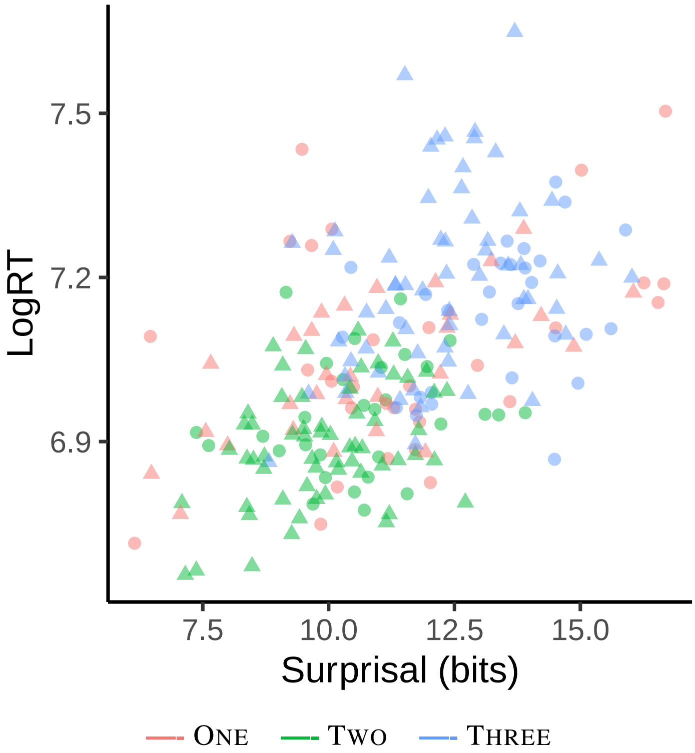

Figure 6: Scaling between surprisal and reading times, at $\delta = 10$, for Experiment 1 (circles) and Experiment 2 (triangles). Colors indicate conditions as in the main paper. For each experiment, we plot the mean surprisal and reading time across all trials with a given combination of noun and condition (One/Two/Three crossed with compatibility). Scaling of reading times with surprisal is similar in both experiments: We conducted Bayesian mixed-effects models predicting log-transformed reading times from model surprisal with a main effect and interaction including the contrast between the two experiments, with full random intercepts and slopes for nouns, items, and participants. We found a consistent main effect of surprisal ($\beta = 0.066$, 95% CrI $[0.06, 0.07]$), but no evidence for either a main effect of experiment ($\beta = 0.02$, 95% CrI $[-0.05, 0.1]$) nor for an interaction between surprisal and experiment ($\beta = 0.01$, 95% CrI $[-0.01, 0.03]$). We note that Figure 3 in the main paper might seem to exhibit a difference in the scaling of model surprisal and reading times in the two experiments; this analysis shows that this difference is not statistically meaningful.

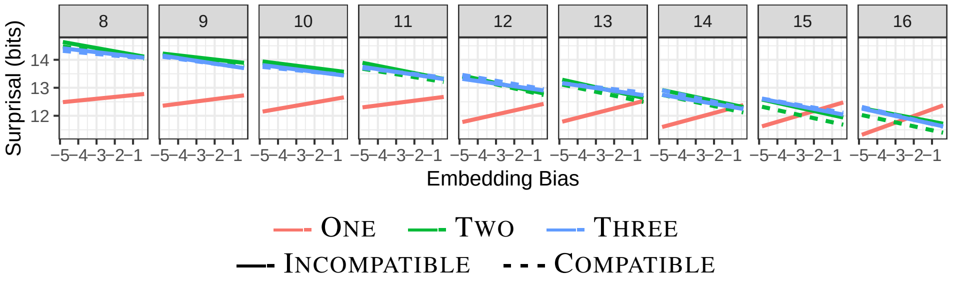

Figure 7: Model surprisal under uniform loss, for different values of δ. The effect of embedding bias is predicted across various values of δ. However, the effects of semantic compatibility and the TWO-THREE contrast are predicted inconsistently, either with no significant effect or sometimes in the opposite direction compared to the resource-rational model and the human data.

2.2 Uniform Loss Model

In Figure 7, we show model surprisal under the assumption of uniform loss, as in the simulations of Futrell et al. [22]. That is, given an average number of retained words, $\delta \in [0, 20]$, each word was retained with a uniform probability of $\frac{\delta}{20}$. The effect of embedding bias is predicted across various values of δ, confirming substantial robustness of this novel prediction. However, the effects of Compatibility and the TWO-THREE contrast are predicted inconsistently, sometimes even in the opposite direction from the resource-rational model. This shows that retention probabilities optimized for the resource-rational objective function match human reading times considerably better than uniform retention probabilities would.

We also considered a window-based model where memory representations consist of the past $K = \delta N$ words. Such a model can account for the effect of depth, but it cannot account for the effect of embedding bias: If the identity of the noun is to impact difficulty, then K must be large enough to include the noun, but then will also include *that*. Therefore, the effect of embedding bias in the presence of a *that*-clause would not arise in such a model.

3 Additional Analyses for Reading Times in Experiments 1–2

3.1 Assignment of Trials to Conditions

Here, we summarise the assignment of trials to conditions in Experiments 1 and 2, as described in the main paper:

# of trials	Experiment 1	Experiment 2
2	ONE	ONE
2	TWO+INCOMPATIBLE	TWO+INCOMPATIBLE
2	TWO+INCOMPATIBLE	TWO+COMPATIBLE
2	THREE+INCOMPATIBLE	THREE+INCOMPATIBLE
2	THREE+INCOMPATIBLE	THREE+COMPATIBLE
30	Fillers	Fillers

3.2 Coding of Conditions

In the mixed-effects analyses, we used Contrast Coding to code conditions using three predictors: One for the contrast between the ONE condition and the TWO/THREE conditions (EMBEDDING), one for the contrast between the TWO and the THREE condition (DEPTH), and one for the contrast between COMPATIBLE and INCOMPATIBLE (COMPATIBLE). All predictors were centered. This leads to the following coding:

Condition	EMBEDDING	DEPTH	COMPATIBLE
ONE	-0.9	0	0
TWO+INCOMPATIBLE	+0.1	-0.5	-0.5
TWO+COMPATIBLE	+0.1	-0.5	+0.5
THREE+INCOMPATIBLE	+0.1	+0.5	-0.5
THREE+COMPATIBLE	+0.1	+0.5	+0.5

In Experiment 2, the fixed effects structure contained these fixed effects, Embedding Bias, and all non-degenerate[6] binary interactions. In Experiment 1, main effects and interactions including compatibility were omitted.

Further, there were random effects for item, subjects, and nouns. The random effects structure was the maximal structure justified by the experimental design [5]: Items and participants had random slopes for all fixed effects. Nouns had random slopes for all fixed effects not involving Embedding Bias (Embedding Bias is constant within each noun, so that per-noun slopes are not justified for it).

3.3 Details for Data Analysis and Mixed-Effects Regression

We implemented models in Stan [10] using `brms` [9]. Embedding Bias was centered. Following Bürkner [9], the following prior was chosen. Fixed effect coefficients had flat priors. The intercept had a Student's t prior with 3 degrees of freedom, scale 2.5 and location 7, where the location parameter was chosen based on the mean log reading time in preceding studies (Section 6). Covariance matrices for random effects were parameterized as the combination of a correlation matrix and a vector of standard deviations [4]. Correlation matrices had $LKJ(1)$ priors [43]. The standard deviations for random effects and for the residuals had Student's t priors with 3 degrees of freedom, scale 2.5, and location 0. See the meta-analysis in Section 6.6 with Figure 24 for an analysis with a more strongly regularizing prior.

Posterior inference used the NUTS sampler [36] implemented in Stan. We ran four chains with 8000 iterations each, of which the first half were discarded as warmup samples. Convergence was assessed using $\widehat{R}$ and visual inspection of chains.

[6] I.e., the interactions EMBEDDING:DEPTH and EMBEDDING:COMPATIBLE are not meaningful and omitted.

3.4 Effects in Raw Reading Times

Here, we detail how we derived posteriors for effects in raw reading times from the Bayesian mixed-effects model.

We compute the effect of Depth in raw reading times:

1. Reading time in the TWO condition: $R_{\text{TWO}} = \exp(\alpha + 0.1 \cdot \beta_{\text{EMBEDDING}} - 0.5 \cdot \beta_{\text{DEPTH}})$
2. Reading time in the THREE condition: $R_{\text{THREE}} = \exp(\alpha + 0.1 \cdot \beta_{\text{EMBEDDING}} + 0.5 \cdot \beta_{\text{DEPTH}})$

Then the effect of Depth is given as:

$$\widehat{\Delta}_{Depth} = R_{\text{THREE}} - R_{\text{TWO}} \tag{17}$$

Next, the effect of compatibility is given as

$$\widehat{\Delta}_{\text{COMPATIBILITY}} = R_{\text{COMPATIBLE}} - R_{\text{INCOMPATIBLE}} \tag{18}$$

where

1. Reading time in the COMPATIBLE conditions: $R_{\text{COMPATIBLE}} = \exp(\alpha + 0.1 \cdot \beta_{\text{EMBEDDING}} + 0.5 \cdot \beta_{\text{COMPATIBLE}})$
2. Reading time in the INCOMPATIBLE conditions: $R_{\text{INCOMPATIBLE}} = \exp(\alpha + 0.1 \cdot \beta_{\text{EMBEDDING}} - 0.5 \cdot \beta_{\text{COMPATIBLE}})$

For Figure 3 in the main paper, the reading time for "*fact*" (similarly "*report*") in ONE (similarly for the other conditions) is given as[7]

$$\exp(\alpha - 0.1\beta_{\text{EMBEDDING}} + \beta_{\text{EMBEDDINGBIAS}} E_{fact} - 0.1\beta_{\text{EMBEDDING:EMBEDDINGBIAS}} E_{fact}) \tag{19}$$

where E_{fact} denotes the centered embedding bias of "fact". We obtained posteriors for these effect sizes by transforming the posterior samples for the coefficients. Figure 3 in the main paper shows the mean and standard deviation of these posteriors.

3.5 Results for Experiments 1 and 2

Details on Participant Recruitment Participants were recruited on Prolific (`prolific.co`). The task, advertised as "Read English Sentences by Pressing Keys", was only shown to residents of the US or the UK with English listed as their first language. Participants were asked what they considered adequate pay for the task, and listed amounts generally consistent with the pay offered. Data was collected by submitting a packet of data to a server upon completion of every sentence. For a small number of participants, less than 10 critical trials arrived at the server, either because they dropped out of the experiment or because not all packets of data were submitted successfully to the server. Those participants were paid, and their data were not excluded. In Experiment 1, data from one participant was not submitted to the server and lost entirely. In Experiment 2, experimenter error led to 17 extra participants being recruited. Their data is excluded when analyzing results specifically for Experiment 2 for consistency, but included in the pooled meta-analysis (Section 6.6 and Figure 23). This exclusion decision does not affect the qualitative or quantitative conclusions of the mixed-effects analysis.

Mixed-Effects Models We report the mixed-effects models for Experiments 1 and 2 in Figures 8-9.

We note that the data from Experiments 1–2 do not provide conclusive evidence for an effect of Embedding Bias or Compatibility within specifically the TWO condition. Substantial evidence for such effects within TWO is, however, provided when pooling data across these and previous studies, see Section 6.6 and Figure 23.

[7]Similar results are obtained when also taking into account the random effects for fact/report.

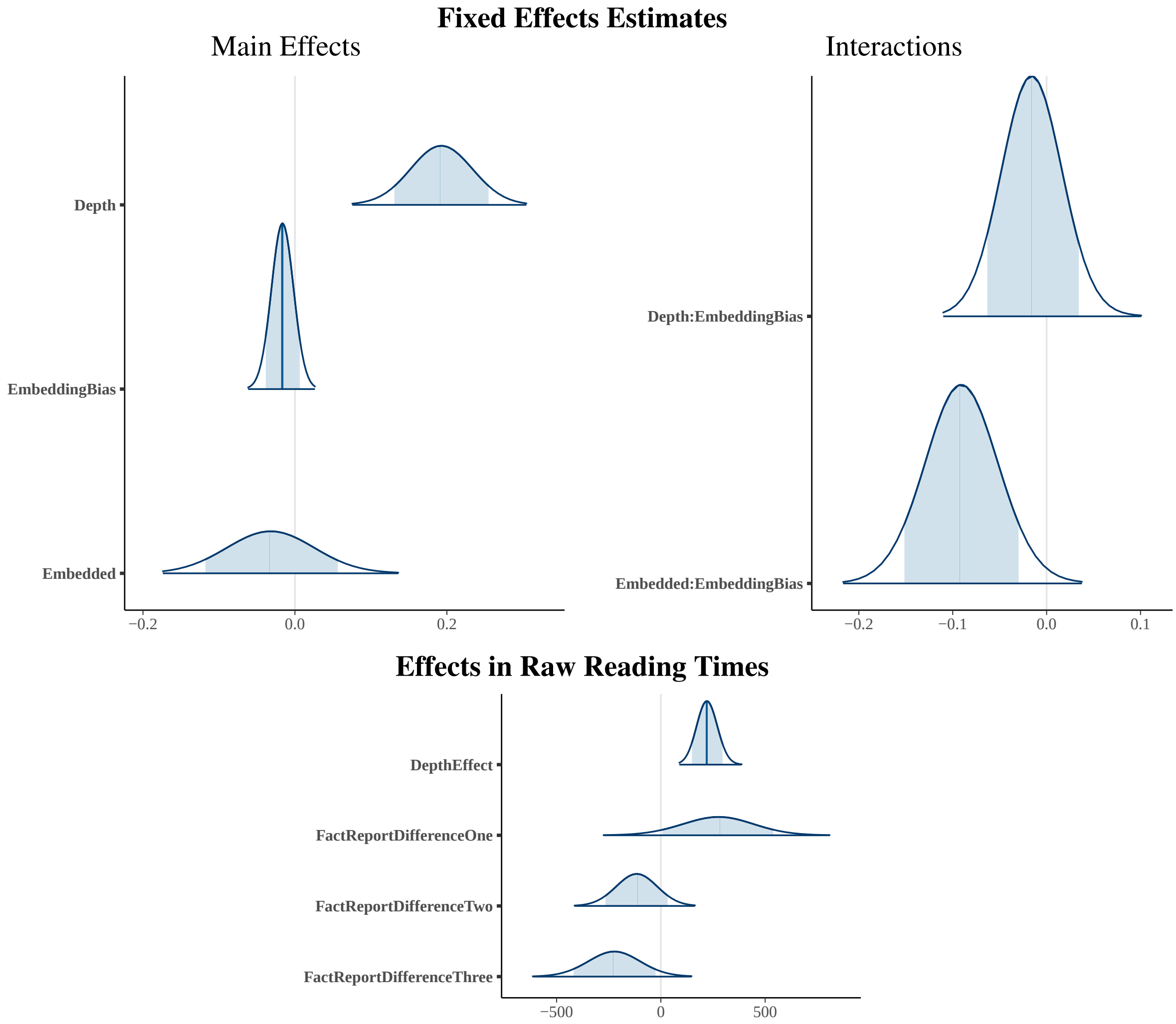

Figure 8: Top: Mixed-effects analysis of log-transformed reading times on the critical word in Experiment 1. We plot the posterior densities for the fixed effects coefficients, with symmetric 95% credible intervals. The key effects of theoretical interest are Depth and the interaction Embedding:EmbeddingBias. Bottom: Effects in raw reading times. Compare Figure 23 for a meta-analysis pooling all available evidence.

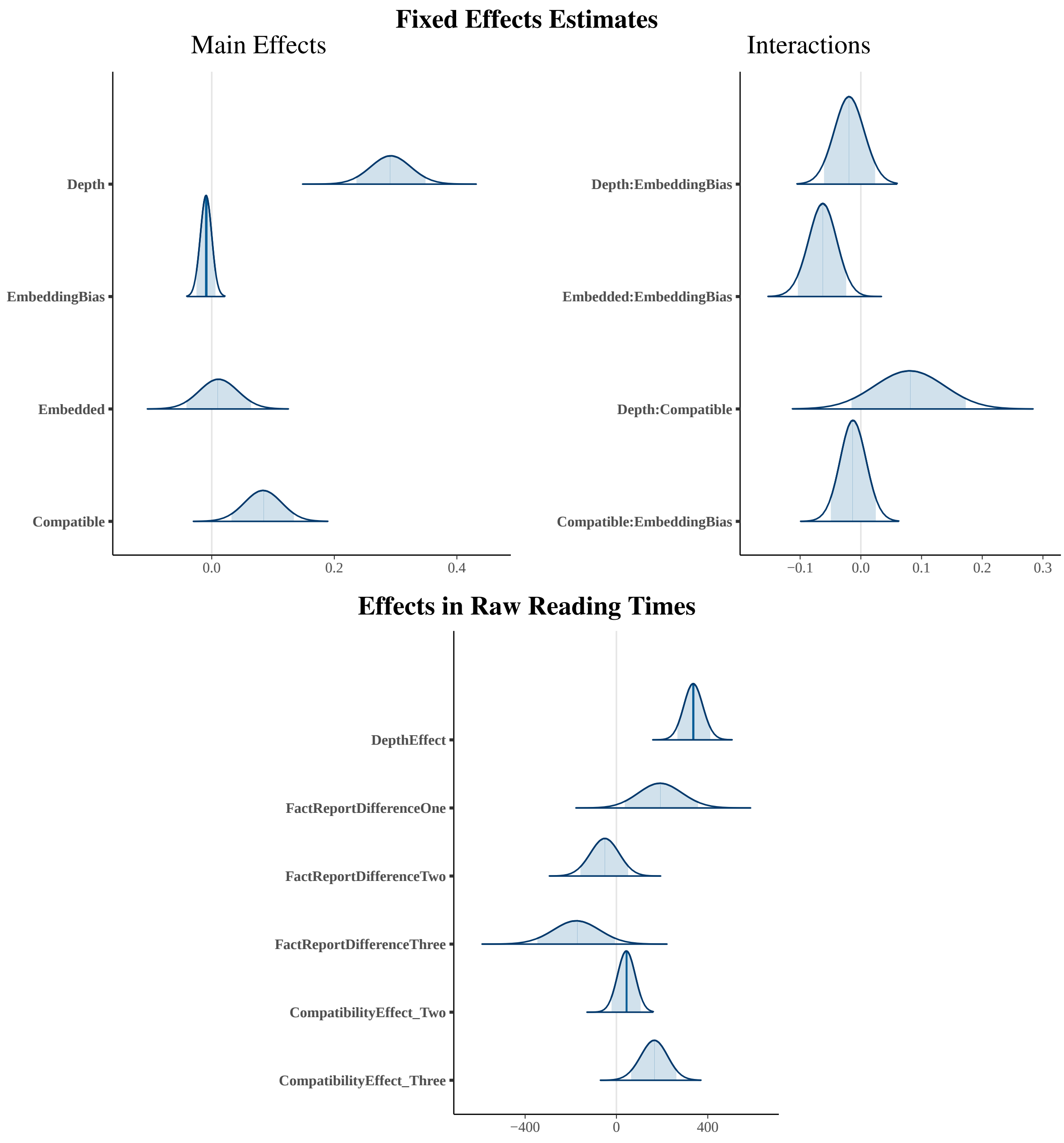

Figure 9: Top: Mixed-effects analysis of log-transformed reading times on the critical word in Experiment 2. We plot the posterior densities for the fixed effects coefficients, with symmetric 95% credible intervals. The key effects of theoretical interest are Depth, Compatible, and the interaction Embedding:EmbeddingBias. Bottom: Posteriors for effects in raw reading times. Compare Figure 23 for a meta-analysis pooling all available evidence.

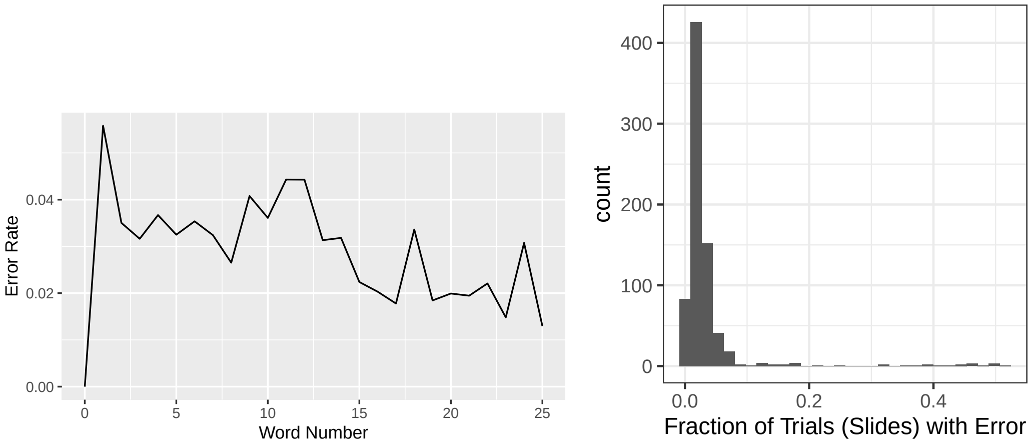

Figure 10: Error rates in the pooled data from Section 6.6. Left: Error rates by position in the sentence (before applying exclusion criteria). For comparison, Boyce et al. [7] report an error rate of 10% on the second word, and about 2–3% for words at position ≥ 5. Right: Fraction of words with error by participant. The vast majority of participants makes errors on less than 5% of words. The exclusion criterion affects participants making errors on more than 20% of words.

3.6 Analysis of Incorrect Responses

Figure 10 shows error rates by word number and participants in the pooled data from Section 6.6. Error rates are similar to Boyce et al. [7], and most participants make errors on less than 5% of words.

Errors across Conditions We analyzed answer correctness on the critical verb using the same fixed and random effects as we used for reading times, but with a logistic model, again implemented in `brms` with default priors. We conducted analyses on the pooled data as in Section 6.6. Results are shown in Figure 11. Errors increase in the difficult THREE condition, as shown by the effect of DEPTH. No other patterns find significant statistical support.

Reading Times conditioned on Errors We also conducted a version of the analysis on trials with both correct and incorrect responses, where we added a fixed effect for response correctness, and interactions with the predictors of primary theoretical interest, i.e., Compatible and Embedding Bias. Again, we report analyses on the pooled data as in Section 6.6. The resulting model fit is shown in Figure 12. There was a main effect of response correctness such that correct responses tend to be faster than incorrect ones, suggesting that errors reflect processing difficulty. Effects of Embedding Bias and Compatibility continue to be estimated very similarly to the model fitted on only correct trials (Figure 23 in Section 6.6).

3.7 Effect of Outlier Removal

Reaction time experiments can produce outliers, particularly due to repeated button pressing or due to participants being distracted or taking a break during a trial. Across the pooled data in Section 6.6, the minimum and maximum reading times recorded were 1 ms and 14 min, presumably due to rapid button presses and pauses within the experiment, respectively. As described in the main paper, we addressed this by excluding the top and bottom 1% of reading times (across critical items and fillers) from the dataset before every mixed-effects analysis. The resulting thresholds for exclusions (first and 99th percentile) were 428 ms and 3.5 s, respectively. 2.5% of the trials excluded due to this criterion were in the critical region, corresponding to 3.2% of the trials in the critical region.

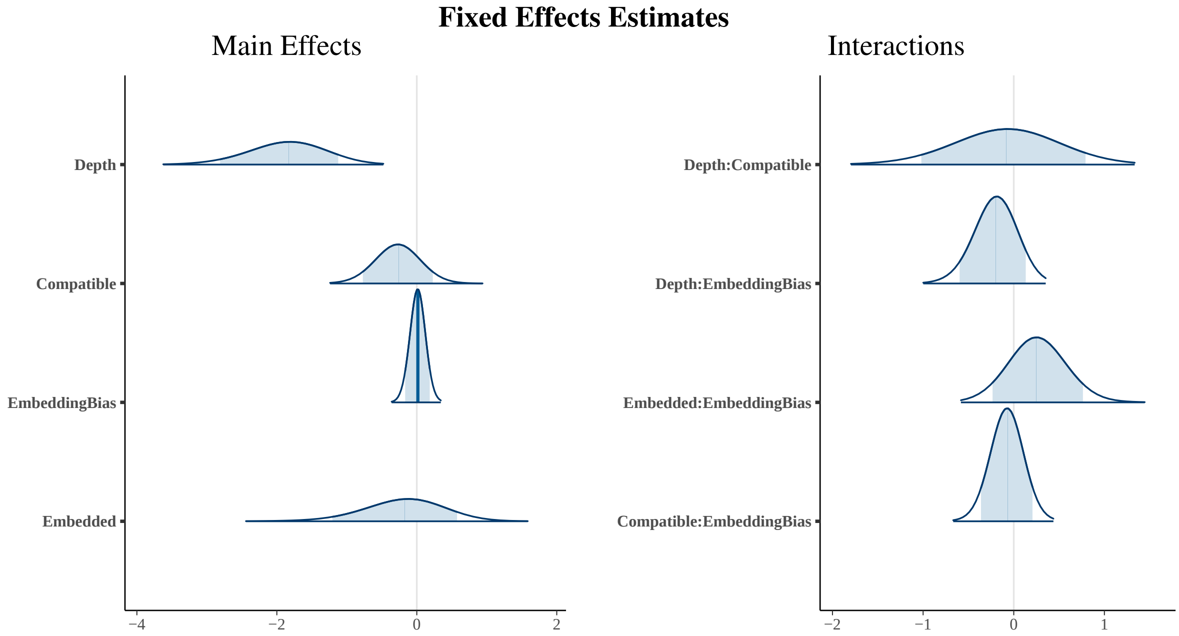

Figure 11: Logistic mixed-effects analysis of errors on the critical word in the pooled data from Section 6.6. Errors increase in the difficult THREE condition. No other patterns find statistical support.

We alternatively considered a more permissive criterion, where we removed reading times below 200 ms (percentile: 0.3%) and above 10 s (percentile: 99.9%). Results are similar to those from the other analysis, with larger effect sizes and sometimes stronger statistical evidence for our conclusions; we show those for Experiments 1 and 2 in Figures 13–14.

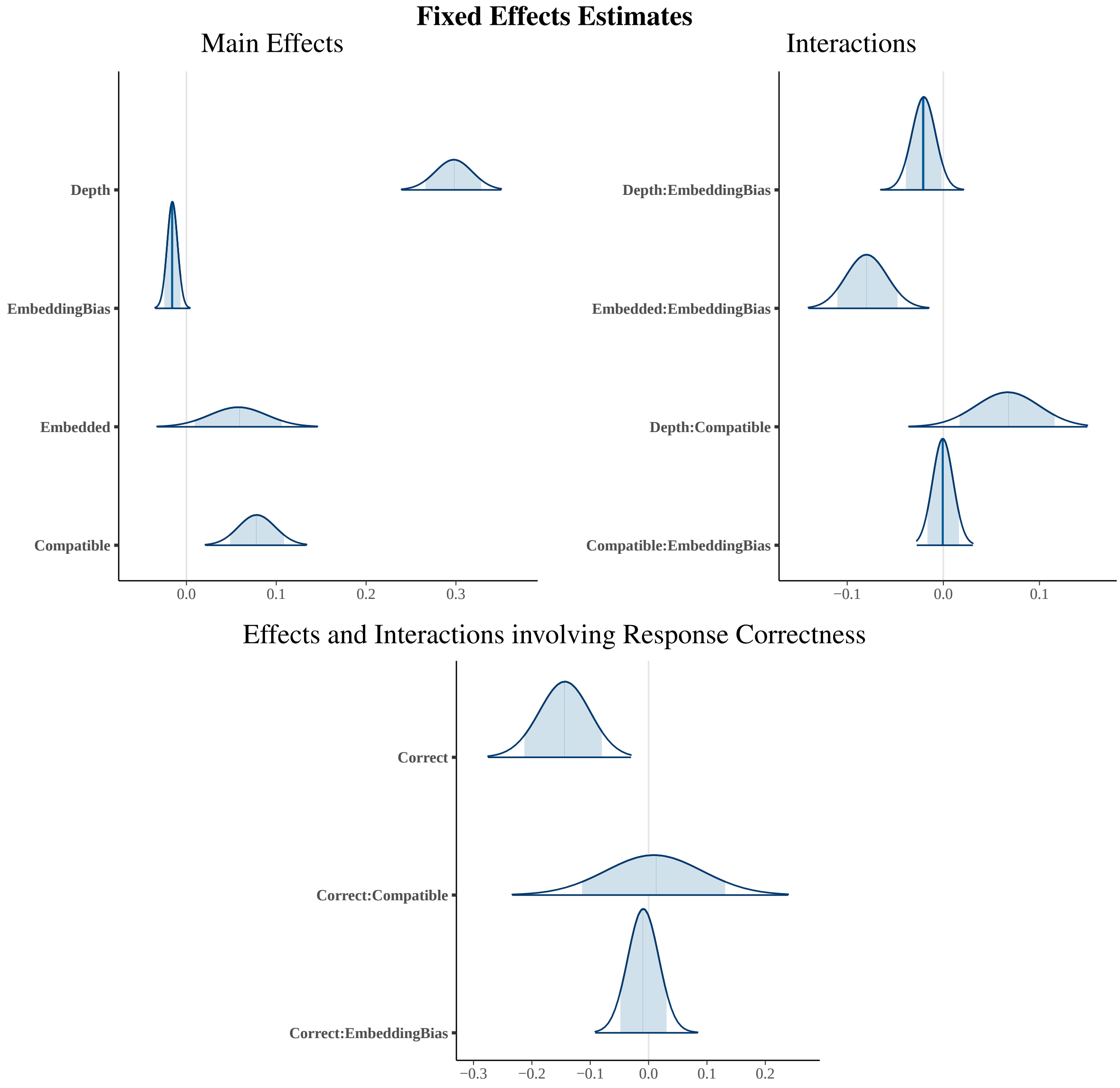

Figure 12: Experiment 2: Mixed-effects analysis of reading times on the critical word in the data from Section 6.6, including trials with an incorrect response, conditioned on response correctness. Correct responses tend to be faster than incorrect ones (main effect of CORRECT), suggesting that difficulty can manifest in both high reading times and incorrect responses. Response correctness did not impact the effects of compatibility or embedding bias. Compare Figure 23 in Section 6.6 for the corresponding analysis on the correct trials only.

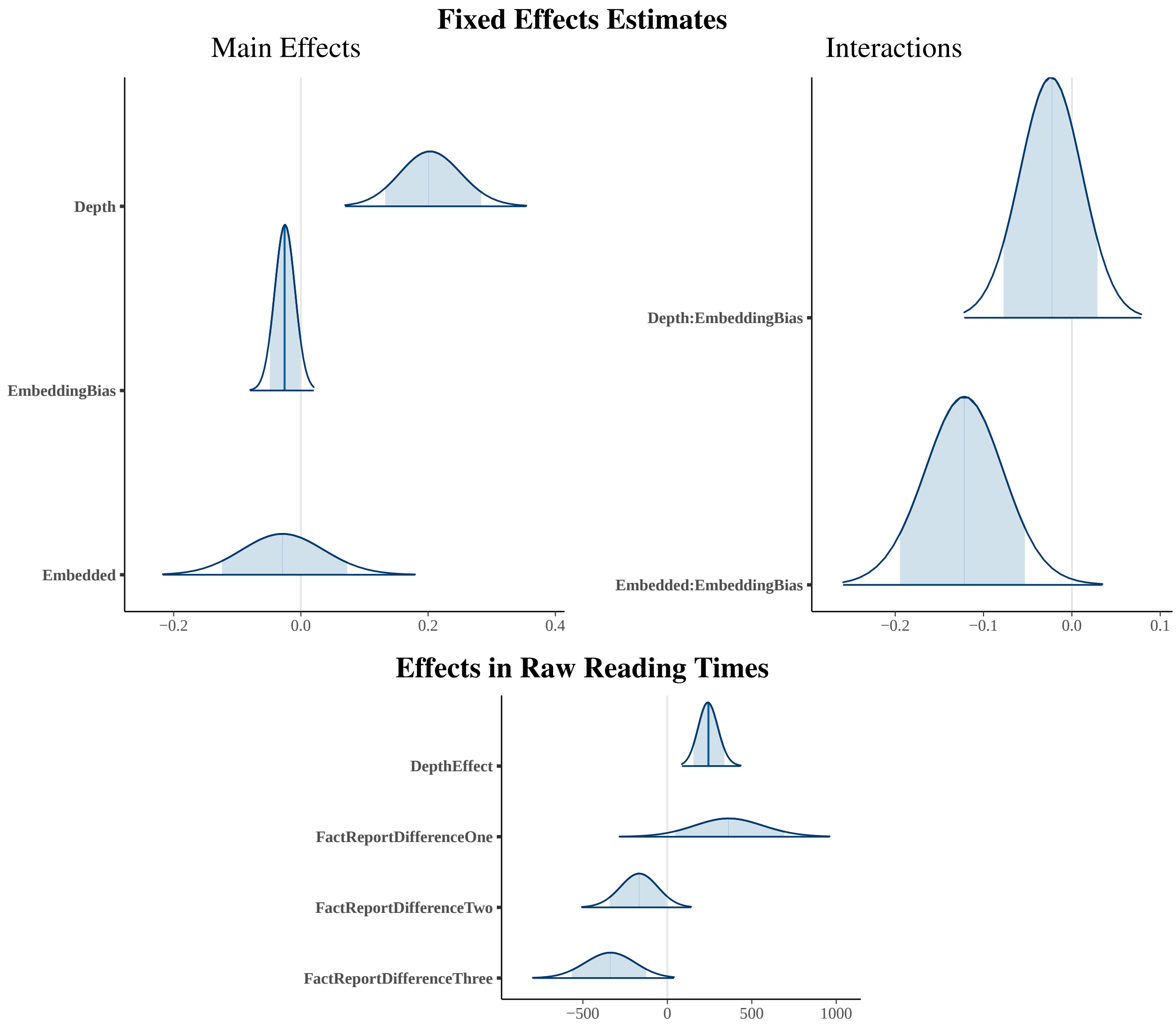

Figure 13: Alternative outlier removal: Mixed-effects analyses for Experiment 1, with more permissive outlier removal (removing reaction times below 200ms and above 10s, see Section 3.7). Compare Figure 8 for results with another criterion (removing top and bottom 1%). Statistical evidence for the key effects (Depth and the interaction Embedding:EmbeddingBias) is similar to the analysis in Figure 8, though effect size estimates are higher than in Figure 8.

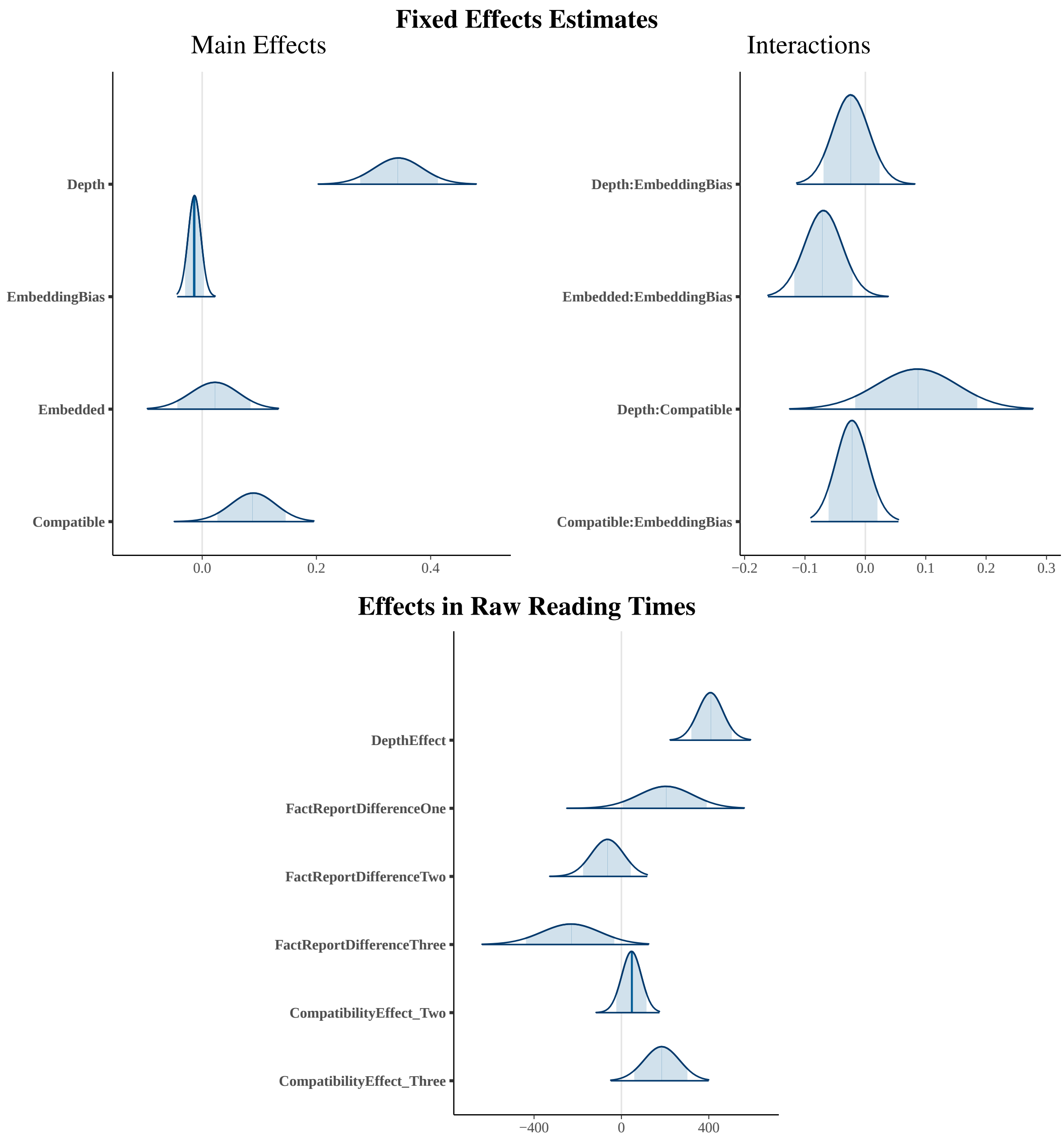

Figure 14: Alternative outlier removal: Mixed-effects analyses for Experiment 2, with more permissive outlier removal (removing reaction times below 200ms and above 10s, see Section 3.7). Compare Figure 9 for results with another criterion (removing top and bottom 1%). Statistical evidence for the key effects (Depth, Compatible, and the interaction Embedding:EmbeddingBias) is similar to the analysis in Figure 9, though effect size estimates are higher than in Figure 9.

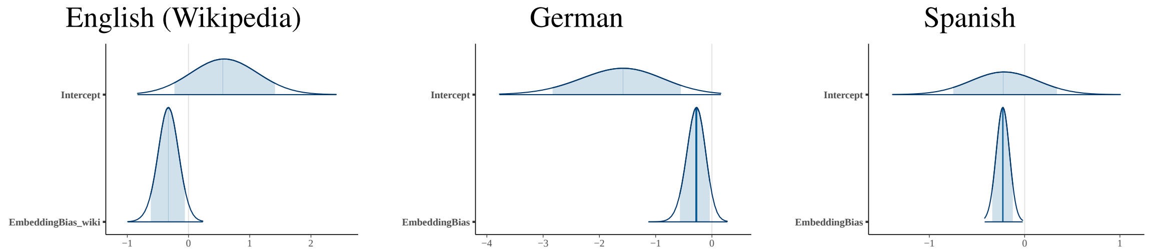

Figure 15: Posteriors for trial-by-trial logistic mixed-effects analyses of the production studies. The dependent variable is whether less than three verbs were produced. Variation in the intercepts reflects differences in the overall rate of incorrect responses; in particular, it is reduced in German in line with prior findings on center embeddings. The effect of interest is the effect of Embedding Bias, which is estimated at similar magnitude in all languages.

4 Additional Analyses for Experiment 3

Annotation Responses were left unannotated for the number of verbs when it was impossible to determine the number of verb phrases, primarily due to ungrammaticality beyond unambigously recoverable orthographic issues. In the English study, a fraction of subjects produced some or mostly ungrammatical completions; 17.6% of responses remained unannotated due to such issues. Such issues were not prominent in the other languages; 0% of Spanish and 2.3% of German responses remained unannotated.

Analysis and Results Logistic mixed-effects models were similar to those used for reading times (Section 3.3): Covariance matrices for random effects were parameterized as the combination of a correlation matrix and a vector of standard deviations [4]. We used a flat prior for the fixed effect coefficient, a Student's t prior with 3 degrees of freedom, location 0, and scale 2.5 for the intercept and the standard deviations, and an LKJ(1) prior for the random effects correlation matrices. We show posteriors of the analyses reported in the main paper in Figure 15.

See further Figure 32 for English results when estimating Embedding Bias on two other large corpora of English.

Part II
Further Experiments and Meta-Analysis

5 Ratings Studies

Previous work on center embeddings has shown that the difficulty of doubly-nested center embeddings (akin to the THREE condition) manifests itself not only in word-by-word reading times, but also in offline ratings. Here, the finding is that ungrammatical versions where the second-to-last verb is missing are perceived as relatively acceptable, sometimes even more acceptable than the full, grammatical version [21, 24, 11, 25, 20]. For instance, (2) is perceived as at least as acceptable than (1), even though it is ungrammatical, an effect known as *structural forgetting*, a kind of *grammaticality illusion* [21, 24]:

1. The patient who the nurse who the clinic had hired admitted met the doctor.

2. The patient who the nurse who the clinic had hired met the doctor.

It is generally accepted that the structural forgetting effect reflects processing difficulty on the third verb in (1) due to premature attachment of the second-to-last verb to the first noun [24, 44], corresponding to the behavior of the posterior in Resource-Rational Lossy-Context Surprisal discussed in the main paper. Thus, one should expect that Embedding Bias modulates the ratings difference between (1)-like and (2)-like sentences, such that humans prefer grammatical versions more strongly when Embedding Bias is higher. Such a prediction is not made by existing accounts of the forgetting effect, but is an immediate consequence of our model.

Here, we report offline ratings studies comparing grammatical and ungrammatical versions of the THREE condition, in an experimental setup closely following prior work on center embeddings [24], finding that ratings indeed exhibit the predicted effect.

5.1 Ratings Study S1

As in Experiments 1–2, trials paired items with nouns. Each item had two conditions, where the UNGRAMMATICAL condition is obtained by removing the second-to-last verb phrase:

1. GRAMMATICAL: The NOUN that the marketing whiz who the artist had hired was a fraud shocked everyone.

2. UNGRAMMATICAL: The NOUN that the marketing whiz who the artist had hired shocked everyone.

For each participant, 10 nouns and items were selected from pools of 34 nouns and 20 items, matched randomly. Critical trials were interspersed with 45 fillers, some of which included comprehension questions. Following [24], participants were asked to rate the *difficulty* of sentences ("How hard is this sentence to understand?").

This experiment consists of three sub-studies with successively more items and nouns (see Section 5.2 for a fully confirmatory replication). In the first part (N=9), there were twelve critical items and twelve nouns, randomly matched per participant. In the second part (N=99), the number of items was increased to 20, and the number of nouns to 21. In the third part (N=36), the number of nouns was increased to 34. Here, we analyze all data together.

We recruited 144 participants on Amazon Mechanical Turk. Stimuli, code, and data are deposited in the project repository.[8]

[8] `https://gitlab.com/m-hahn/resource-rational-surprisal/-/tree/main/experiments/rating/study1`

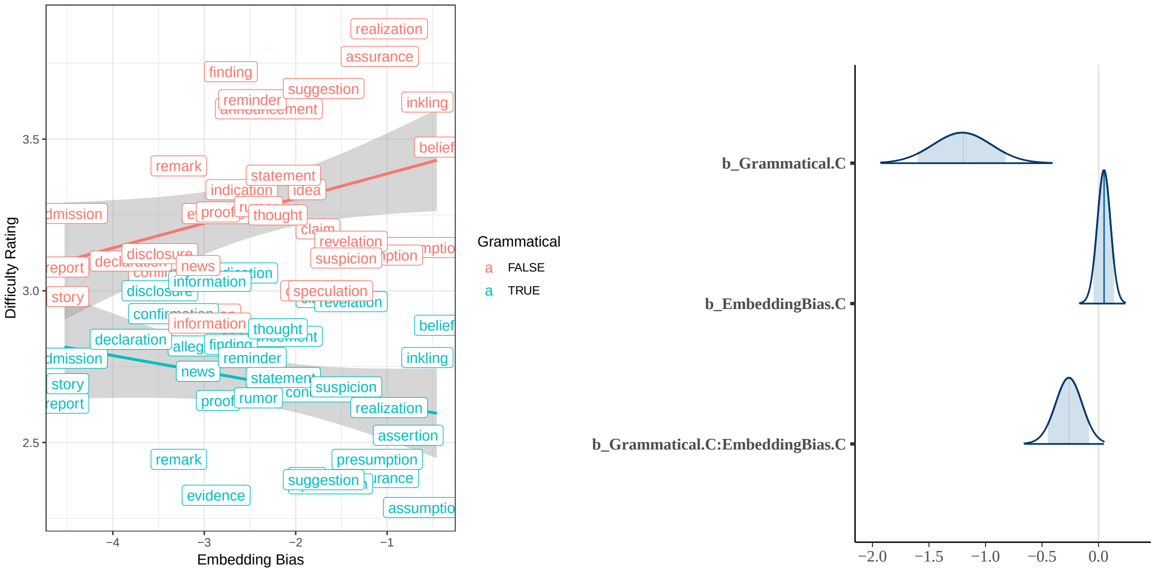

Figure 16: Ratings Study S1 (N=128). Left: By-noun mean responses in the two conditions. Right: Posterior of mixed-effects model. The key effect of theoretical interest is the interaction between grammaticality and Embedding Bias.

11% of participants answered less than 80% of comprehension questions correctly and were excluded from data analysis.

We analyzed the ratings using an ordinal mixed-effect regression, with brms default priors. We entered grammaticality and the embedding bias (both centered) as fixed effects, with random effects (including all appropriate random slopes) for subjects, nouns, and items. The effect of interest is the interaction between grammaticality and embedding bias.

Note that the stimuli in our experiments (THREE condition) differ from those studied in most prior work on center embeddings in that ours combine a complement clause with a relative clause (SC-ORC), whereas most work has studied stacked relative clauses (ORC-ORC). The latter kind of stacking is known to be even more difficult to comprehend than our THREE condition, a fact that is explained by various memory-based models of processing difficulty [23, 44]. We thus do not expect a full reversal of acceptability ratings as observed sometimes in previous studies; instead, we expect a main effect of grammaticality modulated by an interaction with Embedding Bias.

Results are shown in Figure 16. There was an effect of grammaticality, such that the UNGRAMMATICAL versions were rated more difficult to understand ($\beta = -1.20$, 95% CrI $[-1.59, -0.83]$). There was no evidence for a main effect of Embedding Bias ($\beta = 0.05$, 95% CrI $[-0.04, 0.13]$). The key interaction had the theoretically predicted sign, such that the effect of grammaticality increased as Embedding Bias increases ($\beta = -0.27$, 95% CrI $[-0.45, -0.09]$, $P(\beta > 0) = 0.0015$). This result matches the theoretical prediction.

5.2 Ratings Study S2

In Ratings Study S2, we replicated Ratings Study S1 with the following changes. First, we simplified the items so that the second noun phrase had only one word (as in the reading time studies). We also added 45 new fillers, and made minor changes to some fillers to address high error rates in comprehension questions in Ratings Study 1. Fourth, some changes were made to the set of nouns to specifically include nouns with very low and very high embedding bias to maximize statistical power.

191 participants were recruited on Prolific. One participant was excluded by the same criterion as in

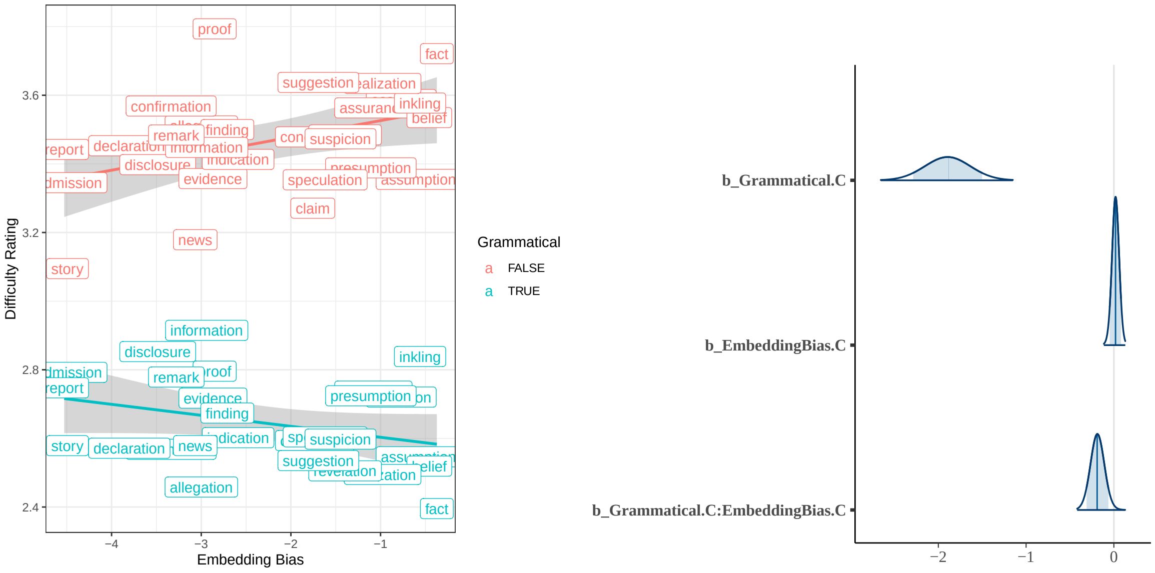

Figure 17: Ratings Study S2 (N=191): Replication with simpler stimuli. Note that the main effect of grammaticality is stronger than with the more complex stimuli (Experiment S1A), but the size of the interaction is similar.

Experiment S1. Stimuli, code, and data are deposited in the project repository.[9]

Data analysis was as in Ratings Study 1. Results are shown in Figure 17.

The interaction was estimated as $\beta = -0.19$, 95% CrI $[-0.31, -0.06]$, $P(\beta > 0) = 0.00275$, in agreement with the first experiment. Interestingly, the main effect of grammaticality was much stronger than in Ratings Study S1, which could be either due to the simpler stimuli or the change in participant population (Prolific vs Mechanical Turk).

[9] `https://gitlab.com/m-hahn/resource-rational-surprisal/-/tree/main/experiments/rating/study2`

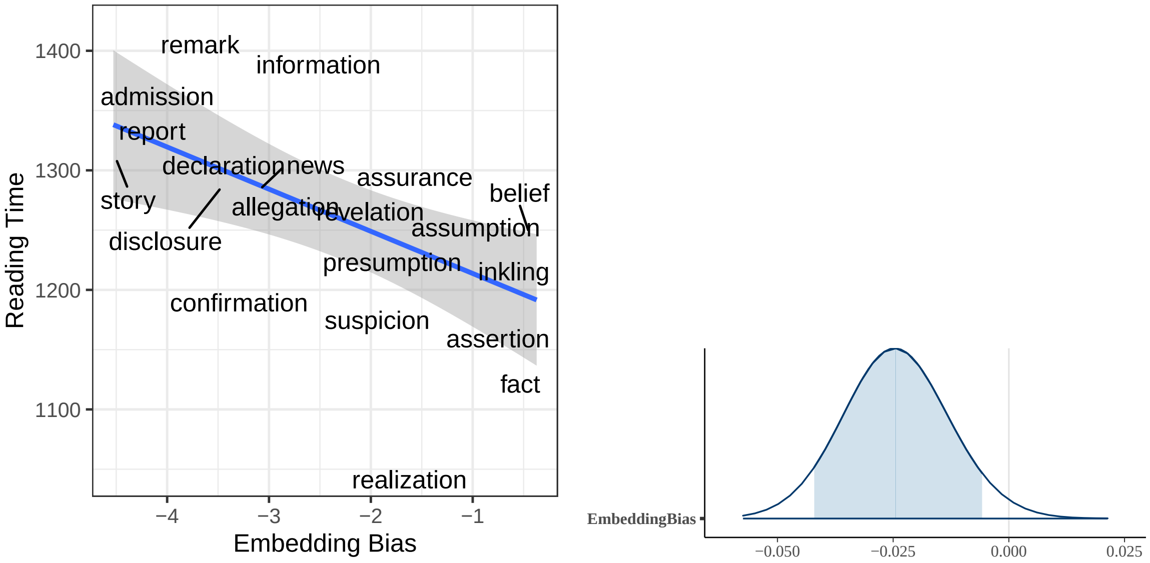

Figure 18: Experiment S1: Reading times within THREE (N=124). Left: Mean RTs per noun. Right: Posterior of embedding bias fixed effect in mixed-effects analysis of log-transformed RTs.

6 Previous Reading Time Experiments

Here, we describe prior reading time experiments and preregistrations. We initially measured the effect of Embedding Bias in the THREE condition (Section 6.1–6.2). We then preregistered theoretical predictions in the TWO and ONE conditions for Embedding Bias and compatibility (Sections 6.3–6.4). We then conducted a larger study examining all conditions, similar to Experiments 1 and 2 in the main paper, but with a partially different stimulus pool (Section 6.5). We also report a meta-analysis that combines evidence from this study with Experiments 1–2 (Section 6.6).

6.1 Experiment S1: Reading Times in THREE

We created 20 items and matched these randomly with 20 nouns for every participant.[10] The items were derived from those used in the ratings studies described in Section 5. All trials were in the THREE condition; no distinction was made based on semantic compatibility. The same fillers were used as in Experiments 1–2, but the full pool was used for every participant as there were 20 (instead of 10 as in Experiments 1–2) critical trials per subject.

Unlike Experiments 1–2, sentences were aborted when the subject made a mistake; this means that data is only available for those critical trials to which subjects advanced successfully. We recruited 124 self-declared English native speakers on Prolific.

The same exclusion criteria and data analysis were applied as in Experiments 1–2. For the given design, the only fixed effects predictor was Embedding Bias. Results are shown in Figure 18; they agree with the experiments described in the main paper, both in direction and size of the effect of Embedding Bias within THREE.

[10] Results are deposited in the project repository: `https://gitlab.com/m-hahn/resource-rational-surprisal/-/tree/main/experiments/maze/previous/study1_EmbeddingBias/Submiterator-master`.

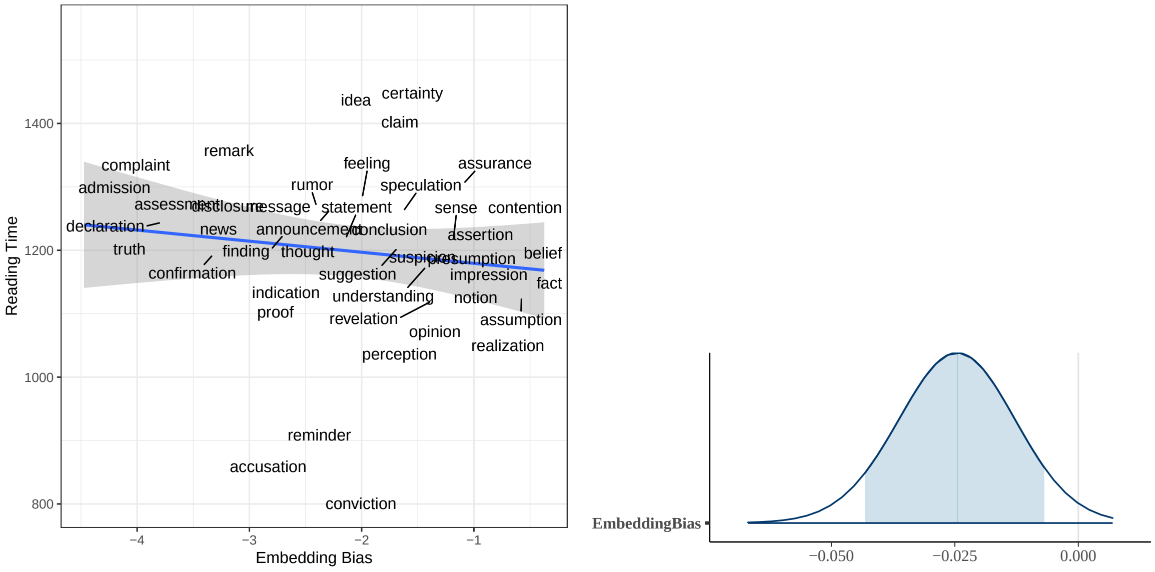

Figure 19: Experiment S2: Reading times within THREE+COMPATIBLE (N=113). Left: Mean RTs per noun. Right: Posterior of embedding bias fixed effect in mixed-effects analysis of log-transformed RTs. Note that measurement precision of per-noun means is reduced compared to other experiments because the number of nouns (46) is large, so that there are fewer data points per noun. However, the mixed-effects analysis shows strong evidence that the slope is negative, with a size agreeing with the other experiments.

6.2 Experiment S2: Reading Times in THREE+COMPATIBLE

This experiment was analogous to Experiment S2, but we changed the items so that the precritical verb phrase would be semantically compatible with the first noun.[11] We furthermore used a larger pool of 46 nouns, and constrained the matching of items and nouns as in Experiments 1–2. Unlike Experiment S1, we used the Maze version with do-overs as used in Experiments 1–2.

We recruited 113 self-declared English speakers on Prolific.

Results are shown in Figure 19; they replicate those of Experiment S1 and agree with those of Experiments 1–2.

6.3 Experiment S3: Reading Times in ONE and TWO

We conducted a preregistered study of the effect of Embedding Bias in the ONE and TWO conditions. We preregistered the theoretical predictions at `https://aspredicted.org/822i4.pdf`. For each subject, 5 trials were in the ONE condition and 5 trials in the TWO condition. We recruited 30 participants.

Stimuli, code, and data are deposited in the project repository[12].

The analysis included main effects of Embedding Bias and of the EMBEDDED contrast (TWO vs ONE contrast). We conducted the same mixed-effects analysis as in Experiments 1–2.

Results are shown in Figure 20; they are consistent with those in Experiments 1–2. Note that reading times are lower than in those experiments; this can be attributed to the fact that a larger fraction of stimuli in this early study had the frequent word *was* as the critical word.

[11] Results are deposited in the project repository: `https://gitlab.com/m-hahn/resource-rational-surprisal/-/tree/main/experiments/maze/previous/study2_compatible/Submiterator-master`.

[12] `https://gitlab.com/m-hahn/resource-rational-surprisal/-/tree/main/experiments/maze/previous/study3_OneTwo`

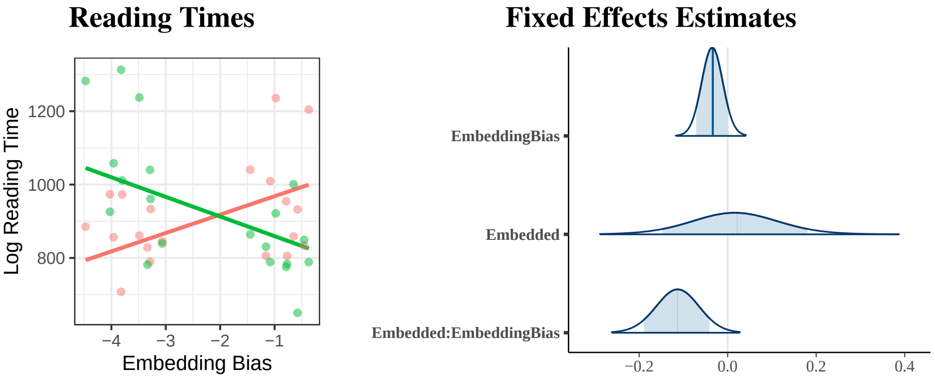

Figure 20: Experiment S3: Reading times in ONE and TWO (N=30).

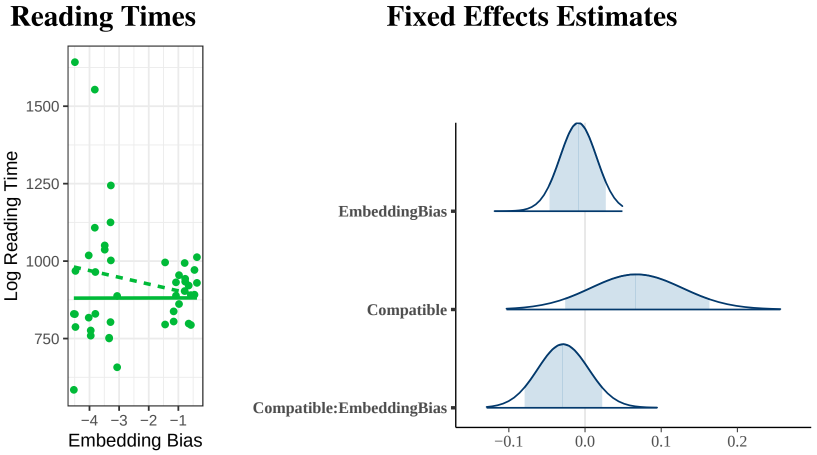

Figure 21: Results from Experiment S4 (N=40).

6.4 Experiment S4: Effect of Compatibility on Reading Times

Here, we report our initial study of the effect of Compatibility in the TWO and THREE conditions. We preregistered the theoretical prediction at `https://aspredicted.org/88tk4.pdf`. We preregistered investigating compatibility in both TWO and THREE, but, due to a scripting error, the experiment only had trials in the TWO condition and none in the THREE condition. The items were derived from those of the previous study. As preregistered, we recruited 40 subjects.

Stimuli, code, and data are deposited in the project repository[13]. Results are shown in Figure 21. They are consistent with those in the main paper, but do not provide conclusive statistical evidence alone due to the small number of participants.

6.5 Experiment S5: Large-Scale Replication of Experiments S3 and S4

We next conducted a series of replications of Experiments S3 and S4, using larger sets of items (69) and nouns (40). In particular, in addition to items from the previous experiments, 32 items were constructed specifically such that the precritical word was constant across the COMPATIBLE and INCOMPATIBLE conditions. We recruited 431 subjects on Prolific. This experiment consisted of multiple studies with successively larger pools of items that we analyze together here. As in Experiments 1–2, there were 10

[13]`https://gitlab.com/m-hahn/resource-rational-surprisal/-/tree/main/experiments/maze/previous/study4_compatibility/`

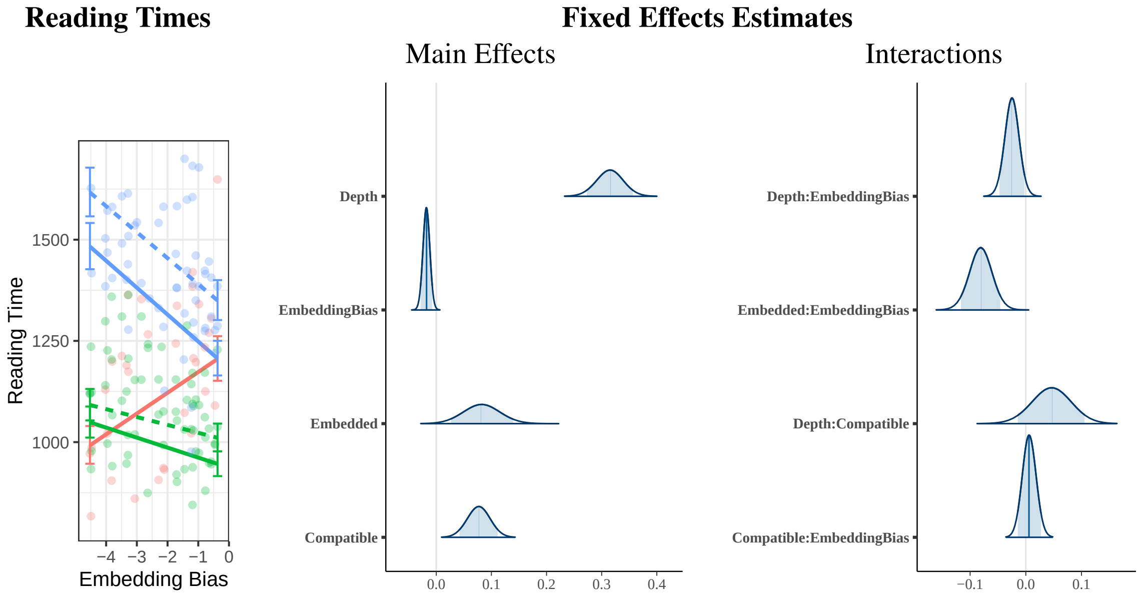

Figure 22: Results from Experiment S5 (N=431). We show the posterior of the fixed effects in the mixed-effects model predicting log-transformed reading times (right), and reading times (left). As in Figure 3 in the main paper, we show empirical means per noun and condition, and mean reading times and standard deviations at "fact"-like and "report"-like nouns, all derived from the mixed-effects analysis. Results closely agree with those in Experiments 1–2, compare Figures 8–9.

critical trials and 30 fillers per participant.

Stimuli, code, and data are deposited in the project repository[14].

Experiments 1 and 2 in the main paper constitute replications of this study, but differ in that the stimulus pools were modified to avoid certain potential concerns regarding in the compatibility manipulation (see Section 12).

We show reading time results in Figure 22. Results agree closely with those of Experiments 1 and 2 (compare Figures 8–9).

6.6 Meta-Analysis for Reading Time Studies

Here, we synthesize evidence from all large-sample reading time experiments covering ONE, TWO, and THREE (Study S5, Experiments 1–2), in order to provide as precise estimates of effect sizes as possible. High-precision estimates are useful both for comparison with theoretical models, and to inform future studies building on the effects reported in this paper.

There were a total of 119 items, including 32 appearing in Experiment 1, 42 items appearing in Experiment 2, and 45 items that only appeared in Study S5 and were not included in Experiments 1–2 (see Section 12). The experiments had a total of 747 subjects, each with data from 10 critical trials.

Fixed-effects estimates and effects in raw reading times are shown in Figure 23. Estimates sharpen those from the individual experiments. In particular, effects of Embedding Bias and Compatibility are confirmed within both TWO and THREE individually:

1. effect of depth $\widehat{\Delta} = 335$ ms, 95% CrI $[294, 377]$, $Pr(\widehat{\Delta} < 0) < 0.0001$

[14] https://gitlab.com/m-hahn/resource-rational-surprisal/-/tree/main/experiments/maze/previous/study5_replication

2. Difference between 'fact' and 'report' in ONE: $\widehat{\Delta} = 222$ ms, 95% CrI $[96, 347]$, $Pr(\widehat{\Delta} < 0) < 0.0001$

3. Difference between 'fact' and 'report' in TWO: $\widehat{\Delta} = -89$ ms, 95% CrI $[-146, -33]$, $Pr(\widehat{\Delta} < 0) = 0.004$

4. Difference between 'fact' and 'report' in THREE: $\widehat{\Delta} = -242$ ms, 95% CrI $[-337, -145]$, $Pr(\widehat{\Delta} < 0) < 0.0001$

5. Compatibility effect in TWO: $\widehat{\Delta} = 39$ ms, 95% CrI $[1, 76]$, $Pr(\widehat{\Delta} < 0) = 0.02$

6. Compatibility effect in THREE: $\widehat{\Delta} = 141$ ms 95% CrI $[86, 198]$, $Pr(\widehat{\Delta} < 0) < 0.0001$

Interactions DEPTH:EMBEDDINGBIAS and DEPTH:COMPATIBLE (Figure 23) indicate that effects of Embedding Bias and Compatibility are stronger in THREE than in TWO, in agreement with model predictions at moderately high retention rates (Figure 4). This is also shown by the fact that the credible intervals for the effects in TWO and THREE are essentially disjoint.

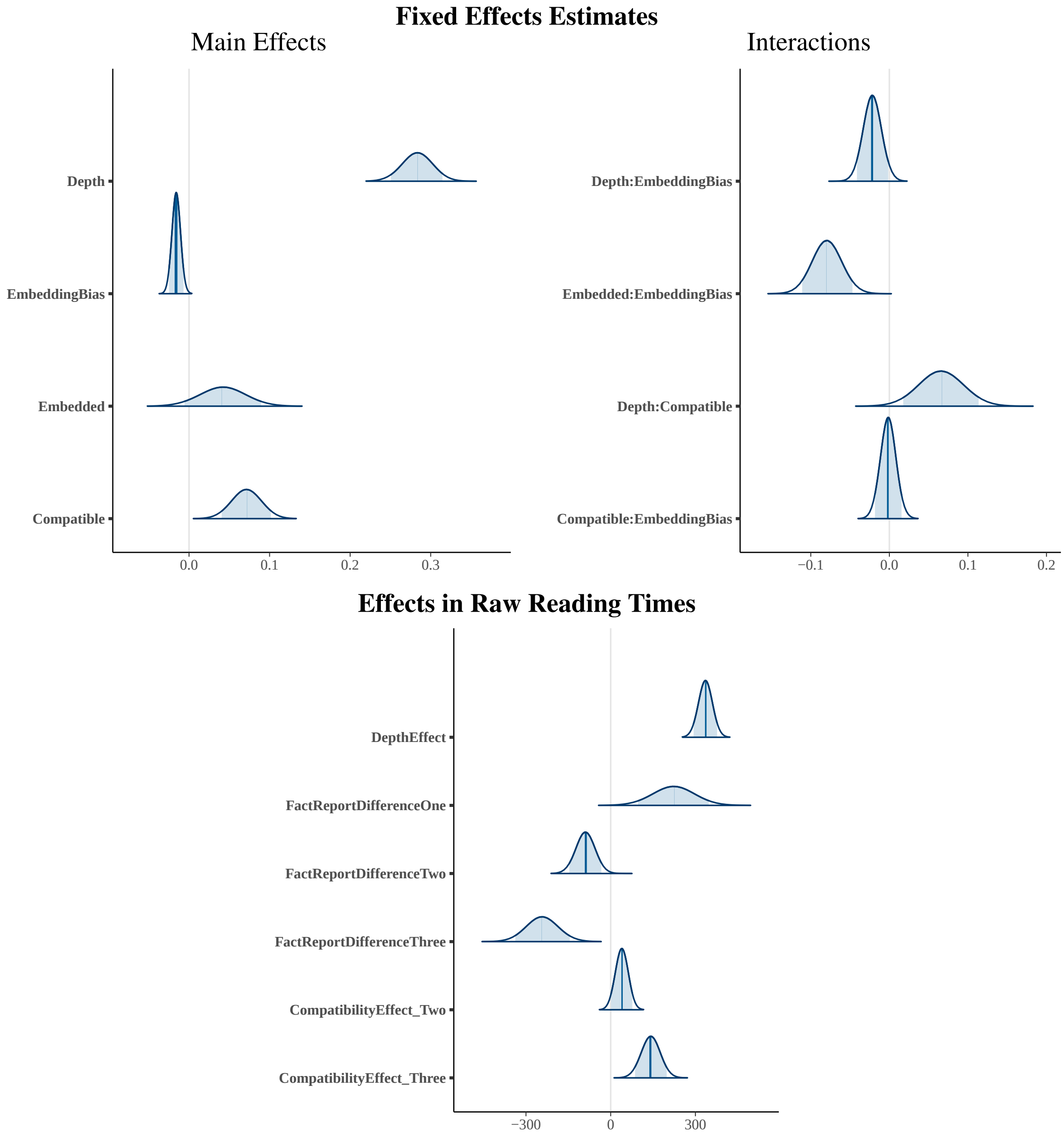

Figure 23: Results from meta-analysis (N_{subjects}=747, N_{items}=119). Top: Posteriors of fixed-effects coefficients for mixed-effects analysis of log-transformed reading times. Bottom: Effects transformed into raw reading times.

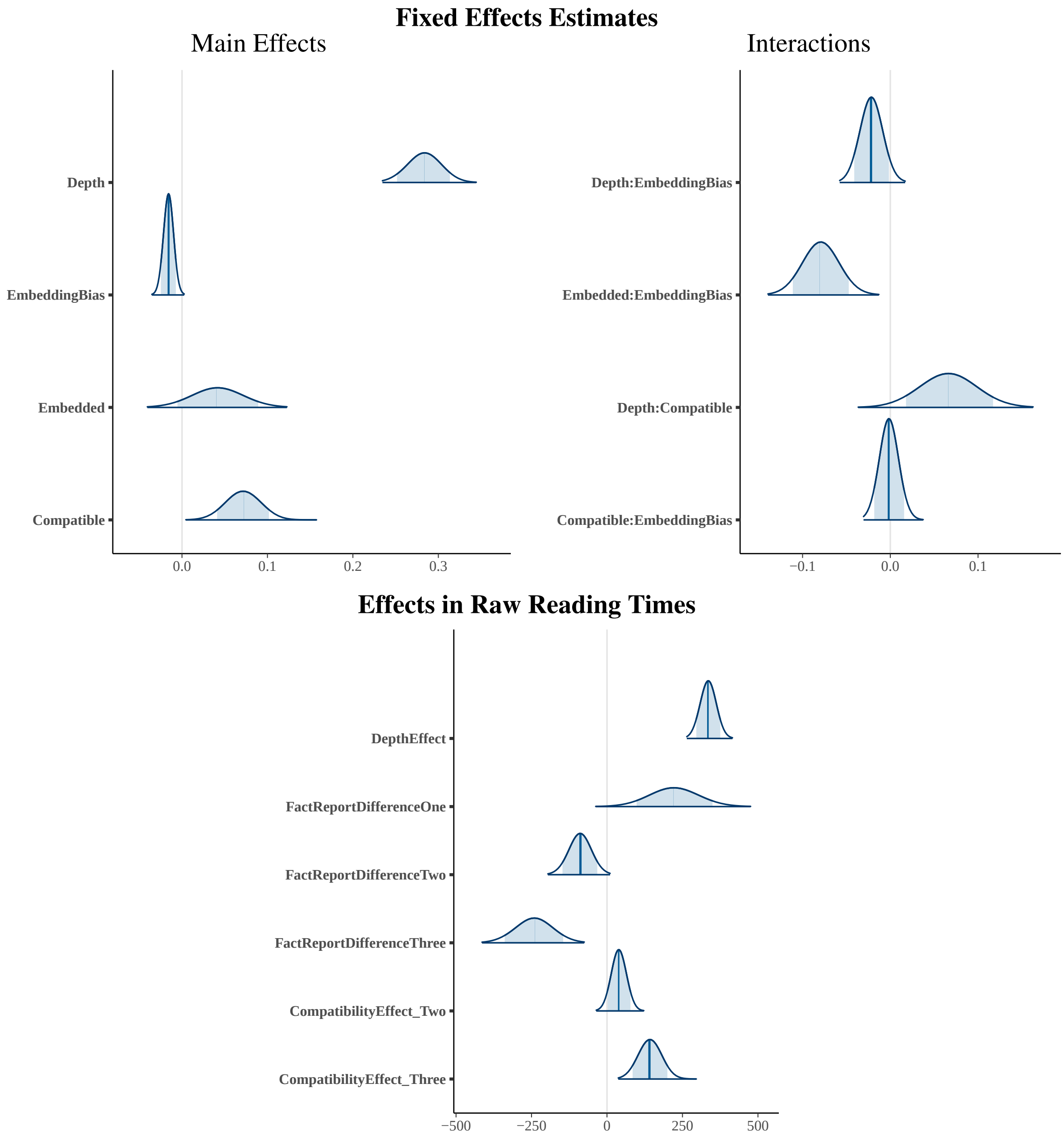

Figure 24: Results from meta-analysis (N_{subjects}=747, N_{items}=119), analyzed with a mixed-effects analysis with a strongly regularizing prior $\mathcal{N}(0,1)$ for the fixed effects. The other parameters have the same prior as the analysis in Figure 23: $t(3,7,2.5)$ for the intercept, $t(3,0,2.5)$ for the variance components, $LKJ(1)$ for the correlation matrices. Results are essentially identical to those in Figure 23.

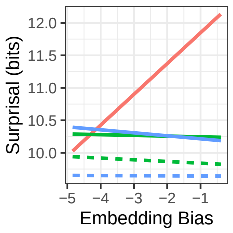

Figure 25: Surprisal computed from fully veridical context representations (corresponding to $\delta = 20$) as computed by GPT-2-XL, the largest version of GPT-2. Compare Figure 3 (right, Surprisal Theory) in the main paper for corresponding results from GPT-2-Medium.

Part III
Comparison with Other Models and Theories

7 Comparison with other Theories

7.1 Conceptual predictions for DLT and Surprisal Theory

To derive conceptual predictions from DLT, we computed Integration Cost on the last verb. This depends on the number of discourse referents between the last verb and the first noun, which is 0 in the One condition, 2 or 3 (depending on the precise sentences, e.g., 3 in *the report that the **doctor cured** the **patient***) in the Two condition, and 4 or 5 (depending on the precise sentences, e.g., 5 in *the report that the **doctor** who the **senator admired cured** the **patient***) in the Three condition.

This qualitative prediction is shared by memory-based models more broadly, as they fundamentally predict that dependencies are harder to resolve when more material intervenes [47, 48, 44]. Models also make specific predictions about how this cost is modulated, in particular due to similarity-based interference, but this does not predict effects related to Embedding Bias (see Section 7.2). On a conceptual level, models in the cue-based retrieval paradigm may potentially predict an effect of semantic compatibility (see Section 7.2), but this does not arise in computationally explicit models in in this paradigm [44, 55, 17].[15]

For Surprisal Theory, we first noted that, when embedding bias is very high, the chance that a noun is followed immediately by a verb has to be relatively low. We thus predicted a positive main effect of embedding bias on surprisal of the last verb in the ONE condition. On the other hand, in the TWO and THREE condition, after observing a clause, we do not expect that the chance of observing another verb depends on either embedding bias of semantic compatibility. To the extent that Surprisal Theory may predict a difference between the TWO and THREE conditions, it would be in the *opposite* direction of the empirically observed one (lower surprisal in the THREE condition), as additional intervening material should make prediction of the final verb easier. These conceptual predictions are in agreement with the surprisals computed both by GPT-2 Medium as reported in the main paper, and by GPT-2 XL, the strongest model in the GPT-2 family, at zero noise (Figure 25). Note that the effect of compatibility is numerically even in the opposite direction compared to Resource-Rational Lossy-Context Surprisal and human reading times.

[15] We are grateful to Jakub Dotlačil for help with running the model from Dotlačil [17].

7.2 Implications for Cue-Based Retrieval Accounts of Center Embedding Difficulty

The difficulty patterns observed in our experiments are distinct from the predictions of existing theories of sentence processing. The findings thus pose an interesting challenge for previous, more mechanistic models of sentence processing, constraining the search for future models that could account for our findings within those modeling frameworks. Here, we discuss prospects and challenges for accounting for our experimental results within a prominent family of previous approaches based on a left-corner parser using associative retrieval as proposed by Lewis and Vasishth [44] (similarly Rasmussen and Schuler [55], Dotlačil [17]).

In the literature on cue-based retrieval, the difficulty of center embeddings arises because syntactic structures are established not using order information (as in a stack), but using associative retrieval of content-addressable memory structures [48, 44, 52]. When encountering the second-to-last verb in a center embedding structure, humans may erroneously retrieve the wrong noun as the subject of this verb, leaving the final verb stranded [48, 44, 34]. Conceptually, this idea is closely related to our model, where processing is disrupted when the information retrieved from short-term memory suggests attaching the second verb to the first noun with relatively high posterior probability. Lewis and Vasishth [44] describe a computationally implemented model of sentence comprehension that also includes an account of the difficulty of center embeddings. In this model, retrieval failure frequently occurs at the last verb because the prediction of a main clause verb was retrieved too early, leaving it unavailable when the final verb is encountered [44, p. 406].[16]

In order to account for our empirical results, such models thus need to be extended to predict in detail when the correct noun is more or less likely to be retrieved as the subject of the second-to-last verb. Two effects need to be accounted for specifically, namely the effects of semantic compatibility and of embedding bias in the TWO and THREE conditions.

An account of the effect of semantic compatibility seems relatively straightforward to implement within extensions of such models. On an informal level (without formalization or computational implementation), Häussler and Bader [34] propose a *Discrimination Hypothesis*, which states that integration of new material is disrupted when a clause intervenes after the correct integration site and "an incorrect but similar integration site competes for attachment." To account for the difficulty of doubly-nested center embeddings (such as our THREE condition), they propose that, when the second verb is encountered, the human parser erroneously retrieves the first (instead of the second) noun as the subject. They also argue that the proposed Discrimination Hypothesis accounts for previous observations on the role of semantic properties of the second verb phrase [24], related to the effect of the Compatibility manipulation in our experiments. In Figure 26A, we show the syntactic structure assigned to a stimulus in the TWO condition in the grammatical formalism assumed by Lewis and Vasishth [44], based on X-Bar syntax [38]. Figure 26B (left) illustrates the state of the parser before the word "annoyed" is received; each constituent in the syntactic tree is represented by a feature structure stored as a chunk in short-term memory (formalized in the ACTR-R framework, Anderson [3]), including information about syntactic categories, lexical items, and which other chunks a chunk is linked to in the syntactic tree. The current tree has two open predictions that both require a finite verb, each represented by a chunk stored in memory. As the parser relies on associative retrieval, and does not represent linear order, it needs to rely on other cues to identify which of the two nodes to attach "annoyed" to. In this case, both subjects are semantically compatible with the verb, and the parser may (incorrectly) attach "annoyed" to VP_1. The parser then completes VP_1 and ends up believing that IP_1 is complete, leading to breakdown once "was" is observed (cf. Lewis and Vasishth [44, Section 6.3]). In contrast, when replacing "annoyed" with "cured", semantic cues strongly favor attaching "annoyed" to VP_2, avoiding prematurely declaring the sentence complete.

[16]While their model as implemented did not actually show retrieval failure in the structures corresponding to our THREE condition (SC/RC in their terminology) (they do not report results for the TWO condition), we believe that the parser rules in their model could be adapted to produce it in the model predictions.

This mechanism could account for the effect of compatibility on reading times on the final verb; a full account would require a detailed specification of parser rules that percolate information about the subject through the tree and use it to determine attachment sites.

It is much less clear how the cue-based retrieval proposals might be extended to account for the effect of embedding bias: it is not obvious why embedding bias should determine how likely a noun is to be retrieved as the integration site for a subject-verb dependency, as embedding bias does not determine a noun's chance of being a subject. One possibility is that the content of the chunks themselves is subject to loss, so that the CP node may be incorrectly recovered as a prepositional phrase node, depending on semantic and statistical cues. Another possibility – one we believe might be the most fruitful in searching for extensions of the Lewis and Vasishth [44] model – is that parser rules are sensitive to statistical patterns in the language. The parser rules in the parser implemented by Lewis and Vasishth [44] are not sensitive to the lexical difference between report/fact/..., but a rational parser with fine-grained awareness of the statistics of the language might well have such rules. Indeed, given the fundamentally rational and adaptive nature of memory retrieval in the Lewis and Vasishth [44] model, it is an appealing idea that memory retrieval should be impacted by the statistical structure of language and give rise to patterns resembling noisy-channel inferences. In a situation such as in Figure 26B, a rational parser may have a specific rule stating – for instance – that, if there are two open VP predictions and one of them has as its subject a noun with very high embedding bias (such as "fact"), a new verb should be preferentially attached to the other VP, as that is statistically likely to be inside a complement clause headed by that noun. Such a rule would then prevent premature attachment to the top-level node. Again, working out a full account would require specifying suitable parser rules, a nontrivial undertaking which may be an interesting problem for future work in the cue-based parsing paradigm.

We have seen that, generally speaking, the difficulty of center embeddings arises in associative retrieval-based parsing models because the parser makes choices based on local information available at the relevant chunks, "guessing" the next steps without a costly traversal of the full tree necessary to gain certainty about the correct next action [48, 44, 34]. The effect of words (e.g. "that") *not being retained* in Resource-Rational Lossy-Context Surprisal corresponds to the effect of words and chunks (e.g., CP_1 in Figure 26B) *not being retrieved* when processing the next word in associative retrieval-based parsers. We suggest that cases where the posterior $P(c|c')$ assigns *high probability to structurally nonveridical variants* correspond to cases where parser rules make *incorrect inferences about the structural position* of the next word by relying on a limited number of easily-retrieved chunks. If the rules of a symbolic parser are rationally adapted to the statistics of the language, then the *influence exerted by surface-similar variants on expectations* in lossy-context surprisal should correspond to rationally adaptated grammar rules *attempting to infer the structural position of the next word based on limited information and the statistics of the language*. Quite similarly to the low on-average surprisal achieved by resource-rational memory representations in our model, such heuristic choices based on local information may be rational on most sentences, but lead to breakdown in structures such as center embeddings. We believe that these suggested correspondences may prove fruitful in searching for extensions of cue-based parsing models that can emulate the predictions of Resource-Rational Lossy-Context Surprisal.

A key difference between our proposed resource-rational model, and models of retrieval-based parsing such as that of Lewis and Vasishth [44] is that the resource-rational objective function, coupled with the definition of lossy-context surprisal (Equation 1 in the main paper), and a specification of the average retention rate, are sufficient to derive model predictions on any input. In contrast, for a retrieval-based parser, predictions are substantially dependent on the grammar and the parsing rules. Whereas the effects observed in our experiments "come for free" under the resource-rational model, implementing them in a retrieval-based parser requires some, potentially arbitrary, decisions about grammar and parser rules. We believe that identifying a rational modeling approach that could derive grammar and parsing rules in a principled manner is an interesting problem for future research, and might point the way towards models recapitulating the predictions of our resource-rational model within more mechanistic symbolic

parsing models. More generally, in agreement with Parker et al. [52], we believe that finding cue-based retrieval models that are fully integrated with findings about probabilistic processing is an important open problem for psycholinguistic research. Such models could provide algorithmic-level counterparts to the computational-level model that we advance in this paper; the findings we describe constrain the search for such models.

7.3 Other Objective Functions

We assumed that memory optimizations are optimized for next-word prediction. Here, we compare to a model that instead assumes that representations are optimized to accurately represent the past, i.e., maximize the mutual information between the context c and the representation c':

$$\min_\theta \mathbb{E}_{c^* w} \mathbb{E}_{c' \sim p(c'|c^*)} \left[-\log p(c|c') \right] \tag{20}$$

where $p(c|c')$ is the posterior as displayed in Figure 2 in the main paper. We solved this objective function using the same method as for our model, representing $p(c|c')$ using the inference network $q_\varphi(c|c')$ in formulating the variational Lagrangian objective function (Equation 6).

Resulting model surprisal is shown in Figure 27. The model continues to qualitatively predict the three effects of interest: Surprisal is higher in THREE than in TWO, decreases with increased embedding bias in the TWO and THREE conditions, and is higher in COMPATIBLE conditions than in INCOMPATIBLE conditions. The difference between TWO and THREE is reduced compared to the model optimizing for predictability, and compared to human reading times. We attribute this to the fact that the objective (20) does not favor retaining recent elements more strongly, and therefore underpredicts locality effects. While this locality effect is predicted by most theories of memory in sentence processing, the other two effects (embedding bias and compatibility in TWO/THREE) are not. It is therefore striking that the model (20) strongly exhibits those, suggesting that they are quite robust to modeling assumptions within the broader framework combining Lossy-Contect Surprisal with resource-rational context representations.

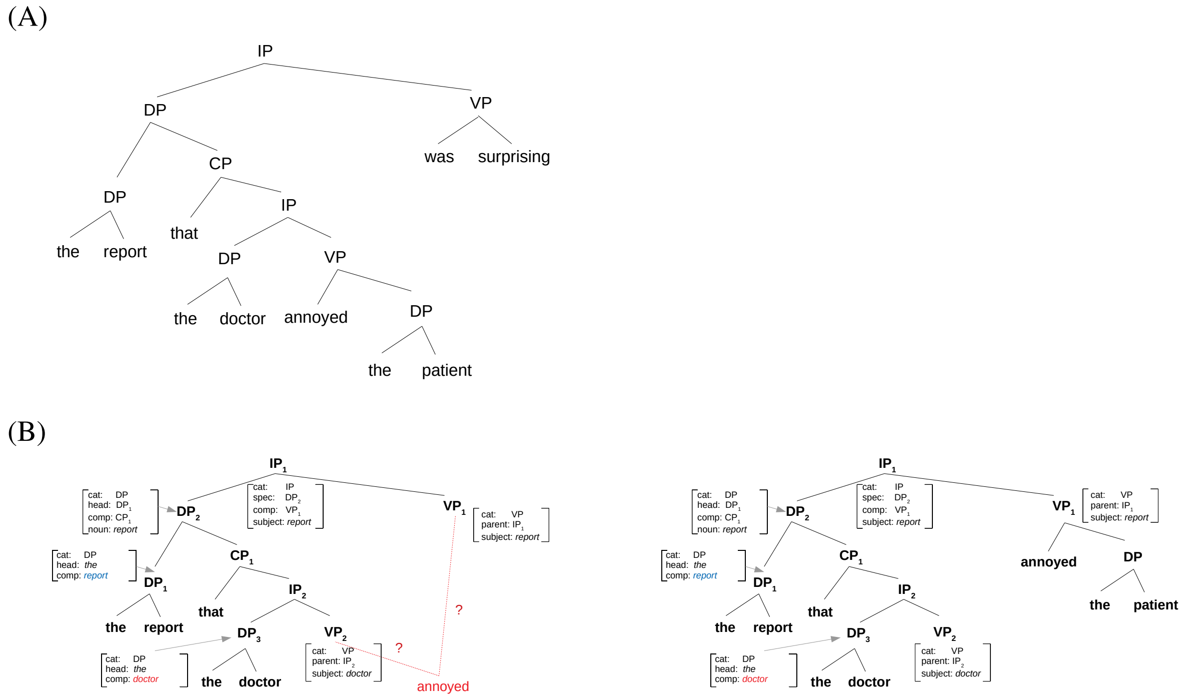

Figure 26: Here, we sketch potential mechanisms by which a retrieval-based left-corner parser such as Lewis and Vasishth [44] (similarly: Rasmussen and Schuler [55], Dotlačil [17]) could be extended to produce the effect of semantic compatibility observed in our experiments. We note that, unlike Resource-Rational Lossy-Context Surprisal, the detailed predictions of such a retrieval-based parser crucially depend on specific assumptions about the grammar and parser rules; we here aim to illustrate what choices would be necessary to implement this effect. (A) Parse tree of a stimulus in the Two condition in the grammar formalism assumed by Lewis and Vasishth [44]. (B) Left: Structure before "annoyed" is observed. Each node in the tree is described by a feature structure stored as a chunk in short-term memory. The current tree has two open predictions that both require a finite verb (at VP_1 and VP_2). Because the model does not represent serial order, it has to rely on other information to decide which of the two nodes to attach to. In this case, the two nodes are distinguished by their subject nouns (report and doctor); here, the model needs to assume that the parser percolates semantic information about the subject through the tree. As "annoyed" is semantically compatible with both subjects, semantic expectations do not rule out (incorrectly) attaching "annoyed" to VP_1. Right: Structure after completing this incorrect attachment. After completing VP_1, the top-level node IP_1 is marked as completed by the parser. Hence, the parser believes the sentence to be complete, leading to disruption when another verb is encountered. While such a mechanism could implement the effect of semantic compatibility, it is less clear how the retrieval-based parser could be extended to account for the effect of embedding bias, as it is not clear how the identity of the first noun impacts the choice of the attachment site for "annoyed". See text for further discussion.

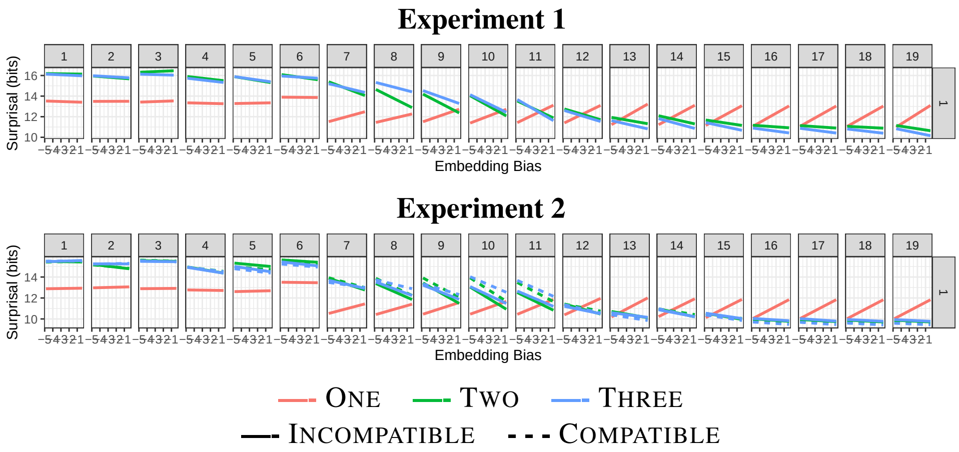

Figure 27: Predictions for an alternative model optimizing for recoverability of the context rather than next-word prediction: Model surprisal on the final verb across values of the retention rate δ. We show linear fits across all item-noun-pairs, averaged across all model runs at a given δ. Compare Figure 4.

8 Comparison with Information-Theoretic Bottleneck

A key property of our resource-rational lossy-context model is a close structural relationship between encodings c' and inputs c. This is different from information-theoretic bottleneck models, which in general do not assume any structural correspondence between the input and the encoding [60].

Here, we illustrate that a purely information-theoretic bottleneck does not predict the empirically observed bias towards surface-similar strings predicted by the resource-rational model. We constructed a very simple and explicit probabilistic grammar that models some key aspects of the One and Two conditions, while being simple enough to permit exact calculations. The grammar is shown in Figure 29; it generates sentences such as “report annoyed patient” and “report that doctor annoyed patient startled janitor”. Crucially, it incorporates a preference for “report+PP” over “report+SC”, and “fact+SC” over “fact+PP”. We note that the grammar is extremely idealizing on purpose; it is intended not as a plausible model of human language, but to qualitatively showcase how certain properties of different models give rise to different predictions.

Using the method of Lagrange multipliers, our resource-rational objective function can be written

$$\mathcal{L}_{\textit{resource-rational}}(\theta) = \mathrm{I}[c_{N+1}|c'_{1\ldots N}] + \kappa \cdot \mathbb{E}\left[\#\{i : c'_i = \text{ERASED}\}\right] \tag{21}$$

where the Lagrange multiplier $\kappa > 0$ depends on δ, and maximizing $\mathcal{L}_{\textit{resource-rational}}(\theta)$ corresponds to solving (3).

A corresponding information-theoretic bottleneck model is obtained by allowing arbitrary encodings c' (without a structural relationship with c), and constraining not the number of retained words, but the mutual information between inputs c and encodings c'. The corresponding objective function thus reads:

$$\mathcal{L}_{\textit{info-bottleneck}}(\theta) = \mathrm{I}[c_{N+1}|c'] - \beta \cdot \mathrm{I}[c_{1\ldots N} : c'] \tag{22}$$

This objective function describes a **past-future** or **predictive information bottleneck** [60, 2, 46, 58, 13, 30, 63]. This describes the problem of compressing observed inputs $c_{1\ldots N}$ into representations c' that maximize informativity about the future, while minimizing the number of encoded bits. The parameter β describes the strength of the pressure towards minimizing the number of encoded bits; it can take values $0 \leq \beta < 1$ ($\beta = 0$ corresponds to perfectly encoding c; $\beta \to 1$ corresponds to preserving no information about c at all).

For each $\beta = 0.1, \ldots, 0.9$, we solved (22) for the grammar given in Figure 29, considering the setting where c' is a discrete random variable with up to 2,000 possible states. For comparison, we also solved

for the resource-rational model Q (3) using the same neural variational method as in our main simulation study; we represented the prior $P(c)$ using a trained LSTM instead of GPT-2.

We then calculated predicted surprisal of the final verb in all sentences of the form "fact *surprised...*" and "fact that doctor annoyed patient *surprised...*" that can be generated by varying nouns and verbs as given by the grammar.

We show the predicted surprisals in Figure 28. The pattern of the resource-rational model agrees qualitatively with the results of the main simulations. When encodings c' are most faithful, at low δ or β, both models shows a positive main effect of embedding bias in the One condition, and no effect in the Two condition. This agrees qualitatively with the conceptual predictions that we derived for Surprisal Theory. In the resource-rational model, there is a range of δ where a negative main effect of embedding buas is observed in the Two condition – this is a signature prediction of models exhibiting a bias towards surface-similar strings. In the information bottleneck model, as β increases ($\beta \geq 0.4$), the effect in the One condition disappears until, for very large β ($\beta \geq 0.7$), the difference between the One and Two condition also disappears. Crucially, there provably is no range of β where embedding bias shows a negative main effect in the Two condition in the Information Bottleneck model.

Fundamentally, the information-theoretic bottleneck method does not show a bias towards surface-similar strings because there is no constraint encouraging similar representations c' for surface-similar strings. For instance, under the information-theoretic bottleneck model, "report that doctor annoyed patient" is mapped to the same code c' as "**fact** that doctor annoyed patient", as these prefixes have exactly the same possible completions under the grammar. In contrast, it is never mapped to the same c' as "report **about** doctor annoyed patient" unless β is very close to 1.

Yet another modeling option could maintain structured representations $c'_{1...N}$ as in our model, but combine these with the information-bottleneck objective (22). Such a model would preferentially delete low-frequency and content words, as this would decrease $\mathrm{I}[c_{1...N} : c']$ the most. We consider such a model implausible, as it would contravene well-established findings about word frequency effects in human memory [29, 26].

We note that, while the presence of a structural relationship between context and memory representations is particularly evident in our model, this is a property shared with previous psycholinguistic model of sentence comprehension. For instance, in models of retrieval-based parsing [44], memory representations consist of chunks, each of which describes some part of the syntactic structure and originates when processing one of the words in the input. The layout of representations is thus tightly linked to the layout of the input, quite distinct from the structure-less compression created by an information bottleneck model. Whereas this might be viewed as an incidental consequence of the use of an incremental parsing algorithm in models such as Lewis and Vasishth [44], our experimental results suggest that such a correspondence between representations and input may in fact be a key aspect of human syntactic processing.

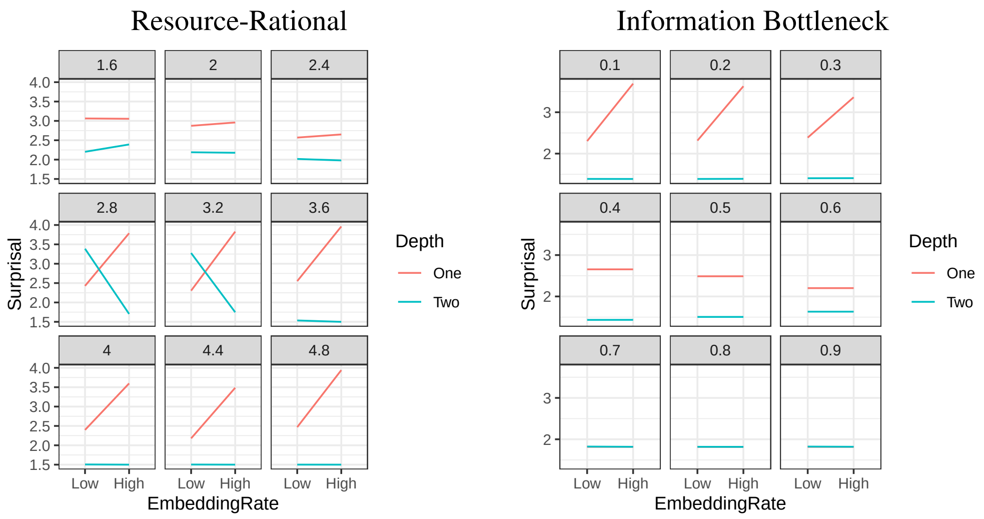

Figure 28: Surprisals on the last verb in the miniature language (Figure 29) under the resource-rational surprisal model as a function of δ (left) and an information-theoretic bottleneck as a function of β (right).

			p	Examples
S	$\rightarrow$	NP_1 VP EOS	0.5	fact annoyed patient.
S	$\rightarrow$	NP_2 VP EOS	0.5	report annoyed patient.
NP_1	$\rightarrow$	N_1	0.1	fact
NP_1	$\rightarrow$	N_1 SC	0.7	fact that doctor annoyed patient
NP_1	$\rightarrow$	N_1 PP	0.2	fact about doctor
NP_2	$\rightarrow$	N_2	0.4	report
NP_2	$\rightarrow$	N_2 SC	0.1	report that doctor annoyed patient
NP_2	$\rightarrow$	N_2 PP	0.5	report about doctor
NP_3	$\rightarrow$	N_3	0.25	doctor
PP	$\rightarrow$	about NP_3	1	about doctor
SC	$\rightarrow$	that NP_3 VP	1	that doctor annoyed patient
VP	$\rightarrow$	V NP_3	1	annoyed doctor
N_1	$\rightarrow$	fact, belief, ...	uniform	fact
N_2	$\rightarrow$	report, story, ...	uniform	report
N_3	$\rightarrow$	doctor, patient, ...	uniform	doctor
V	$\rightarrow$	annoyed, shocked, surprised, pleased, ...	uniform	annoyed

Figure 29: Probabilistic grammar describing a fragment containing the ONE and TWO conditions, restricted to the COMPATIBLE condition. For each of open word classes N_1, N_2, N_3, V, we assumed 10,000 distinct words.

Part IV
Further Analyses

9 Model Fit on Fillers

We also evaluated the model as a predictor of reading times on the fillers. We expected that the model should provide competitive model fit at least in some of the regions of the parameter space in which it also predicts reading times in center embeddings well. Besides our Maze data, we also considered eyetracking and self-paced-reading data collected for these fillers by [61].

To account for spillover effects that are prominent in eyetracking and particularly self-paced reading, we include predictors from the preceding token in all reading measures. We fitted log-transformed reading times for Maze and Self-Paced Reading, and raw reading times for Eye-Tracking[17].

We constructed mixed-effects models with the following fixed effects:

1. Model surprisal, averaged over all model runs at a given retention rate δ[18]

2. word length

3. Log word frequency in the British National Corpus [41]

4. Log word Frequency in the Corpus of Contemporary American English [COCA 15]

5. The same predictors (1-4), and the corresponding reading time measure, for the preceding word

We entered items (i.e., word tokens) and subjects as random effects. Due to computational cost constraints, we created frequentist mixed-effects models using `lme4` [6] with random intercepts only. We compared model fit using Akaike Information Criterion [AIC 1].

There are about 730 distinct tokens in 56 sentences. For Maze, we have data from >700 subjects, each of which read 30 of 56 fillers. For the other modalities, we have considerably less data, both because [61] recruited fewer participants, and because they used multiple lists of fillers, so that not every participant read each of the fillers used in our study.

We show model fit, measured using AIC, in Figure 30. We compare model fit to GPT-2, for which we clipped context length to 20 for fair comparison with the resource-rational model. White cells indicate fit equivalent to vanilla GPT2. Red cells indicate better model fit than vanilla GPT2; blue fit indicates poorer fit. In all three modalities, model fit is at least competitive with vanilla GPT2 across a wide area in the parameter space: In particular, model fit is at least as good as that of GPT2 whenver $\delta \geq 3$. There is also a clear overlap between the regions where model fit is best across the fillers with the regions where prediction is best for the final verb in center embeddings (compare Figure 5). We also specifically compared model fit to Maze reading times between plain surprisal ($\delta = 0$), and the configuration reported in Figure 3 of the main paper ($\delta = 10$). The model strongly improved fit over plain surprisal (ΔAIC = 24).

The success of our model in predicting reading times when retaining only half or less of context words explains recent findings that reading times are impacted by local statistics beyond surprisal [28] and that n-gram models provide relatively good fit to reading times compared to their limited statistical fit to language [64].

[17]The rationale for this is that we use reading times from the preceding word as covariates to account for potential spillover effects. As reading times can be zero in eyetracking, log-transformation does not make sense there.

[18]We found equivalent results when instead computing AIC for every model run individually, and then averaging for all runs at a given retention rate δ.

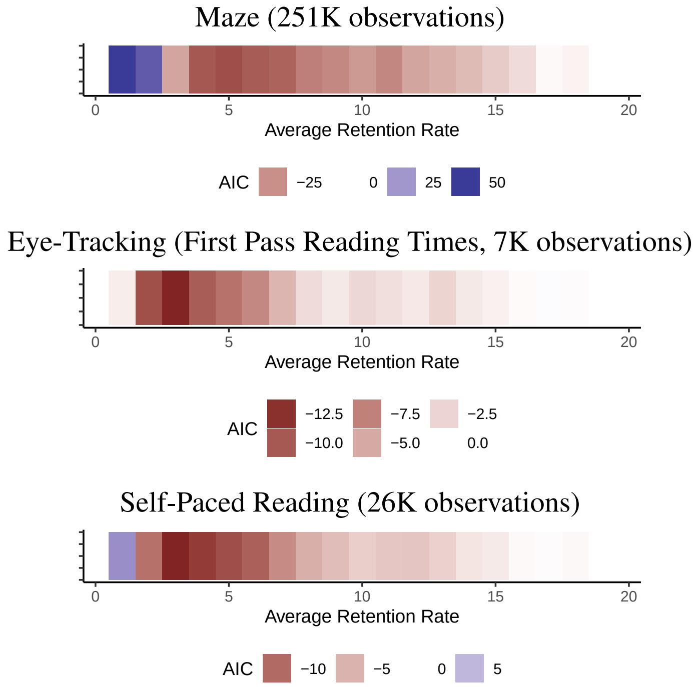

Figure 30: Model fit on reading times on the fillers. Eye-tracking and self-paced reading data was collected by [61]. We show the AIC difference compared to pure GPT2; negative values indicate better model fit. The model improves over GPT2 over a wide range of retention rates. Low retention rates seem to lead to better model fit in eye-tracking and self-paced reading than in Maze, potentially suggesting lower attention in those paradigms.

While the model improves fit in all three modalities, the parameters leading to best fit in Eyetracking and Self-Paced Reading appear to have lower retention rates δ when compared to Maze. This may suggest that participants tend to be less attentive in those paradigms than in Maze.

We considered whether the improvement over full surprisal can be localized to specific contexts or constructions, such as long dependencies. We found no evidence for such localized effects; instead, the improvement appears to be an across-the-board statistical improvement. Investigating this in more detail is an interesting question for future work.

Including DLT We further added DLT integration cost as a predictor into the analyses. We operationalized this as follows based on Demberg and Keller [16]. We parsed the fillers using Stanza [54] into the Universal Dependencies 2 format, a standard representation format for syntactic dependencies. We treated nouns (NOUN and PROPN) and verbs (VERB) as introducing discourse referents.[19] As Demberg and Keller [16] analyzed effects on nouns and verbs separately, we created separate predictors for nouns and verbs. These were centered within the relevant POS category (nouns or verbs), and then set to zero for other words.[20] With the DLT predictors, the model continued to improve over plain surprisal ($\Delta AIC = -23$ for $\delta = 10$ vs full surprisal) in fitting the Maze data.

[19] We did not treat AUX as introducing a discourse referent because Demberg and Keller [16] found a *facilitating* effect of intervening auxiliaries, opposite to the DLT prediction if they are treated as discourse references.

[20] We found equivalent results when zeroing them for other words *before* centering.

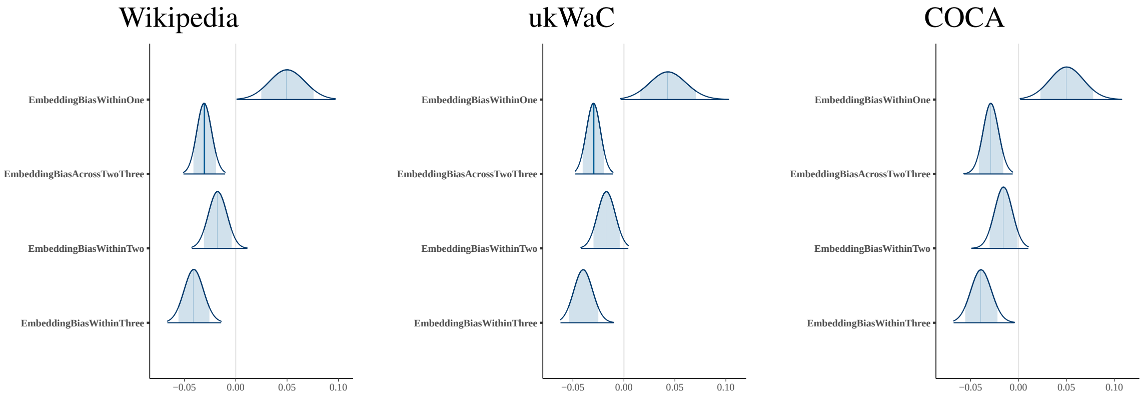

Figure 31: Posterior of the effect of Embedding Bias on reading times on the final verb (on the data from Section 6.6), estimating Embedding Bias using three large-scale English corpora. The posteriors are obtained from the posterior of the mixed-effects analysis of log-transformed reading times fitted separately using three versions of Embedding Bias; for instance, the effect within ONE is obtained as $\beta_{EmbeddingBias} - 0.9\beta_{EmbeddingBias:Embedding}$ (cf. Section 3.4). All corpora lead to similar estimated coefficients in the different conditions. This confirms the robustness of the finding to the corpus used for estimating Embedding Bias.

10 Comparing Embedding Bias with other Possible Predictors

10.1 Estimating Embedding Bias Using Other Corpora

In Figures 31–32, we show that the effect of Embedding Bias found in Experiments 1–3 is robust to different corpora used for estimating Embedding Bias: In addition to Wikipedia, we also considered COCA [15, 1B words of American English] and ukWaC [19, 2B words primarily of British English]. Results are very similar across the three corpora. [21]

10.2 Embedding Bias and other Predictors

The effect of Embedding Bias consistently observed across experiments in reading times, production, and ratings might be among the more surprising experimental findings in this paper. In the model, it arises from the differences in posterior probability assigned to the correct number of embedding levels. Given that this is a new effect not previously reported in the psycholinguistic literature, we asked whether there might be other factors correlated with Embedding Bias that might also capture the observed behavioral data.

We considered the following properties of nouns:

1. EMBEDDINGBIAS, i.e.

$$\log P(that|the\ NOUN) \tag{23}$$

   estimated on the corpora discussed in Section 10.1 (Wikipedia, ukWaC, COCA).

   Note that EMBEDDINGBIAS is the difference of (2) and (3) listed below.

[21] In the production study, estimated effects are stronger with ukWaC and COCA counts than with the Wikipedia counts reported in the main paper; this is in part because the word *inkling* is much more common in those corpora than in Wikipedia, leading to different and potentially more reliable estimates of Embedding Bias. We did not include *inkling* in model simulations due to the low number of occurrences in Wikipedia.

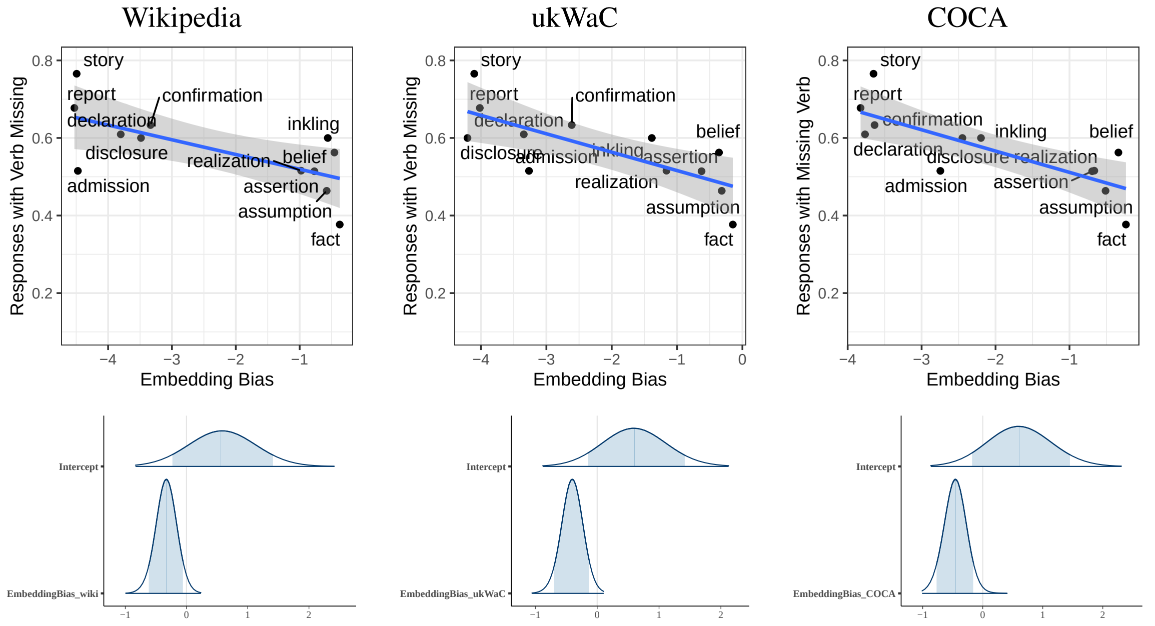

$\beta = -0.33$, 95% CrI $[-0.61, -0.06]$ $P(\beta > 0) = 0.01025$

$\beta = -0.40$, 95% CrI $[-0.70, -0.13]$ $P(\beta > 0) = 0.00275$

$\beta = -0.46$, 95% CrI $[-0.77, -0.16]$ $P(\beta > 0) = 0.00375$

Figure 32: Results of the English production study when estimating Embedding Bias using Wikipedia (as reported in the main paper) and the two other large corpora described in Section 10.1. Top: By-noun rates of incomplete responses. Bottom: Results of mixed-effects analysis (compare Figure 15). A significant effect of similar effect size is found with all three corpora.

2. MARGINALPROBABILITY:

$$\log P(the\ NOUN) \tag{24}$$

3. JOINTPROBABILITY:

$$\log P(the\ NOUN\ that) \tag{25}$$

4. COMPLEMENTCLAUSEBIAS: the log-probability that a noun, when it is followed by *that* embeds a complement clause rather than a relative clause:

$$\log P(SC|the\ NOUN\ that) \tag{26}$$

This probability is referred to as *inclusive CC noun bias* by Staub et al. [57].

Estimating this term requires syntactic analysis distinguishing SCs from other constructions (e.g., relative clauses). We found that automatic parsing was not fully reliable in distinguishing these; thus, we followed Staub et al. [57] in using manual annotation. While they used search engine results, we found those (as obtained in 2021) strongly biased towards names and website titles. We thus instead sampled occurrences of "the NOUN that" from the English Wikipedia (the same corpus used to estimate the other probabilities). A trained linguist annotated these for (1) SC, (2) RC ("The fact that was mentioned..."), (3) Other ("The part of the report that was written first...") readings. Annotation continued until either a Binomial confidence interval for $logP(SC|\text{the NOUN that})$ had width ≤ 0.15, or no further data was availbale. We conducted annotation for 95 nouns from the initial pool (see Section 11), a superset of the nouns considered in the experiments and simulations.

For all relevant nouns, this condition was reached after annotating less than 200 samples. Results are deposited in the project repository.[22].

Across the 95 nouns, we found that $P(SC|\text{the NOUN that})$ was generally close to 1 (mean 0.88; median 0.97; minimum 0.2 for 'relief' and 'bet'; these are not used in the experiments).

5. SUBJECTBIAS: the log-probability that a noun is a subject. Here, we found automatic parsing to be sufficiently reliable. We parsed about 20% of the English Wikipedia using Stanza [54] and recorded the fraction of times a noun's dependency label was *nsubj* or a subtype.

   We reasoned that, under accounts of center embeddings in theories of cue-based retrieval (see Section 7), SUBJECTBIAS rather than EMBEDDINGBIAS could determine how likely humans might be to (erroneously) attach a noun such as *report* as the subject of *annoyed* in *The report that the doctor annoyed the patient...*, causing difficulty on the final verb.

6. WORDLENGTH measured in characters

7. ENTAILING: entailing/non-entailing contrast, see Section 11

8. HOMOPHONEVERB: homophony with a verb (e.g., remark, report)

9. DEVERBAL: derivational relationship with a verb, including homophony (e.g., information)

We first fitted, for each of these predictors, a frequentist mixed-effects model with lme4 [6] (using ML, not REML), and computed its log-likelihood. These models were fitted on the subset in the TWO/THREE conditions of the data used in Section 6.6, and included TWO/THREE contrast and the COMPATIBILITY contrast, their interaction, and the additional predictor of interest. Due to convergence issues, the model included only random intercepts. The log-likelihood values for each predictor are shown in Figure 33. EMBEDDINGBIAS estimated from the three corpora result in the best model fits (lowest negative log-likelihood), with some distance to the other predictors. Interestingly, the ukWaC version provided the best model fit; this might be either due to genre differences or the presence of British English speakers among the participant population on Prolific.

We further used forward model selection with BIC to determine whether better fit can be obtained by combining different predictors. This was not the case, as the best model overall included only EMBEDDINGBIAS.

In conclusion, Embedding Bias is a much better predictor of noun-specific reading times on the final verb in the TWO/THREE condition than the other predictors investigated. There is also little evidence that adding any further predictor to it would improve model fit.

As a further way of understanding how Embedding Bias predicts reading times, we conducted the following procedure. We fitted a version of the mixed-effects model with all fixed and random effects involving Embedding Bias removed. This means that any by-noun variation is now encoded in the fitted random effects. For each noun, its effect on reading times in the TWO and THREE conditions is given as its random intercept plus 0.1 times its random slope for the EMBEDDED fixed effect. This provides a measure of the noun's impact on reading times, without any bias towards an effect of Embedding Bias or any property of nouns. In Figure 34, we plot the posterior means for these effects against Embedding Bias. We found a strong correlation between the two measures ($R = -0.72$, $p = 1.5 \cdot 10^{-7}$; $\rho = -0.70$, $p = 1.3 \cdot 10^{-6}$). [23]

[22] `https://gitlab.com/m-hahn/resource-rational-surprisal/-/blob/main/materials/nouns/corpus_counts/wikipedia/RC_annotate/results/collectResults.py.tsv`

[23] We also computed the correlation across different posterior samples, instead of for the mean. We obtained a mean correlation of $R = -0.59$, 95% CrI $[-0.72, -0.45]$.

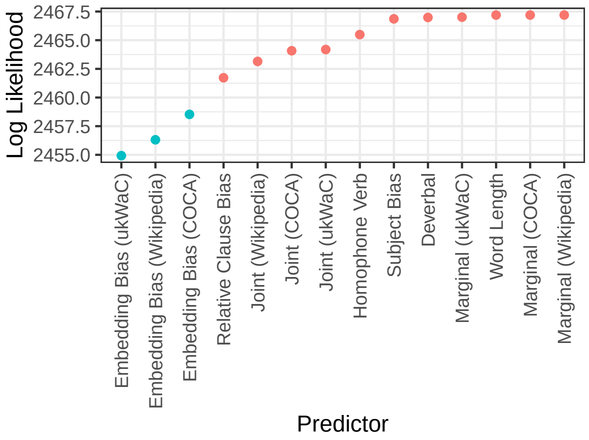

Figure 33: Negative log-likelihoods (lower is better) of models of reading times with various properties of nouns added. The best model fit is achieved by Embedding Bias (blue), as estimated on three large corpora of English.

In conclusion, Embedding Bias is not just a better predictor of reading times on the final verb than other properties of nouns, but also accounts for a substantial amount of variance in the by-nouns effect on reading times.

An interesting connection can be made to work suggesting a role of mutual information in language and human language processing [e.g. 14]: Up to a constant, Embedding Bias is equal to the mutual information between the noun and *that*. In this sense, anticipating the final verb is easier when the mutual information between the noun and the crucial functional element (the word *that*) is higher.

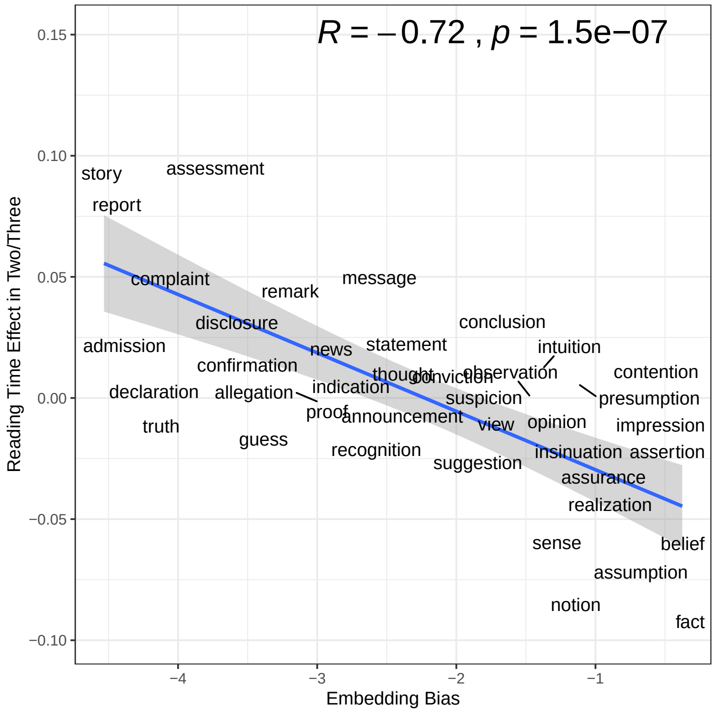

Figure 34: Comparing relative reading time effects of nouns in the TWO/THREE conditions with Embedding Bias as estimated from Wikipedia. Reading time effects were estimated using a mixed-effects analysis of reading times on the critical verb, but in which fixed effects including embedding bias were removed (see text for details). A strong correlation between the two confirms that reading time differences on the critical verb closely track embedding bias.

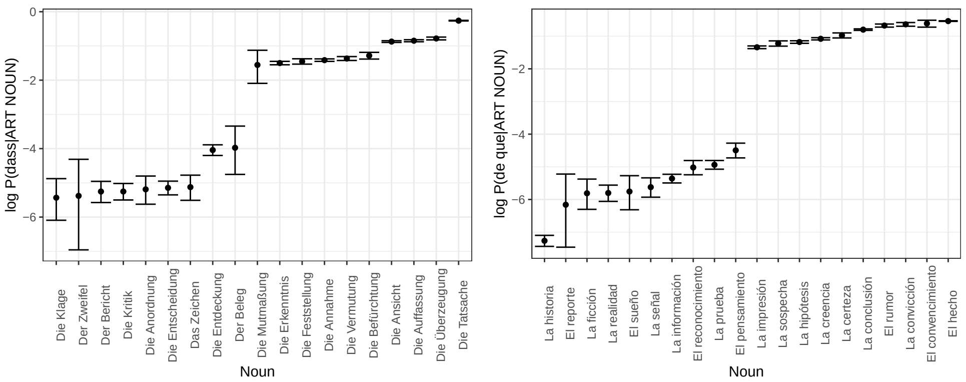

Figure 35: Nouns in German and Spanish selected for Experiment 3, with Embedding Bias and a 95% binomial confidence interval.

Part V
Materials

11 Nouns and Embedding Bias

We collected nouns taking sentential complements from the Penn Treebank (English) and the Universal Dependencies treebanks (German and Spanish).

English From an initial set of 161 English nouns labeled as embedding a complement clause in the Penn Treebank, we selected 58 nouns based on overall semantic fit with the items. While we used all 58 nouns in model simulations, the behavioral experiments focused on nouns with very low and very high embedding bias to maximize statistical precision.

We estimated embedding bias using Wikipedia; see Section 10.1 for results using two other large corpora for estimating Embedding Bias. Note that, in English, *that* can embed both complement clauses and relative clauses; Embedding Bias does not distinguish between those different types of embedded clauses. We also evaluated nouns' biases specifically towards complement clauses in Section 10, finding that the nouns under consideration tend to be strongly biased towards complement clause embedding as opposed to relative clause embedding. In that section, we also investigated whether separately considering a noun's bias to embed complement or relative clauses predicts human reading times beyond Embedding Bias, finding little, if any, evidence for such effects.

Semantic Grouping In order to eliminate any item-specific confounds, we cross nouns with items across subjects in the reading time studies. This raises the question of how to deal with combinations that are semantically infelicitous, which primarily happens with nouns that presuppose truth of the embedded proposition – e.g., the combination "fact" + "was false" tends to be semantically infelicitous[24]

We therefore grouped nouns into three classes and annotated the items for compatibility with those, matching only compatible pairs. We distinguished entailing and non-entailing nouns, as defined by [37]. We further distinguished *claim*-like and *accusation*-like nouns to account for a group of items that

[24] We note that such combinations typically still are felicitous in certain contexts, e.g. *Everyone else tries to guess whether they think the **fact was false***. `https://medium.com/future-of-design-in-higher-education/zoom-friendly-warmups-and-icebreakers-3400c8b7263`; *If they only discovered that the **fact was a lie** after the wedding* `https://www.catebrough.com/can-i-have-my-south-carolina-marriage-annulled/`.

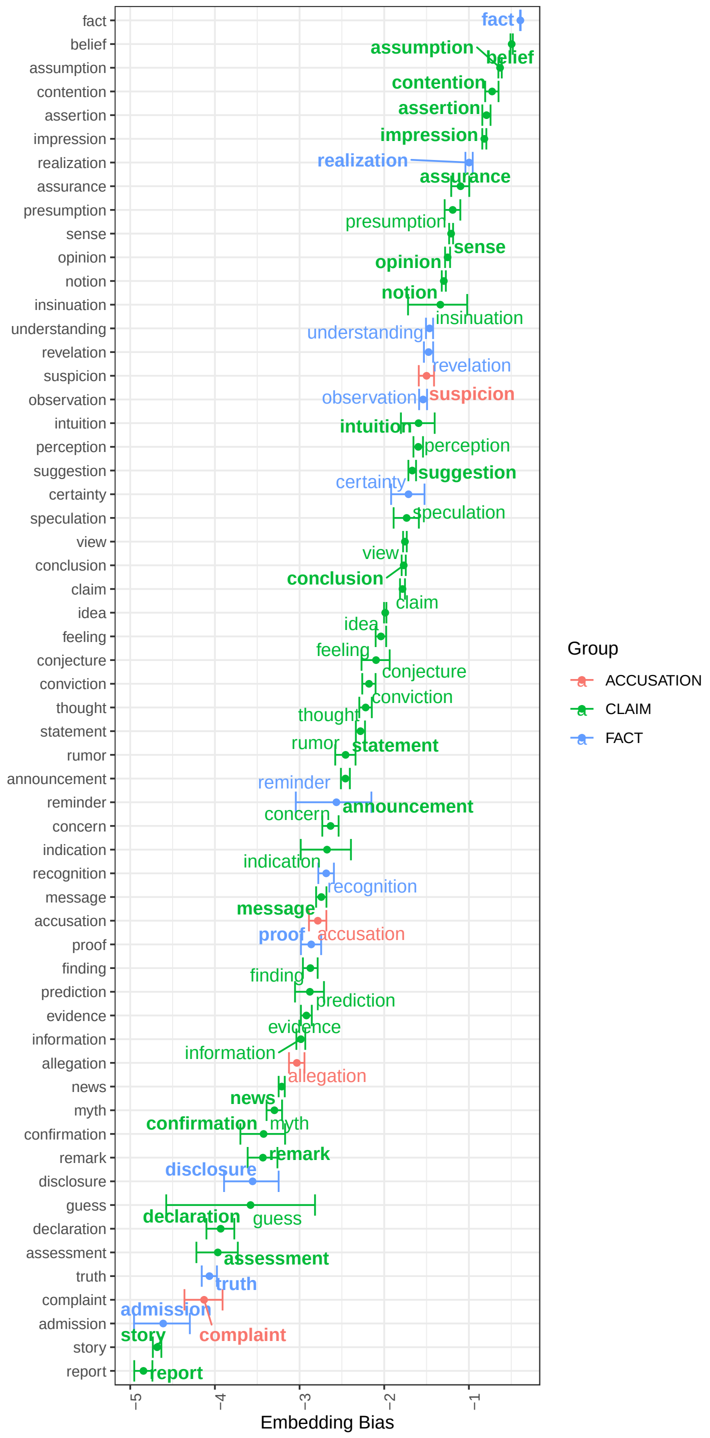

Figure 36: English nouns by Embedding Bias as estimated on Wikipedia, annotated for the three semantic classes. This plot contains all 58 nouns considered in model simulations. Nouns used in Experiments 1–2 are marked in bold. We show 95% binomial confidence intervals for embedding bias.

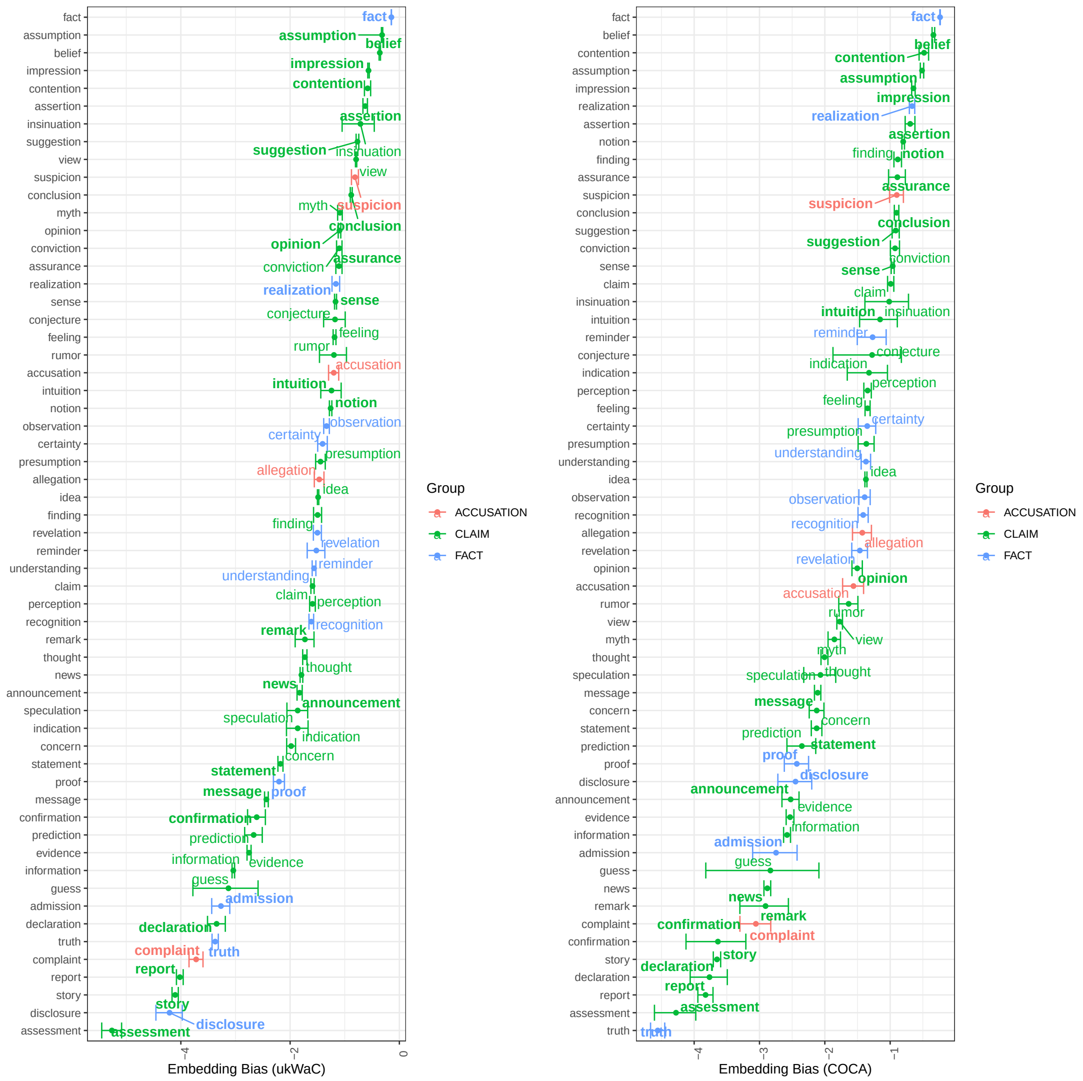

Figure 37: English nouns by Embedding Bias as estimated on ukWaC and COCA (see Section 10.1). Compare Figure 36.

presuppose positive valence (e.g., “the NOUN that the paramedic saved the children...”), making them less compatible with *accusation*-like nouns. This leads to the following three classes.

1. Entailing nouns (e.g., fact).
2. Claim-like non-entailing nouns (e.g., claim, report).
3. Accusation-like non-entailing nouns (e.g., accusation, allegation).

See Figure 36 for the full list of nouns used in simulations and studies with this annotation.

Note that we on purpose did not aim to only include high-plausibility combinations. Rather, our goal was to eliminate semantic anomalies where an entailment presupposition is violated. While it would be possible to consider the *plausibility* or *frequency* of a noun-verb match, this would introduce additional probabilistic biases into the noisy-channel reasoning that could confound the effect of theoretical interest, i.e., the Embedding Bias effect.

German and Spanish In German and Spanish, we selected 20 nouns based both on semantic fit with the nouns used in the English experiments, and focused on extreme values of complement clause bias to maximize power in the production studies. Resulting nouns are shown in Figure 35.

We note that, in the Spanish study, due to a scripting error not discovered before data analysis, the noun *pensamiento* was not included in the experiment, resulting in only 19 nouns.

Whereas “the NOUN that” allows both sentential complement and relative clause continuations in English, German and Spanish use complementizers (*dass* and *de que*) that are used only for complement clauses, not used for relative clauses.

12 Stimuli (Reading Time Studies)

The stimuli were constructed subject to the following constraints:

1. The critical verb and the second-to-last verb phrase were neither identical, strongly similar (e.g. annoyed and startled), nor were they differentially semantically similar in the COMPATIBLE and INCOMPATIBLE conditions. For instance, if the inner verb is “annoyed” in the COMPATIBLE condition, the final verb cannot be “startled”.

2. In Experiment 2, the last word in the second-to-last verb phrase was identical across the COMPATIBLE and INCOMPATIBLE conditions. While the Maze paradigm largely avoids spillover effects, this constraint helps remove any remaining doubt that spillover could impact the comparison between COMPATIBLE and INCOMPATIBLE.

3. In Experiment 2, the overall distribution of COCA log word frequency of the second-to-last verb, across the items, was matched in the COMPATIBLE and INCOMPATIBLE conditions. This was done to avoid any possibility of increased difficulty due to rare lexical items spilling over to the final verb.

These constraints had not been consistently enforced in the preceding experiments (Section 6). While they rigorously rule out various potential confounds, we did not observe evidence that these constraints had an impact on the effects of interest in either the model predictions or human reading times.

12.1 Stimuli (Experiment 1)

For each item, we show the full version in the THREE condition. The other conditions are obtained by removing one or both embedded clauses. For each item, we also indicate the semantic groups of nouns that we paired it with (see Section 11).

1. The NOUN [that the commander [who the president appointed] was fired] annoyed people. (claim, fact)

2. The NOUN [that the criminal [who the officer arrested] was innocent] turned out to be entirely bogus. (claim)

3. The NOUN [that the surgeon [who the nurse assisted] read the news] was not a secret. (claim, fact)

4. The NOUN [that the worker [who the thug attacked] was rescued] surprised people. (claim, fact)

5. The NOUN [that the pianist [who the sponsors backed] played sonatas] pleased everyone. (claim, fact)

6. The NOUN [that the actor [who the producer blamed] got very sick] was sad to hear. (fact, claim)

7. The NOUN [that the politician [who the banker bribed] laundered money] came as a shock to his supporters. (fact, claim, accusation)

8. The NOUN [that the paramedic [who the swimmer called] saved the children] was good news. (claim, fact)

9. The NOUN [that the trader [who the businessman consulted] tricked Kim] did not bother Mary. (claim, fact, accusation)

10. The NOUN [that the mayor [who the farmer disliked] was elected] did not bother the farmer. (claim, fact)

11. The NOUN [that the neighbor [who the woman distrusted] poisoned the dog] seemed to be a malicious smear. (claim, accusation)
12. The NOUN [that the senator [who the diplomat encountered] opposed Josh] was absolutely true. (claim)
13. The NOUN [that the preacher [who the parishioners fired] was lying] startled Anne. (claim, fact, accusation)
14. The NOUN [that the counselor [who the writer hired] hated the janitor] was dismissed as untrue. (claim)
15. The NOUN [that the singer [who the fans idolized] won a prize] was exciting. (claim, fact)
16. The NOUN [that the CEO [who the employee impressed] teased everyone] drove Bill crazy. (claim, fact, accusation)
17. The NOUN [that the sculptor [who the painter invited] made sculptures] did not surprise anyone. (claim, fact)
18. The NOUN [that the principal [who the teacher liked] turned everyone down] surprised the parents. (claim, fact)
19. The NOUN [that the dancer [who the audience loved] was interviewed on TV] seemed exciting. (claim, fact)
20. The NOUN [that the entrepreneur [who the trickster misled] was admired everywhere] gave Mary fits. (claim, fact)
21. The NOUN [that the engineer [who the tenant phoned] repaired the TV] was very believable. (claim)
22. The NOUN [that the trickster [who the witness recognized] was arrested] calmed people down. (claim, fact)
23. The NOUN [that the scientist [who the mayor relied on] had faked data] made Sue angry. (claim, accusation)
24. The NOUN [that the bureaucrat [who the guard shouted at] instructed the newscaster] was bogus. (claim)
25. The NOUN [that the employee [who the manager supervised] was reprimanded] devastated his family. (claim, fact)
26. The NOUN [that the president [who the commander supported] was impeached] shocked the people. (claim, fact)
27. The NOUN [that the driver [who the tourist thanked] was ill] bothered Bill. (claim, fact)
28. The NOUN [that the thief [who the detective tracked] robbed everyone] was disconcerting. (claim, fact, accusation)
29. The NOUN [that the athlete [who the coach trained] expressed anger] came as a disappointment. (fact, claim, accusation)

30. The NOUN [that the runner [who the psychiatrist treated] won the marathon] seemed very interesting. (claim, fact)
31. The NOUN [that the doctor [who the patient visited] was talented] impressed the whole city. (claim, fact)
32. The NOUN [that the pediatrician [who the receptionist worked for] mistrusted the parent] had troubled people. (claim, fact)

12.2 Stimuli (Experiment 2)

Formatting is as in Section 12.1. For the second-to-last verb phrase, the show the INCOMPATIBLE and COMPATIBLE version, separated by a slash.

1. The NOUN [that the teacher [who the principal liked] [failed the student/annoyed the student]] was only a malicious smear. (claim, accusation)
2. The NOUN [that the doctor [who the colleague mistrusted] [cured the patients/bothered the patients]] seemed hard to believe. (claim, fact)
3. The NOUN [that the president [who the farmer admired] [appointed the commander/impressed the commander]] was entirely bogus. (claim)
4. The NOUN [that the victim [who the swimmer rescued] [sued the criminal/surprised the criminal]] appeared on TV. (claim, fact)
5. The NOUN [that the guest [who the cousin invited] [visited the uncle/pleased the uncle]] drove Jill crazy. (claim, fact)
6. The NOUN [that the psychiatrist [who the nurse assisted] [diagnosed the patient/horrified the patient]] became widely known. (claim, fact)
7. The NOUN [that the driver [who the guide called] [phoned the tourist/amazed the tourist]] was absolutely true. (claim)
8. The NOUN [that the actor [who the fans loved] [greeted the director/astonished the director]] appeared to be true. (claim)
9. The NOUN [that the banker [who the analyst defrauded] [trusted the customer/excited the customer]] proved to be made up. (claim)
10. The NOUN [that the judge [who the attorney hated] [acquitted the defendant/vindicated the defendant]] was a lie. (claim)
11. The NOUN [that the captain [who the crew trusted] [commanded the sailor/motivated the sailor]] was nice to hear. (claim, fact)
12. The NOUN [that the manager [who the boss authorized] [hired the intern/saddened the intern]] seemed absurd. (claim, fact)
13. The NOUN [that the bully [who the children hated] [harassed the boy/shocked the boy]] was entirely correct. (claim, fact, accusation)
14. The NOUN [that the plaintiff [who the jury interrogated] [interrupted the witness/startled the witness]] made it into the news. (claim, fact)

15. The NOUN [that the drunkard [who the thug hit] [outwitted the bartender/stunned the bartender]] sounded hilarious. (claim, fact)
16. The NOUN [that the medic [who the survivor thanked] [greeted the surgeon/surprised the surgeon]] turned out to be untrue. (claim)
17. The NOUN [that the lifesaver [who the soldier taught] [rescued the swimmer/encouraged the swimmer]] took the townspeople by surprise. (claim, fact)
18. The NOUN [that the fisherman [who the gardener helped] [admired the politician/delighted the politician]] was interesting. (claim, fact)
19. The NOUN [that the janitor [who the organizer criticized] [ignored the audience/amused the audience]] was funny. (claim, fact)
20. The NOUN [that the investor [who the scientist detested] [deceived the entrepreneur/disappointed the entrepreneur]] drove everyone crazy. (claim, fact)
21. The NOUN [that the firefighter [who the neighbor insulted] [rescued the houseowner/discouraged the houseowner]] went unnoticed. (claim, fact)
22. The NOUN [that the agent [who the FBI sent] [arrested the criminal/confused the criminal]] was acknowledged. (fact, claim)
23. The NOUN [that the plumber [who the apprentice consulted] [assisted the woman/puzzled the woman]] was true. (claim)
24. The NOUN [that the senator [who the diplomat supported] [defeated the opponent/troubled the opponent]] deserved attention. (fact, claim)
25. The NOUN [that the fiancé [who the author met] [married the bride/startled the bride]] did not surprise anyone. (fact, claim)
26. The NOUN [that the businessman [who the sponsor backed] [fired the employee/surprised the employee]] came as a disappointment. (fact, claim, accusation)
27. The NOUN [that the thief [who the detective caught] [robbed the woman/enraged the woman]] broke her family's heart. (fact, claim, accusation)
28. The NOUN [that the criminal [who the stranger distracted] [abducted the officer/baffled the officer]] seemed concerning. (fact, claim, accusation)
29. The NOUN [that the customer [who the vendor welcomed] [contacted the clerk/terrified the clerk]] was very believable. (claim)
30. The NOUN [that the commander [who the president appointed] [was fired yesterday/was confirmed yesterday]] troubled people. (claim, fact)
31. The NOUN [that the trickster [who the woman recognized] [was arrested by the police/was acknowledged by the police]] calmed people down. (claim, fact)
32. The NOUN [that the politician [who the farmer trusted] [was elected three days ago/was refuted three days ago]] did not bother the farmer. (claim, fact)
33. The NOUN [that the politician [who the banker bribed] [seemed corrupt to everyone/seemed credible to everyone]] gave John the chills. (claim, fact)

34. The NOUN [that the sculptor [who the painter admired] [made sculptures in the US/made headlines in the US]] did not surprise anyone. (claim, fact)
35. The NOUN [that the runner [who the psychiatrist treated] [won the marathon in France/was widely known in France]] turned out to be incorrect. (claim)
36. The NOUN [that the analyst [who the banker trusted] [repaired the TV this morning/appeared on TV this morning]] was very believable. (claim)
37. The NOUN [that the child [who the medic rescued] [wrote articles in newspapers/was mentioned in newspapers]] seemed very interesting. (claim, fact)
38. The NOUN [that the carpenter [who the craftsman carried] [hurt the apprentice/confused the apprentice]] was acknowledged. (claim, fact, accusation)
39. The NOUN [that the daughter [who the sister found] [greeted the grandmother/frightened the grandmother]] seemed concerning. (claim, fact, accusation)
40. The NOUN [that the tenant [who the foreman looked for] [questioned the shepherd/annoyed the shepherd]] proved to be made up. (claim, accusation)
41. The NOUN [that the pharmacist [who the stranger saw] [questioned the customer/distracted the customer]] sounded surprising. (claim, fact)
42. The NOUN [that the surgeon [who the patient thanked] [cured his colleagues/shocked his colleagues]] was ridiculous. (claim)

13 Stimuli (Production Study)

13.1 English

1. The NOUN that the the school principal who the teacher
2. The NOUN that the the famous sculptor who the painter
3. The NOUN that the the marketing whiz who the artist
4. The NOUN that the the marathon runner who the psychiatrist
5. The NOUN that the the frightened child who the medic
6. The NOUN that the the alleged criminal who the officer
7. The NOUN that the the college student who the professor
8. The NOUN that the the suspected mobster who the media
9. The NOUN that the the leading man who the starlet
10. The NOUN that the the old preacher who the parishioners
11. The NOUN that the the young violinist who the sponsors
12. The NOUN that the the conservative senator who the diplomat
13. The NOUN that the the military commander who the president
14. The NOUN that the the injured victims who the criminal
15. The NOUN that the the savvy politician who the banker
16. The NOUN that the the famous surgeon who the patient
17. The NOUN that the the violent extremist who the agent
18. The NOUN that the the office clerk who the customer
19. The NOUN that the the shady trader who the businessman
20. The NOUN that the the successful CEO who the employee
21. The NOUN that the the taxi driver who the tourist
22. The NOUN that the the angry bookseller who the thief
23. The NOUN that the the odd neighbor who the woman
24. The NOUN that the the mad scientist who the mayor
25. The NOUN that the the ambitious student who the bully
26. The NOUN that the the cunning trickster who the woman
27. The NOUN that the the aspiring entrepreneur who the philanthropist
28. The NOUN that the the courageous lifesaver who the swimmer

13.2 German

1. DET NOUN dass der Schulleiter, den der Lehrer, den der Bäcker
2. DET NOUN dass der Bildhauer, den die Anwältin, die die Richterin
3. DET NOUN dass der Marketingspezialist, den die Künstlerin, die die Malerin
4. DET NOUN dass der Marathonläufer, den der Psychiater, den die Patientin
5. DET NOUN dass das Kind, das der Sanitäter, den das Opfer
6. DET NOUN dass der Kriminelle, den der Polizist, den der Spion
7. DET NOUN dass der Student, den der Professor, den der Kollege
8. DET NOUN dass der Mafioso, den der Journalist, den die Informatikerin
9. DET NOUN dass der Schauspieler, den der Star, den der Feuerwehrmann
10. DET NOUN dass der Pfarrer, den das Gemeindemitglied, die der Gläubige
11. DET NOUN dass der Geigenspieler, den die Sponsoren, die der Tierarzt
12. DET NOUN dass der Abgeordnete, den die Diplomatin, den die Botschafterin
13. DET NOUN dass der Kommandant, den der Präsident, den der Minister
14. DET NOUN dass das Opfer, das der Verbrecher, den der Gefangene
15. DET NOUN dass der Politiker, den der Banker, den der Investor
16. DET NOUN dass der Chirurg, den der Patient, den der Zahnarzt
17. DET NOUN dass der Extremist, den der Agent, den der Polizist
18. DET NOUN dass der Büroangestellte, den der Kunde, den der Gast
19. DET NOUN dass der Händler, den der Unternehmer, den der Verkäufer
20. DET NOUN dass der Chef, den der Angestellte, den der Praktikant
21. DET NOUN dass der Taxifahrer, den der Tourist, den der Kellner
22. DET NOUN dass der Buchhändler, den der Dieb, den der Kriminelle
23. DET NOUN dass der Nachbar, den der Mann, den der Handwerker
24. DET NOUN dass der Wissenschaftler, den der Bürgermeister, den der Gemeinderat
25. DET NOUN dass der Junge, den der Clown, den der Schulleiter
26. DET NOUN dass der Betrüger, den die Frau, die der Metzger
27. DET NOUN dass der Unternehmer, den der Wohltäter, den der Millionär
28. DET NOUN dass der Lebensretter, den der Schwimmer, den der Tourist

Matrix clauses:

1. Lisa hat gesagt, dass
2. Klaus hat erzählt, dass
3. Ich habe gehört, dass
4. Hans hat behauptet, dass
5. Luigi hat gedacht, dass
6. Ibrahim hat gemeint, dass
7. Die Leute glauben, dass
8. Ich denke nicht, dass
9. Niemand denkt wirklich, dass
10. Vorher erfuhr Angela, dass
11. Peter sagte mir, dass
12. Gestern sagte Norbert, dass

13.3 Spanish

Prefixes:

1. DET NOUN de que el director que
2. DET NOUN de que el escultor que
3. DET NOUN de que el vendedor que
4. DET NOUN de que el corredor que
5. DET NOUN de que el niño que
6. DET NOUN de que el criminal que
7. DET NOUN de que el estudiante que
8. DET NOUN de que el mafioso que
9. DET NOUN de que el actor que
10. DET NOUN de que el predicador que
11. DET NOUN de que el violinista que
12. DET NOUN de que el senador que
13. DET NOUN de que el comandante que
14. DET NOUN de que la víctima que
15. DET NOUN de que el político que
16. DET NOUN de que el cirujano que

17. DET NOUN de que el extremista que
18. DET NOUN de que el empleado que
19. DET NOUN de que el empresario que
20. DET NOUN de que el CEO que
21. DET NOUN de que el taxista que
22. DET NOUN de que el librero que
23. DET NOUN de que el vecino que
24. DET NOUN de que el científico que
25. DET NOUN de que el alumno que
26. DET NOUN de que el embaucador que
27. DET NOUN de que el jefe que
28. DET NOUN de que el salvavidas que

References

[1] H. Akaike. Information theory and an extension of the maximum likelihood principle. In B. Petrov and F. Csaki, editors, *International Symposium on Information Theory*, 1973.

[2] N. Amir, S. Tiomkin, and N. Tishby. Past-future information bottleneck for linear feedback systems. *2015 54th IEEE Conference on Decision and Control (CDC)*, pages 5737–5742, 2015.

[3] J. R. Anderson. *The Adaptive Character of Thought*. Erlbaum, Hillsdale, NJ, 1990.

[4] J. Barnard, R. McCulloch, and X. L. Meng. Modeling covariance matrices in terms of standard deviations and correlations, with application to shrinkage. *Statistica Sinica*, 10(4):1281–1311, 2000.

[5] D. Barr, R. Levy, C. Scheepers, and H. J. Tily. Random effects structure for confirmatory hypothesis testing: Keep it maximal. *Journal of memory and language*, 68 3, 2013.

[6] D. Bates, M. Machler, B. Bolker, and S. C. Walker. Fitting linear mixed-effects models using lme4. *Journal of Statistical Software*, 67:1–48, 2014.

[7] V. Boyce, R. Futrell, and R. P. Levy. Maze made easy: Better and easier measurement of incremental processing difficulty. *Journal of Memory and Language*, 111:104082, 2020.

[8] S. P. Boyd and L. Vandenberghe. *Convex Optimization*. 2006.

[9] P.-C. Bürkner. brms: An R package for bayesian multilevel models using Stan. *Journal of Statistical Software, Articles*, 80(1):1–28, 2017.

[10] B. Carpenter, A. Gelman, M. D. Hoffman, D. Lee, B. Goodrich, M. Betancourt, M. A. Brubaker, J. Guo, P. Li, and A. Riddell. Stan: A probabilistic programming language. *Journal of Statistical Software*, 76(1):1–32, 2017.

[11] M. H. Christiansen and M. MacDonald. A usage-based approach to recursion in sentence processing. *Language Learning*, 59:126–161, 2009.

[12] T. M. Cover and J. A. Thomas. Elements of information theory. 2005.

[13] F. Creutzig, A. Globerson, and N. Tishby. Past-future information bottleneck in dynamical systems. *Physical review. E, Statistical, nonlinear, and soft matter physics*, 79 4 Pt 1:041925, 2009.

[14] J. Culbertson, M. Schouwstra, and S. Kirby. From the world to word order: Deriving biases in noun phrase order from statistical properties of the world. *Language*, 2020.

[15] M. Davies. The Corpus of Contemporary American English (COCA). 2012.

[16] V. Demberg and F. Keller. Data from eye-tracking corpora as evidence for theories of syntactic processing complexity. *Cognition*, 109:193–210, 2008.

[17] J. Dotlačil. Parsing as a cue-based retrieval model. *Cognitive Science*, 45(8):e13020, 2021. doi: https://doi.org/10.1111/cogs.13020. URL `https://onlinelibrary.wiley.com/doi/abs/10.1111/cogs.13020`.

[18] T. Dozat and C. D. Manning. Deep biaffine attention for neural dependency parsing. In *5th International Conference on Learning Representations, ICLR 2017, Toulon, France, April 24-26, 2017, Conference Track Proceedings*. OpenReview.net, 2017. URL `https://openreview.net/forum?id=Hk95PK9le`.

[19] A. Ferraresi, E. Zanchetta, M. Baroni, and S. Bernardini. Introducing and evaluating ukWaC , a very large web-derived corpus of english. 2008.

[20] S. Frank and P. Ernst. Judgements about double-embedded relative clauses differ between languages. *Psychological Research*, 83:1581 – 1593, 2018.

[21] L. Frazier. Syntactic complexity. In D. R. Dowty, L. Karttunen, and A. M. Zwicky, editors, *Natural language parsing: Psychological, computational, and theoretical perspectives*, pages 129–189. Cambridge University Press New York, 1985.

[22] R. Futrell, E. Gibson, and R. Levy. Lossy-context surprisal: An information-theoretic model of memory effects in sentence processing. *Cognitive Science*, 44, 2020.

[23] E. Gibson. The dependency locality theory: A distance-based theory of linguistic complexity. In Y. M. A. Marantz and W. O'Neil, editors, *Image, Language, Brain*, pages 95–126. 2000.

[24] E. Gibson and J. Thomas. Memory limitations and structural forgetting: The perception of complex ungrammatical sentences as grammatical. *Language and Cognitive Processes*, 14(3):225–248, 1999.

[25] M. Gimenes, F. Rigalleau, and D. Gaonac'h. When a missing verb makes a French sentence more acceptable. *Language and Cognitive Processes*, 24:440 – 449, 2009.

[26] M. Glanzer and N. L. Bowles. Analysis of the word-frequency effect in recognition memory. *Journal of Experimental Psychology: Human Learning & Memory*, 2:21–31, 1976.

[27] Y. Goldberg. *Neural Network Methods for Natural Language Processing*. Synthesis Lectures on Human Language Technologies. Morgan & Claypool Publishers, 2017. doi: 10.2200/S00762ED1V01Y201703HLT037. URL `https://doi.org/10.2200/S00762ED1V01Y201703HLT037`.

[28] A. Goodkind and K. Bicknell. Local word statistics affect reading times independently of surprisal. *CoRR*, abs/2103.04469, 2021. URL `https://arxiv.org/abs/2103.04469`.

[29] A. Gorman. Recognition memory for nouns as a function of abstractness and frequency. *Journal of experimental psychology*, 61:23–9, 1961.

[30] M. Hahn and R. Futrell. Estimating predictive rate-distortion curves via neural variational inference. *Entropy*, 21(7):640, 2019.

[31] M. Hahn and F. Keller. Modeling Human Reading with Neural Attention. In *Proceedings of EMNLP*, 2016.

[32] J. M. Hammersley and K. W. Morton. Poor man's Monte Carlo. *Journal of the royal statistical society series b-methodological*, 16:23–38, 1954.

[33] C. Hansen, C. Hansen, S. Alstrup, J. G. Simonsen, and C. Lioma. Neural speed reading with structural-jump-LSTM. *arXiv preprint arXiv:1904.00761*, 2019.

[34] J. Häussler and M. Bader. An interference account of the missing-VP effect. *Frontiers in Psychology*, 6:766–766, 2015.

[35] S. Hochreiter and J. Schmidhuber. Long short-term memory. *Neural Comput.*, 9(8):1735–1780, 1997. doi: 10.1162/neco.1997.9.8.1735. URL `https://doi.org/10.1162/neco.1997.9.8.1735`.

[36] M. D. Homan and A. Gelman. The no-U-turn sampler: adaptively setting path lengths in hamiltonian monte carlo. *Journal of Machine Learning Research*, 15(1):1593–1623, 2014.

[37] R. Huddleston and G. K. Pullum. *The Cambridge Grammar of the English Language*. 2002.

[38] R. Jackendoff. *X Syntax: A Study of Phrase Structure*. 1980.

[39] D. P. Kingma and M. Welling. Auto-encoding variational Bayes. In *ICLR 2014 : International Conference on Learning Representations (ICLR) 2014*, 2014.

[40] M. Lee, T. B. Hashimoto, and P. Liang. Learning autocomplete systems as a communication game. *arXiv preprint arXiv:1911.06964*, 2019.

[41] G. Leech. 100 million words of English: The British national corpus (BNC). *Second Language Research*, 28:1–13, 1992.

[42] T. Lei, R. Barzilay, and T. Jaakkola. Rationalizing neural predictions. *arXiv preprint arXiv:1606.04155*, 2016.

[43] D. Lewandowski, D. Kurowicka, and H. Joe. Generating random correlation matrices based on vines and extended onion method. *Journal of Multivariate Analysis*, 100(9):1989–2001, 2009.

[44] R. L. Lewis and S. Vasishth. An activation-based model of sentence processing as skilled memory retrieval. *Cognitive Science*, 29(3):375–419, 2005.

[45] T. Luong, H. Pham, and C. D. Manning. Effective approaches to attention-based neural machine translation. In L. Màrquez, C. Callison-Burch, J. Su, D. Pighin, and Y. Marton, editors, *Proceedings of the 2015 Conference on Empirical Methods in Natural Language Processing, EMNLP 2015, Lisbon, Portugal, September 17-21, 2015*, pages 1412–1421. The Association for Computational Linguistics, 2015. doi: 10.18653/v1/d15-1166. URL `https://doi.org/10.18653/v1/d15-1166`.

[46] S. Marzen and J. Crutchfield. Predictive rate-distortion for infinite-order markov processes. *Journal of Statistical Physics*, 163:1312–1338, 2016.

[47] B. McElree. Sentence comprehension is mediated by content-addressable memory structures. *Journal of Psycholinguistic Research*, 29(2):111–123, 2000.

[48] B. McElree, S. Foraker, and L. Dyer. Memory structures that subserve sentence comprehension. *Journal of Memory and Language*, 48(1):67–91, Jan. 2003. ISSN 0749596X. doi: 10.1016/S0749-596X(02)00515-6. URL `http://linkinghub.elsevier.com/retrieve/pii/S074959602005156`.

[49] Y. Miao and P. Blunsom. Language as a latent variable: Discrete generative models for sentence compression. *arXiv preprint arXiv:1609.07317*, 2016.

[50] A. Mnih and K. Gregor. Neural variational inference and learning in belief networks. In *Proceedings of The 31st International Conference on Machine Learning*, pages 1791–1799, 2014.

[51] B. Nicenboim, P. Logacev, C. A. Gattei, and S. Vasishth. When high-capacity readers slow down and low-capacity readers speed up: Working memory and locality effects. *Frontiers in Psychology*, 7, 2016.

[52] D. Parker, M. Shvartsman, and J. A. Van Dyke. The cue-based retrieval theory of sentence comprehension: New findings and new challenges. In L. Escobar, V. Torrens, and T. Parodi, editors, *Language processing and disorders*, pages 121–144. Cambridge Scholars Publishing, Newcastle, 2017.

[53] A. Paszke, S. Gross, F. Massa, A. Lerer, J. Bradbury, G. Chanan, T. Killeen, Z. Lin, N. Gimelshein, L. Antiga, A. Desmaison, A. Köpf, E. Yang, Z. DeVito, M. Raison, A. Tejani, S. Chilamkurthy, B. Steiner, L. Fang, J. Bai, and S. Chintala. Pytorch: An imperative style, high-performance deep learning library. In *NeurIPS*, 2019.

[54] P. Qi, Y. Zhang, Y. Zhang, J. Bolton, and C. D. Manning. Stanza: A python natural language processing toolkit for many human languages. In *ACL*, 2020.

[55] N. Rasmussen and W. Schuler. Left-corner parsing with distributed associative memory produces surprisal and locality effects. *Cognitive science*, 42:1009–1042, 2018.

[56] M. Seo, S. Min, A. Farhadi, and H. Hajishirzi. Neural speed reading via skim-RNN. *arXiv preprint arXiv:1711.02085*, 2017.

[57] A. Staub, F. Foppolo, C. Donati, and C. Cecchetto. Relative clause avoidance: Evidence for a structural parsing principle. *Journal of Memory and Language*, 98:26–44, 2018.

[58] S. Still, J. Crutchfield, and C. J. Ellison. Optimal causal inference: estimating stored information and approximating causal architecture. *Chaos*, 20 3:037111, 2010.

[59] I. Sutskever, O. Vinyals, and Q. V. Le. Sequence to sequence learning with neural networks. In *NIPS*, 2014.

[60] N. Tishby, F. C. Pereira, and W. Bialek. The information bottleneck method. *arXiv preprint physics/0004057*, 2000.

[61] S. Vasishth, K. Suckow, R. L. Lewis, and S. Kern. Short-term forgetting in sentence comprehension: Crosslinguistic evidence from verb-final structures. *Language and Cognitive Processes*, 25(4): 533–567, 2010.

[62] A. Vaswani, N. Shazeer, N. Parmar, J. Uszkoreit, L. Jones, A. N. Gomez, Ł. Kaiser, and I. Polosukhin. Attention is all you need. In *Advances in Neural Information Processing Systems*, pages 5998–6008, 2017.

[63] Y. Wang, J. M. L. Ribeiro, and P. Tiwary. Past-future information bottleneck for sampling molecular reaction coordinate simultaneously with thermodynamics and kinetics. *Nature Communications*, 10, 2019.

[64] E. Wilcox, J. Gauthier, J. Hu, P. Qian, and R. Levy. On the predictive power of neural language models for human real-time comprehension behavior. In S. Denison, M. Mack, Y. Xu, and B. C. Armstrong, editors, *Proceedings of the 42th Annual Meeting of the Cognitive Science Society - Developing a Mind: Learning in Humans, Animals, and Machines, CogSci 2020, virtual, July 29 - August 1, 2020*. cognitivesciencesociety.org, 2020. URL `https://cogsci.mindmodeling.org/2020/papers/0375/index.html`.

[65] R. J. Williams. Simple statistical gradient-following algorithms for connectionist reinforcement learning. *Machine Learning*, 8:229–256, 1992.

[66] A. W. Yu, H. Lee, and Q. V. Le. Learning to skim text. *arXiv preprint arXiv:1704.06877*, 2017.
